# Unravelling the resilience of the KGK VI population from the Gumelnița site (Romania) through stable isotopes

**DOI:** 10.1038/s41598-023-35129-0

**Published:** 2023-05-25

**Authors:** Ana García-Vázquez, Adrian Bălășescu, Gabriel Vasile, Mihaela Golea, Valentin Radu, Vasile Opriș, Theodor Ignat, Mihaela Culea, Cristina Covătaru, Gabriela Sava, Cătălin Lazăr

**Affiliations:** 1 ArchaeoSciences Platform, Research Institute of the University of Bucharest (ICUB), University of Bucharest, Bucharest, Romania; 2 “Vasile Pârvan” Institute of Archaeology, Romanian Academy, Bucharest, Romania; 3 National Museum of Romanian History, Bucharest, Romania; 4 Bucharest Municipality Museum, Bucharest, Romania; 5 “Francisc J. Rainer” Institute of Anthropology, Romanian Academy, Bucharest, Romania; 6 Horia Hulubei National Institute for R&D in Physics and Nuclear Engineering, Măgurele, Romania

**Keywords:** Anthropology, Archaeology, Biogeochemistry

## Abstract

The Gumelnița site belongs to the Kodjadermen-Gumelnița-Karanovo VI (KGK VI) communities (c. 4700–3900 cal BC) and comprises the tell-type settlement and its corresponding cemetery. This paper reconstructs the diet and lifeways of the Chalcolithic people in the northeastern Balkans using archaeological remains found at the Gumelnița site (Romania). A multi-bioarchaeological investigation (archaeobotany, zooarchaeology, anthropology) was conducted on vegetal, animal, and human remains, alongside radiocarbon dating and stable isotope analyses (δ^13^C, δ^15^N) of humans (n = 33), mammals (n = 38), reptiles (n = 3), fishes (n = 8), freshwater mussels shells (n = 18), and plants (n = 24). According to the results of δ^13^C and δ^15^N values and FRUITS, the inhabitants of Gumelnița had a diet based on crops and using natural resources, such as fish, freshwater molluscs and game. Although domestic fauna was occasionally exploited for meat, it had a role in providing secondary products. Crops were heavily manured, and chaff and other crop waste may have been necessary fodder for cattle and sheep. Dogs and pigs fed on human waste, although the diet of the latter is more similar to that of wild boars. Foxes had a diet close to dogs, which may indicate synanthropic behaviour. Radiocarbon dates were calibrated with the percentage of freshwater resources obtained by FRUITS. As a result, the corrected dates for the freshwater reservoir effect (FRE) have a delay of an average of 147 years. According to our data, this agrarian community developed a subsistence strategy under the pressure of some climatic changes that started after 4300 cal BC, corresponding to KGK VI rapid collapse/decline episode tracked recently (that begins around 4350 cal BC). This matching of our data in the two models (climatic and chrono-demographic) allowed us to capture the economic strategies that led to the resilience of those people more than other contemporary KGK VI communities.

## Introduction

**Figure 1:**
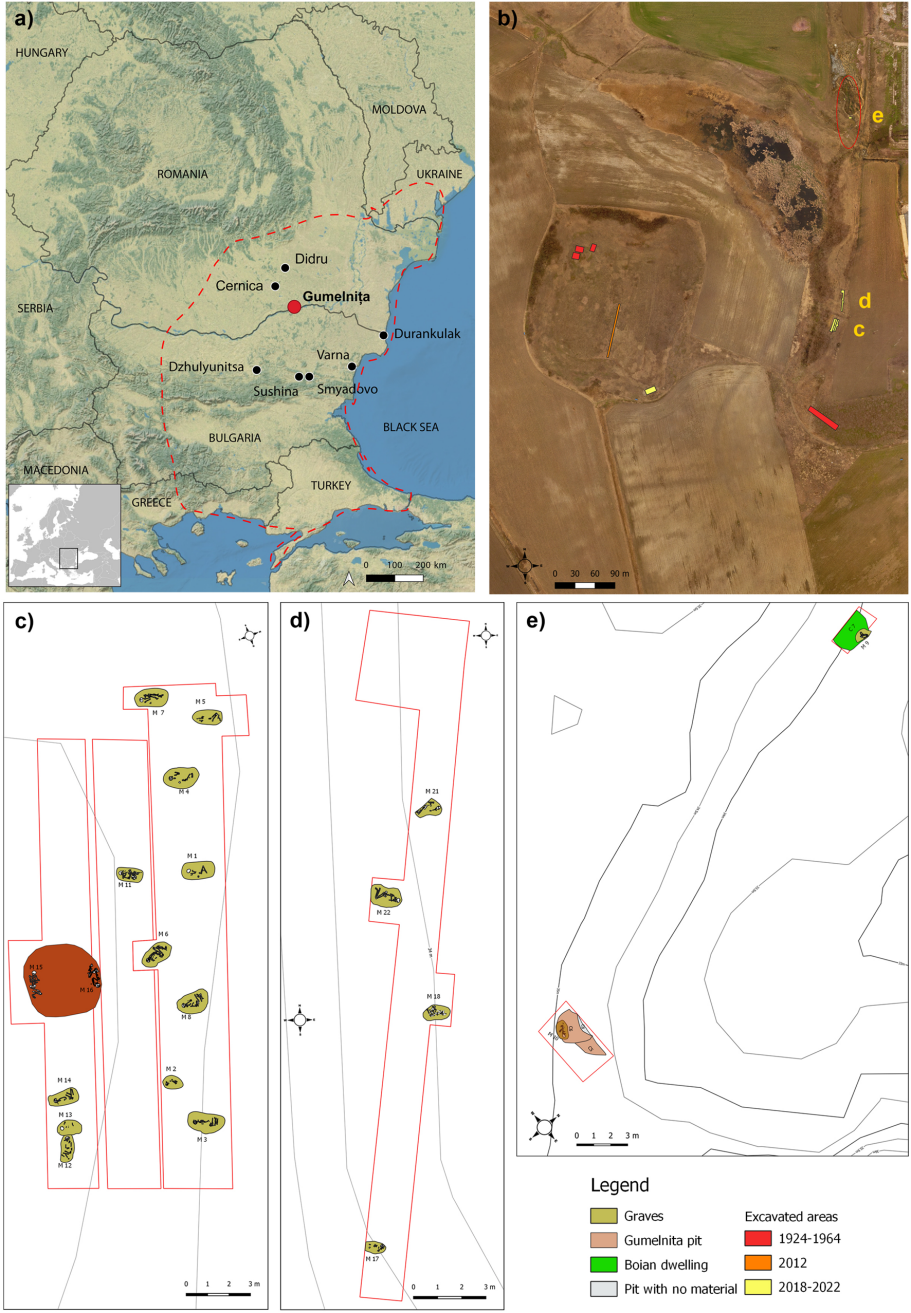
(**a**) Map showing the location of the Gumelnița site and others mentioned in the text. The maximum spread of KGK VI communities is outlined by the dotted line. This map was created using QGIS 3.20.1-Odense (https://qgis.org). Basemap source: ESRI. (**b**) Aerial picture showing excavation areas and the location of the graves excavated between 2017 and 2022 (**c**–**e**). (**c**) Graves M1-M8, M11-M16 and C12. (**d**) Graves M17, M18, M21 and M22. (**e**) Graves M9 and M10.

The Kodjadermen–Gumelnița–Karanovo VI (KGK VI) archaeological group reflects the maximum flourishing development of human communities in the Balkans during the Prehistory^1–3^. These people and their material culture features (archaeological artefacts) cover most of the northern Balkans (Fig. 1a) between c. 4700–3900 cal BC. This period is known as Chalcolithic, Copper Age or Eneolithic, representing a distinct period with substantial socio-economic transformations in the target area. Generally, these KGK VI communities occupied a vast geographical area (Fig. 1a) with diverse landscapes and ecosystems, ranging from mountains and hills, to plains, wetlands and costal environments, and from riparian ecosystems to the steppe.

### KGK VI archaeological and paleogenetic framework

Around 5000 cal BC, in the northern Balkans (Bulgaria and Romania), some fundamental changes took place, such as the rise of tell settlements, the development of the defensive systems—ditches, banks and palisades, the emergence of extramural cemeteries, adoption of some new raw materials—copper, gold, graphite alongside metallurgical innovation. The KGK VI people's lifestyle determined by these innovations and inherent technological progress, led to the development of complex and stratified societies very well reflected by the wealthy graves documented in some extramural cemeteries^1–4^.

Following the *classic* cultural-historical interpretation, most archaeologists still consider that KGK VI originated from the evolution of previous archaeological "cultures" in Bulgaria and Romania (e.g., Boian, Maritsa, Polyanitsa, Karanovo V and Hamangia)^4^. However, this significant development of KGK VI society could also result from a slow migration process from the Southeast. Recent aDNA analysis demonstrated that both populations (Boian and Gumelnița) have similar genetic signatures due to a common origin in the Northwestern Anatolia from where they arrived in the Balkans^5–7^. This implies that the so-called “cultures” from Northeastern Bulgaria and Southeastern Romania, along with all known innovations, social development, and material cultural progress, were the result of these newcomers, who came with new ideas and managed to occupy the area of the Northern Balkans, a region previously inhabited only sporadically. Newcomers arrived in the northern Balkans around 4901–4751 cal BC, and in the first 300 years, the human population steadily grew, reaching a maximum development and territorial expansion between 4500 and 4400 cal BC; then quickly declined until it vanished from archaeological records around 4150–3800 cal BC^8^.

From a paleogenomic perspective, this KGK VI people present typical Northwestern Anatolian Neolithic-related ancestry. Also, this population presents some Balkan hunter-gather related ancestry^9^, along with sporadic evidence of steppe-related ancestry (Varna I and Smyadovo)^6^, which points towards a genetic admixture between these populations. This interplay of the local, steppe, and western Asian ancestries in Southeastern Europe Chalcolithic people mark the end of the replacement of its local Balkan hunter-gatherers by Anatolian Neolithic farmers, a process that began ~ 8500 years ago^7^.

This admixture could probably explain some variabilities observed between KGK VI people, at the level of material culture, diet and lifestyle, a situation that could reflects by their different origins, and, consequently, various cultural traditions^8^.

### Gumelnița site

The notoriety of the Gumelnița site is linked to its tell settlement, probably the largest tell settlement of KGK VI people north of the Danube (Fig. S1a). Nearby, the pair cemetery of the KGK VI settlement is documented, along with earlier Neolithic habitation traces (Boian “culture”), and a Cernavodă II cemetery, a Bronze Age settlement (Tei "culture"), and sporadic features corresponding to the first Iron Age and the seventh–eleventh centuries CE^10^. Only the features assigned to the KGK VI and Boian archaeological groups were radiocarbon dated.

#### Location

The Gumelnița eponymous site is located in the northern area of the Balkan region (Fig. 1a), in Southeast Romania, on the left bank floodplain of the Danube River, immediately south of the confluence of the Argeş River with a small tributary (Valea Mare)^10–15^.

#### Excavation biography

The history of this site is linked to the beginnings of the archaeological discipline in Romania. The first archaeological research at this site layed the foundations for the definition of Gumelnița “culture”^16–18^. The mound from “Măgura Gumelnița” or “Măgura Calomfirescu” (the tell settlement) has been mentioned since the nineteenth century, and at the beginning of the twentieth century was the first time investigated archaeologically by Vladimir Dumitrescu (1925). In the interwar period, Dinu V. Rosetti (Bucharest Municipality Museum) and Barbu Ionescu (Oltenița Local Museum) will periodically investigate the site, and in the 60's the excavations will be resumed by a team led by Vladimir Dumitrescu and Silvia Marinescu-Bîlcu. After that, the site would be sporadically investigated by the Oltenița Museum's archaeologists (Călărași County). In 1977, on the terrace (cemetery area) a rescue excavation led to the identification of new graves. Between 2011 and 2012 the site was investigated (partially) again under the umbrella of another rescue excavation. In 2017 our team resumed the archaeological investigation, and since then, research has continued every year except for the lockdown period^10,11,13–16,18^.

#### Tell settlement

The tell mound is located in an erosional remnant from the high terrace of the Danube on the loess deposits^11^. Although from the ground level, it looks like an elongated mound, the aerial photos show that it has a rectangular shape (Figs. 1b and S1b). The erosional remnant has a length of 272 m and a width of 227 m; the maximum elevation is 38 m.a.s.l, and the area is about 60,000 m^2^.

Our excavation area is located in the Southeastern corner of the mound (Fig. 1b), not in the top area, where the 2011–2012 rescue trench excavation identified an intense KGK VI habitation.

The archaeological stratigraphy has a thickness of over 4 m (in the top area) and more than 5 m (our excavation), and all anthropic deposits belong to the Gumelnița archaeological signals (A1, A2 and B1 phases or ceramic styles). The absolute chronology of the tell settlement based on the radiocarbon dating of bones, charcoal and seeds (n = 14) is 4800 through 4040 cal BC with a 95.4% probability^10^ (Table S8).

The KGK VI features identified in our investigations (2017–2021) consist of two burnt and two unburnt houses (features L1, L2, and L3, L4 respectively), some pits (features C1, C2, C3, C5, and C6), and a waste area (feature C4). The investigated features contain different quantities of pottery, flint tools, grinding stones, clay figurines, metal items, and adornments along with animal or plant remains^10,11,19^. Some of the recovered artefacts (e.g., grinding stones, stone crusher and flint sickle implements) indicate the agricultural activities practised by the Gumelnița people. In addition, there are documented tools related to hunting (e.g., arrowheads and spearheads) and fishing (e.g., clay weights for fishing nets, fishing hooks or harpoons), but also other tools and recipients used for domestic activities (e.g., pots of various dimensions used for cooking, serving or storing the food, stone or flint axes, clay weights for weaving)^10,11,19^.

#### Cemetery

The cemetery was identified at the end of the 50's of the 20th century, approximately 200 m East of the tell settlement, when some public works destroyed 7–8 graves. Between 1961 and 1962, in the same area, 5 inhumation graves were examined. In 1977, a rescue excavation led to the identification of other 3 graves. The graves contain skeletons in a crouched position on the left side. Only the individual from grave M3/1962 was in extended dorsal position, a typical burial position for the KGK VI communities that lived on the Black Sea coast (Palazu Mare, Varna I, Durankulak, and Devnja cemeteries)^20^. In most cases, the individuals had been buried with the head positioned towards east. Our work led to the identification of additional 20 inhumation graves (2017–2022) (Figs. 1c–e, S2, S3 and S4). The skeletons have approximately the same position and orientation as in previously investigated burials in this KGK VI cemetery^15,21^. Based on the radiocarbon dates performed on human bones (n = 10), the cemetery’s period of use can be estimated to 4545–4179 cal BC (95.4% probability)^10^. The contemporaneity of the individuals buried in the cemetery is proved by ^14^C dates obtained for the features investigate on the tell settlement (Table S8).

The grave goods are presented only in a few graves (n = 11) and consist of stone or copper axes, flint blades, bone figurines, copper chisel or shell beads^10,11,15,19,22^ (Table 1). In some graves, a variety of plant remains were identified (Table S1).

**Table 1:** List of the graves from the Gumelnița site, with the indication of sex, age, pathology, and grave goods.

Grave	Year	Sex	Age	Pathology	Grave goods	References
M1-I1*	2017	F	33–45 years (Middle adult)	–	–	^11^
M1-I2	2017	–	Adolescent	–		^11^
M2-I1*	2018	–	Infant/child (~ 3 years)	Periostitis (femoral and tibial diaphysis)	–	^23^
M2-I2	2018	–	Adult	–		^23^
M2-I3	2018	–	Adult	–		^23^
M3-I1*	2018	F	34.7 ± 7.8 years (Young adult)	Antemortem tooth loss (M_1_ right)	–	^23^
M3-I2	2018	–	Child (Infans I) (3–5 years )	Periostitis (femoral diaphysis)		^23^
M4	2018	F	< 30.5 years (young adult)	Paget's disease (*osteitis deformans*)	–	^23^
M5	2018	F	Adult	Periostitis (femoral and tibial diaphysis)	–	^23^
M6	2018	M	51.5 ± 12.6 years (old adult)	Periostitis, antemortem tooth loss (inferior molars)	–	^23^
M7	2018	M	Young adult	–	–	^23^
M8	2018	M	30.5–32 years (young adult)	–	Pot, flint, *Spondylus* sp.beads, pigments	^23^
M9	2018	F	Adult	Periostitis (tibial diaphysis)	Flint, ceramic (fragments)	^23^
M10	2018	F	48.8 ± 10.5 years (middle adult)	Dental caries (M_1_–M_3_ left); antemortem tooth loss (M_1_–M_3_ right); osteoarthritis	–	^23^
M11	2019	M	45.6–54.5 years (old adult)	Antemortem tooth loss; cranial traumas; osteoarthritis	Pot, pigments	^10^
M12	2019	F	48.8 years (middle adult)	Dental caries	*Antalis* sp. beads, red ochre traces on the skull	^10^
M13	2019	–	1–3 years (Infant)	Periostitis	Anthropomorphic figurine, *Antalis* sp*.* beads	^10^
M14	2019	F	32–34.7 years (young adult)	–	*Spondylus* sp. beads, flint	^10^
C12-I1	2019	M	Adult	Dental calculus	Ceramic fragments, charcoal, animal bones (*Bos taurus, Ovis aries, Capra hircus, Sus domesticus, Canis familiaris*)	^19^
C12-I2	2019	–	1 year ± 4 months	–		^19^
C12-I3	2019	–	Adult	–		^19^
C12-I4	2019	–	Adult	–		^19^
C12-I5	2019	–	Adolescent	–		^19^
C12-I6	2019	–	Adolescent	Periostitis (femur diaphysis)		^19^
M15	2021	F	31.2 (22.6–38.2) (young adult)	Dental calculus	–	^19^
M16	2021	F	49.7 (39.4–59.9) (old adult)	Antemortem tooth loose (P^2^ bilateral, inferior molars)	–	^19^
M17	2022	F	Young adult	–	–	**
M18	2022	F	Young adult	–	–	**
M21	2022	M	Young adult	–	–	**
M22	2022	M	Young adult	–	Flint, red pigment	**
M1	1962	M	30–35 years (young adult)	Osteophyte formation in two thoracic bodies; osteophyte formation in distal epiphysis of the left fibula	–	**,^15^
M2	1962	M	30–32 years (young adult)	Humerus varus deformity (unilateral, right)	–	**,^15^
M3	1962	M	26–32 years (young adult)	Criba orbitalia; fracture located in the proximal third of the right humerus (oblique fracture, healed, without signs of infection); trauma located in the left parietal (healed); eburnation; markedly accentuated longitudinal striations and discrete paths of reactive bone, on both tibias	Ceramic (fragments), animal bones (no sp. info)	**,^15^
M1-I1*	1960	–	Child (4–6 years)	cribra orbitalia (both orbits, bilateral pitting), cribra cranii (bilaterally, parietal, located superior to lambdoid suture)	Ceramic (fragments), cereal grains, ash, animal bones (*Bos taurus*, *Silurus glanis* and *Emys orbicularis*) and a human vertebra (M1-1960-I2)	**,^24^
M1-I2	1960	–	Adult	–		**

“M” indicates the grave and “I” is the individual. *Denotes de main individual of the grave. ** this paper.

In 3 of the burials (M1, M2, M3) and in C12 pit, human bone fragments which belong to other individuals than those buried in anatomical positions were found. This particular custom fits well in the *deviant burials* category (addition type) that involves the deliberate incorporation of human bones from another skeleton into a burial of a more or less complete burial^25^. The situation from Gumelnița cemetery is not a exceptional one, being also documented in the Sultana-Malu Rosu cemetery (Călărași County, Romania) and other KGK VI necropolises^26^. In the rest of the graves, only single individuals were found buried.

#### Animal and plant remains

Both plant and animal remains (Tables S1 and S2) from the Chalcolithic period were found on the site^10,11,19^. Unfortunately, the data regarding faunal and botanical findings from 60's graves are not available (Table 1). Within the mammal remains, domestic animals are the dominant (89.87%), and within them, the most numerous are the cattle (*Bos taurus*: 41.47%), followed by ovicaprines (*Ovis aries*/*Capra hircus*: 32.73%), pigs (*Sus domesticus:* 9.06%) and dogs (*Canis familiaris:* 6.61%). Game is 5.54% and is represented by: wild horse (*Equus ferus*), auroch (*Bos primigenius*), red deer (*Cervus elaphus*), roe deer (*Capreolus capreolus*), wild boar (*Sus scrofa*), fox (*Vulpes vulpes*), european badger (*Meles meles*), hare (*Lepus europaeus*) and beaver (*Castor fiber*). The 1966 study^27^, also concludes that cattle prevail, followed by ovicaprines and pigs, while hunting is poorly represented.

After mammals, molluscs are the animal group with a more significant number of remains. The generus *Unio* (*U. tumidus, U. pictorum, U. crassus* and *Unio *sp.) represents 63.06%, followed by *Anodonta* sp. (30.07%) and other species (*Pseudoanodonta* sp., *Viviparus* sp*. Dreissena* sp*.* and *Cepaea vindobonensis*). The fish with a greater amount of remains are the eurasian carp (*Cyprinus carpio*) (30.61%) and the catfish (*Silurus glanis*) (30.61%), followed by the northern pike (*Esox lucius*) (8.16%). Remains of unidentified species of fishes represent 30.61%. The Reptilia remains belong to the pond turtle (*Emys orbicularis*). Only four bird bones were identified (3 from small individuals, 1 from a large individual).

Moreover, a large diversity of plants was identified at least at family level^10,11^, and most of them were recovered from the tell settlement. Most macrobotanical remains were identified at the family level. The cereal remains are the most diverse (and larger batch), with wild and domesticated species. The wild herbaceous cereals species are brome grass (*Bromus* sp.) and needle grass (*Stipa* sp.), while the domestic cereals are einkorn wheat (*Triticum monococcum*), emmer wheat (*Triticum dicoccum*), barley (naked and hulled) (*Hordeum vulgare*), and spelt wheat (*Triticum* cf. *spelta*).

Pulses are also an important food source documented through both domestic (lentil (*Lens culinaris*), pea (*Pisum* sp.) and bitter vetch (*Vicia ervilia*)), and wild species like vetchling (*Lathyrus* sp.).

Among the fruits, *Prunus* sp. stands out because of the numerous fragments of the stone (pyrena) recovered. In some cases it has been possible to reach the species level, thus identifying the cherry plum (*Prunus cerasifera*). Other wild fruits found were acorn (*Quercus robur pedunculiflora*), elderberry (*Sambucus nigra*) and blackberry (*Rubus* cf. *fruticosus*). The vegetation signature is completed by some herbaceous plants (*Brassica* sp., black bindweed (cf. *Fallopia convolvulus*), fat hen (*Chenopodium album*), false cleaves (*Galium* cf. *spurium*), pale persicaria (*Persicaria lapathifolia*), common knotgrass (*Polygonum aviculare*), common sorrel (*Rumex acetosa*) and water pepper (*Persicaria hydropiper*)).

The full archaeozoological and archaeobotanical data from the site are described in Lazăr et al.^10,19^. Tables S1 and S2 indicate the number of remains and the minimum number of individuals.

### Stable isotopes

#### Stable isotopes biogeochemistry as a tool for reconstructing the past

Stable isotope analyses are a standard analytical tool employed in several scientific areas, but also in archaeology in reconstructing components of the dietary system and multiple historical and cultural issues associated with this system^28,29^. The basic principles of this technique are relatively simple: both carbon and nitrogen occur in nature in two main isotopes, one lighter and more abundant, and the other heavier and scarcer. The ratio of light to heavy isotopes of an element will vary depending on physical–chemical, thermal and biological reactions, which is known as isotope fractionation. This fractionation occurs, for example, between human’s diet and its own isotopic values, being a suitable tool for the reconstruction of the diet of past organisms. The proportion between the light and heavy isotopes of carbon and nitrogen in bone collagen, allows the identification of the origin of the proteins assimilated by the individual, since each large food group has its characteristic isotopic values^30,31^. In this way, it is possible to differentiate foods of terrestrial origin from marine or freshwater using their carbon isotope ratios, since the terrestrial food chain is based on atmospheric CO_2_ that plants fix by photosynthesis, while in the aquatic environments carbon derives from carbonate species dissolved in water^32–34^.

In the case of terrestrial plants, we can differentiate C4 plants, which are tropical herbaceous plants among which some are of great interest in human nutrition (millet, corn, sorghum, sugar cane) and have a clearly different carbon isotope signal than C3 plants^35,36^. For example, the values of δ^13^C in C3 plants vary between − 30 and − 21‰ with an average of − 26.5 ± 2‰; C4 plants vary between − 15 and − 7‰ with an average of − 12.5 ± 1‰^37,38^. Among C3 plants, leguminous plants also show a different isotopic signal from the rest, in this case, the nitrogen signal, since they fix N_2_ directly from air, thanks to structures called mycorrhizae, and not from the nitrates produced by soil bacteria^39^.

In addition a constant offset in nitrogen isotope values is produced by processes related to the assimilation of food. At each food chain step the nitrogen isotope values increase by 3.5–5‰^31,40^. Nitrogen isotopes also help to determine whether the food source came from terrestrial or aquatic ecosystems because in the latter, the food web is more complex and nitrogen isotope values are overall higher^33^. The carbon signal also varies: between a plant and the bone collagen of the herbivore that consumes it, the increase is 5‰; after the primary consumers, each step in the food chain correspond an additional increase in δ^13^C values of up to 1‰^40^.

Bone collagen represents 90% of the entire organic fraction of bone, and it is formed from the proteins ingested in the diet. It renews itself very slowly, which makes it possible to reconstruct the diet of humans for at least the last 10 years of their lives^30^. For other mammals, the turnover rate can be higher, like in dogs, where the rate among young adult dogs is between 6 months and 3 years^41^.

Conchiolin is the acid-insoluble fraction of the mollusc shell organic matrix and is proteinaceous, so it can be used as a proxy for the soft tissues in isotopic analyses^42–44^. The values obtained in the conchiolin are similar to those of the animal's body^42^, which represents an approximation of past diets. So far, only marine molluscs have been analysed to study the diets of past humans^45–48^.

Carbonized plant materials (combusted under poor oxygen availability) prevent the degradation by microorganisms and their morphological traits are still recognisable. Because of this, wood charcoal and charred seeds and grains are the most common archaeobotanical remains. Studies on modern seeds concluded that there is only a slight change in δ^13^C and δ^15^N due to carbonization^49^. The stable isotopes of the seeds reflect the conditions where the plants grew. The analysis of carbon isotope discrimination (Δ^13^C) in crop plant remains from archaeological sites is positively correlated with water availability during the period of growth^50–52^. The addition of manuring to the fields can raise the natural δ^15^N of soils, resulting in crops with higher δ^15^N than unfertilised soil^53^.

For the reconstruction of the contribution of the different types of foods in the diet of humans or animals statistical tools have been developed such as Bayesian mixing models^54,55^. FRUITS (Food Reconstruction Using Isotopic Transferred Signals)^56,57^ is probably the most used in archaeology and helps to achieve accurate and precise food intake estimates in diet reconstruction studies.

#### Stable isotopes studies in the KGK VI area

Unfortunately, only a few studies using stable isotope analysis are available in the targeted timespan from the northern Balkans. Varna I and Durankulak cemeteries are the most extensively studied sites in the KGK VI area. The first isotopic results suggested that both populations primarily utilised terrestrial C3-based diets with varying proportions of meat, despite their proximity to the Black Sea^58,59^, but later a small percentage of marine sourced protein was identified^60,61^. Mathieson et al.^6^ published stable isotope data together with ancient DNA and radiocarbon dating from Dzhulyunitsa, Smyadovo, and Sushina sites that belong to the KGK VI area in Bulgaria, but those isotopic results haven’t been interpreted yet. The same happened with the data from Tafani et al.^62^ from the site of Dridu in Romania.

Regarding domestic animals, cattle were dominant during the Neolithic and also in the KGK VI period^63,64^, followed by sheep, goats, and pigs. The cattle mortality profile suggests husbandry oriented towards prime meat exploitation (6–24 months) and dairy production (4–9 years)^65^. The presence of dairy lipid residues in ceramic pots provides direct evidence of this, at least from the sixth millennium BC in the Balkans^66,67^. For cattle, winter leaf foddering was suggested^68^. Some of the sheep and cattle from KGK VI show δ^13^C values compatible with a minor consumption of C4 plants^65,69,70^, and it has been proposed seasonal exploitation of salt marshes in the Black Sea coastal area.

As for pigs, their hybridisation with wild boar^71^ makes it necessary to resort to bone metrics to differentiate them. Even so, isotopic interpretation is not easy. However it seems common for wild boars to have lower δ^15^N values on average, although the distributions of the two overlap^72^, but the higher contribution of animal protein in pigs' diet suggests that it would be provided by humans and that those pigs would be maintained in the settlement^70^.

This study aims to reconstruct the diet of the ancient inhabitants of the Gumelnița site and to reconstruct the lifestyle of the KGK VI people that lived here ~ 6000 years ago through the stable isotope analysis of bone collagen, conchiolin extracted from bivalve shells and charred seeds and grains, all recovered from Gumelnița site.

## Material and methods

### Plants

A total of 24 charred grains/seeds or fragments have been analysed for stable isotopes (Table 2). Grains and seeds analysed here come from our excavations from the years 2017–2020. The domestic plants analysed are emmer wheat (n = 3), einkorn wheat (n = 3), barley (n = 4), lentil (n = 1), pea (n = 2) and bitter vetch (n = 1). The wild seeds and grains analysed are from *Prunus* sp. (4 fragments coming from 2 different pits), greyish oak (n = 1), blackberry (n = 2) and *Bromus* sp. (n = 2). All the plants come from pits of the KGK VI period except the bitter vetch which is from a Boian pit^10^.

**Table 2:** Isotopic results for the plants from the Gumelnița site. δ^15^N and δ^13^C values of the grain include the offset of charred grain applied of − 0.31 in δ^15^N and − 0.11 in δ^13^C^73^.

Lab ID	Taxon	Feature	Grain							Chaff	
			%N	δ^15^N (‰)	%N	δ^13^C (‰)	C: N	Δ^13^C	WI	δ^15^N (‰)	δ^13^C (‰)
GUM-54	*T. dicoccum*	C5	4.6	3.7	62.0	− 28.7	15.7	22.9	921.9	1.3	− 30.6
GUM-55	*T. dicoccum*	C5	5.4	6.6	68.9	− 22.4	15.0	16.3	84.4	4.2	− 24.3
GUM-56	*T. dicoccum*	C5	3.5	3.8	69.2	− 24.0	23.2	17.9	154.7	1.4	− 25.9
GUM-57-T	*Prunus* sp.	C6	1.1	5.0	62.6	− 24.7	69.4	18.7	–	–	–
GUM-58	*Prunus* sp.	C4	1.1	4.7	67.0	− 25.8	73.9	19.8	–	–	–
GUM-59	*Prunus* sp.	C4	1.1	3.6	57.6	− 26.1	58.5	20.1	–	–	–
GUM-60	*Prunus* sp.	C4	0.8	6.3	65.4	− 23.2	95.2	17.1	–	–	–
GUM-61-T	*Q. robur*	L1	1.7	4.0	49.2	− 27.0	33.8	21.1	–	–	–
GUM-62	*V. ervilia*	C7	6.6	1.3	63.9	− 26.7	11.4	20.8	–	–	–
GUM-63	*T. monococcum*	C4	3.4	6.1	68.5	− 25.7	23.4	19.7	294.8	3.7	− 27.6
GUM-64	*T. monococcum*	L1	6.7	6.7	67.7	− 23.5	11.8	17.4	126.0	4.3	− 25.4
GUM-65	*T. monococcum*	C5	4.8	5.1	67.0	− 23.4	16.4	17.4	124.7	2.7	− 25.3
GUM-66	*H. vulgare (nudum)*	C5	4.2	6.0	65.5	− 25.8	18.4	19.8	302.4	3.6	− 27.5
GUM-67	*H. vulgare (nudum)*	C4	3.3	13.4	70.1	− 24.5	25.1	18.5	182.5	11.0	− 26.2
GUM-68	*H. vulgare (nudum)*	C2	3.9	5.4	70.8	− 23.8	21.1	17.7	135.8	3.0	− 25.5
GUM-69	*H. vulgare (nudum)*	C6	6.5	3.9	65.6	− 26.1	11.8	20.1	338.6	1.5	− 27.8
GUM-70	*L. culinaris*	L1	6.7	8.2	60.8	− 22.8	10.6	16.7	–	–	–
GUM-71	*Pisum* sp.	L1	4.0	6.9	62.9	− 23.3	18.1	17.2	–	–	–
GUM-72	*Pisum* sp*.*	L1	4.4	7.1	60.5	− 23.4	16.2	17.3	–	–	–
GUM-73–74	*R.* cf.* fruticosus*	C6	5.6	9.3	59.8	− 27.0	12.4	21.1	–	–	–
GUM-75	cf.* Bromus*	C7	3.5	9.3	63.4	− 24.5	20.9	18.4	–	–	–
GUM-76	*Bromus* sp.	C7	3.4	10.1	63.7	− 23.9	21.7	17.8	–	–	–

Offset grain-rachis is − 2.4‰ for δ^15^N for both cereals, − 1.9‰ in wheat δ^13^C and − 1.7‰ in barley^74–76^.

To check if there are contaminants in the seeds that are naturally present in the soils and can affect the stable isotope ratios (carbonates, nitrates or humic acids) we used the methods described by Vaiglova et al.^77^ Due to the small size of the seeds, two samples with the highest weights were chosen (GUM-57 and GUM-61) for testing soil contamination. Samples were crushed and analysed by Fourier Transform Infrared Spectroscopy with Attenuate Total Reflectance (FTIR-ATR) using a Vector 22 from Bruker. Each sample was measured once, the background was subtracted and a baseline correction was performed using OPUS software. The spectra were normalized. The following peaks were observed to test the contamination: 870 and 720 for carbonates; 3300, 1450 and 1085 for nitrates and 3690, 1080 and 1010 for humic acids. Both samples presented a peak in 870 (carbonates contamination), and GUM-61 another one in 1085 (nitrate contamination) (Fig. S5). As carbonates and nitrates were present in the samples, following Vaiglova et al.^77^, the pre-treatment applied to the sample to remove this contamination was 0.5 M HCl at 80 °C for 30 min (or until effervescence stopped) followed by three rinses in ultrapure Milli-Q water. After this, samples were freeze-dried and analysed for carbon and nitrogen isotope ratios. GUM-57 (now GUM-57-T) and GUM-60 (now GUM-60-T) were again analysed by FTIR-ATR to make sure the contamination had been eliminated. Peaks 870 and 1085 were not present in this new analysis. Figure S5 from Supplementary Materials show the FTIR-ATR spectra (done with Spectragryph 1.2.15) of pre- and post-treatment. Due to the success of the pre-treatment in removing the contaminants, this protocol was used for the rest of the samples. Then samples were freeze-dried for analysis by isotope-ratio mass spectrometry (IRMS).

To correct the values due to the charring effect, we used the corrections of Nitsch et al.^73^ of − 0.31‰ for the δ^15^N and − 0.11‰ for the C.

For the calculation of carbon isotope discrimination (Δ^13^C) in C3 plants, we used the equation from Farquhar et al.^51^: $\Delta {}^{13}C=\frac{\delta {}^{13}{C}_{air}-\delta {}^{13}{C}_{p}}{\left(1+\frac{\delta {}^{13}{C}_{p}}{1000}\right)}$, where δ^13^C_air_ of the Neolithic period is − 6.5‰^78^, and δ^13^C_p_ is the plant carbon isotope composition.

Water input (WI) represents the rainfall and the irrigation (if applied) during grain filling of barley and wheat grains. For calculations, we used the equations^79^: Δ^13^C_barley_ = 9.99 + 1.52 × ln(WI) and Δ^13^C_wheat_ = 8.50 + 1.78 × ln(WI).

As domestic herbivores eat preferably cereal chaff than grain (which is mainly for human consumption), for calculating the value of the correspondent chaff, offsets of − 2.4‰ of δ^15^N for both cereals, and − 1.9‰ of δ^13^C for wheat and − 1.7‰ for barley^74–76^ were used.

### Bone collagen

A total of 33 human remains (Table 3) have been selected for isotopic analysis. The information about sex, age, pathologies and grave good can be found in Table 1. From the skeletons excavated during 2017–2022, one sample per individual was selected (except in the case of grave M2, where only M2-I1 was selected) after they were individualized during the anthropological study^11,19,23^. All of the remains belong to graves with complete skeletons, except the ones from C12, a pit with disarticulated human bones. In this case, we sampled the individuals following the identification of the anthropological study^19^.

**Table 3:** Elemental and isotopic results of bone powder and bone collagen isotopic results from the Gumelnița humans.

Lab ID	Grave	Bone	Bone powder		Bone collagen					
			%N	%C	%N	δ^15^N (‰)	%C	δ^13^C (‰)	C:N	Yield (%)
GUM-1	M1-I1	Femur	1.3	6.3	13.1	9.5	35.7	− 20.2	3.2	11.3
GUM-2	M1-I2	Femur	1.2	6.0	12.1	9.4	33.5	− 20.5	3.2	11.5
GUM-3	M2-I1	Long bone	1.5	6.4	13.2	10.1	36.0	− 19.7	3.2	4.1
GUM-4	M3-I1	Ulna	*0.4*	5.1	10.8	10.4	31.2	− 20.6	3.4	2.0
GUM-5	M3-I2	Long bone	1.7	6.8	13.5	10.3	36.7	− 19.8	3.2	11.1
GUM-6	M4	Long bone	0.8	4.7	12.2	9.5	33.0	− 20.3	3.1	7.1
GUM-7	M5	Long bone	*0.4*	3.6	11.5	9.6	30.7	− 20.4	3.1	6.3
GUM-8	M6	Long bone	*0.4*	4.5	12.0	10.0	31.5	− 20.0	3.0	6.4
GUM-9	M7	Femur	*0.4*	4.8	11.9	10.1	33.4	− 20.3	3.3	5.5
GUM-10	M8	Long bone	0.7	5.5	12.0	10.6	31.4	− 20.1	3.1	3.7
GUM-11	M9	Long bone	0.8	5.1	13.2	10.0	36.2	− 20.2	3.2	3.9
GUM-12	M10	Long bone	1.5	6.6	14.0	10.5	37.5	− 20.2	3.1	7.1
GUM-13	M11	Femur	0.5	3.9	12.4	10.2	33.3	− 20.4	3.1	3.7
GUM-14	M12	Femur	0.7	5.5	12.7	10.1	34.3	− 19.8	3.2	3.6
GUM-15	M13	Long bone	*0.3*	6.6	11.2	12.4	28.6	− 19.5	3.0	4.3
GUM-16	M14	Long bone	1.5	7.0	13.3	9.6	36.3	− 19.9	3.2	4.9
GUM-81	C12-I1	Mandible	0.7	4.6	5.9	10.9	16.1	− 20.5	3.2	8.0
GUM-82	C12-I2	Femur	1.2	5.4	8.0	14.3	22.2	− 19.3	3.2	12.5
GUM-83	C12-I3	Ulna	1.1	5.7	7.8	9.6	21.4	− 20.0	3.2	7.8
GUM-84	C12-I4	5th metatarsal	2.2	8.1	13.9	10.9	38.9	− 19.6	3.3	15.4
GUM-85	C12-I5	Fibula	0.8	4.2	9.5	8.9	26.2	− 20.4	3.2	5.5
GUM-86	C12-I6	Femur	1.0	5.0	9.7	9.0	28.6	− 20.9	3.4	4.3
GUM-87	M15	Rib	0.8	4.2	11.5	10.5	32.7	− 20.0	3.3	4.1
GUM-88	M16	Rib	1.0	4.7	12.4	9.0	34.5	− 20.3	3.2	5.0
GUM-116	M17	Rib	0.5	6.6	8.8	9.7	24.2	− 21.0	3.2	3.8
GUM-117	M18	Rib	0.7	7.0	13.7	9.6	37.5	− 20.5	3.2	7.4
GUM-120	M21	Rib	1.6	6.7	14.9	10.6	40.6	− 20.4	3.2	10.6
GUM-121	M22	Rib	1.3	6.8	14.5	10.1	39.8	− 20.2	3.2	9.4
GUM-95	M1/1962	Rib	0.6	7.3	12.4	9.5	35.4	− 20.8	3.3	4.1
GUM-96	M2/1962	Rib	1.1	9.1	13.2	11.5	36.3	− 20.6	3.2	7.6
GUM-97	M3/1962	Rib	1.2	5.7	12.3	10.5	34.3	− 20.3	3.2	8.6
GUM-101	M1/1960-I2	Vertebra	1.5	8.4	12.8	11.5	35.9	− 20.5	3.3	10.2
GUM-102	M1/1960-I1	Skull	–	–	13.3	9.6	37.0	− 20.6	3.2	10.1

In italics the %N results that are under 0.5%.

In addition to the graves investigated by us, samples were taken from the human remains recollected during the 60’s on the cemetery (M1/1962, M2/1962 and M3/1962)^15^ and the tell (M1/1960)^24^, deposited in the Francisc J. Rainer Institute of Anthropology (Bucharest, Romania). M1/1962, M2/1962 and M3/1962 samples from this old collection come from skeletons in anatomical connection (single graves), for which the bone inventory is recorded. M1/1960-I1 is a child skull discovered in the tell during the excavation of 1960 by Vladimir Dumitrescu. The skull (M1/1960-M1) was found with animal remains and a human vertebra (M1/1960-M2) that belonged to an adult. A synthesis of the new anthropological study done for these human remains excavated in the 60's can be found in Table 1.

Using this sample selection strategy, we tried to cover the entire human population available at Gumelnița site.

23 domestic mammals (6 cattle, 5 dogs, 6 sheep, 1 goat, 5 pigs), 10 wild mammals (1 auroch, 1 beaver, 1 roe deer, 1 red deer, 1 horse, 1 hare, 2 wild boars and 2 red foxes), 2 *Sus* sp. (due to their fragmentation it was not possible to classify under the wild or the domestic type), 3 reptiles (pond turtle) and 8 fishes (6 catfish and 2 eurasian carps) were selected for collagen extraction (Table 4). The selected bones come from tell features, and from the cemetery (C12 pit). Table S2 contain all zooarchaeological information, including the minimum number of individuals (MNI). Burnt and digested bones are not considered in our study (neither for ^14^C nor for stable isotopes). Only the human bone of M1-I2 shows some burning traces. Generally, the percentage of the burnt bones in the osteological batch from the Gumelnița site is very small (not more than 1%).

**Table 4:** Elemental and isotopic results of bone powder and bone collagen from Gumelnița vertebrates.

Lab ID	Feature	Species	Bone	Age	Bone powder		Bone collagen					
					%N	%C	%N	δ^15^N	%C	δ^13^C	C:N	Yield (%)
GUM-18	US 1044	Cattle	Right tibia	> 2.5 years	2.9	9.9	13.7	7.5	37.2	− 17.9	3.2	11.8
GUM-19	US 1048	Cattle	Right tibia	> 2.5 years	2.8	9.6	13.2	7.2	35.2	− 18.7	3.1	15.2
GUM-20	US 1053	Cattle	Right tibia	< 1 years	2.6	9.0	13.6	7.6	36.3	− 19.1	3.1	13.6
GUM-21	US 1036	Cattle	Right tibia	< 1 years	2.6	9.2	14.2	7.6	38.1	− 18.9	3.1	3.6
GUM-91	US 1060	Cattle	Right tibia	> 2 years	2.8	10.2	13.5	6.3	37.5	− 19.7	3.2	15.1
GUM-100	M1/1961	Cattle	Caudal vertebra	Adult	3.1	11.0	12.0	5.4	33.3	− 17.4	3.2	20.7
1335-110	US 1039	Cattle	Right metatarsal	Sub/adult	–	–	14.1	6.7	38.4	− 19.2	3.2	–
1336-110	US 1041	Cattle	2nd Phalanx	> 2 years	–	–	12.6	6.9	34.5	− 18.4	3.2	–
1337-110	US 1048	Cattle	1st Phalanx	> 2 years	–	–	14.6	6.8	39.3	− 18.8	3.1	–
GUM-32	US 1011	Sheep	Left mandible	4–6 years	2.5	9.0	14.2	6.2	38.2	− 19.3	3.1	5.0
GUM-33	US 1048	Sheep	Left mandible	4–6 years	2.1	7.6	14.0	8.6	37.6	− 18.9	3.1	5.0
GUM-34	US 1053	Sheep	Left mandible	9–12 months	1.8	6.8	12.3	8.5	33.7	− 17.7	3.2	4.1
GUM-35	US 1011	Sheep	Left mandible	9–12 months	1.9	7.5	12.8	7.8	34.7	− 16.9	3.2	4.1
GUM-92	US 1037	Sheep	Left mandible	9–12 months	2.4	8.4	13.2	6.5	36.8	− 17.5	3.2	10.1
GUM-93	US 1033	Sheep	Left mandible	9–12 months	1.7	7.4	10.5	6.9	36.6	− 21.6	*4.1*	8.7
GUM-36	US 1044	Goat	Left mandible	9–12 months	1.9	7.3	12.5	8.0	34.1	− 19.6	3.2	4.6
GUM-25	US 1053	Dog	Right mandible	> 6 months	1.6	7.0	12.3	9.0	32.8	− 19.3	3.1	4.4
GUM-26	US 1053	Dog	Right mandible	> 6 months	0.8	5.8	11.7	10.5	30.9	− 20.6	3.1	7.6
GUM-27	US 1044	Dog	Right mandible	> 6 months	2.9	9.9	13.4	9.4	35.5	− 19.3	3.1	11.0
GUM-28	US 1011	Dog	Right mandible	> 6 months	*0.2*	3.1	5.5	8.5	16.4	− 21.2	3.5	0.7
GUM-29	US 1011	Dog	Right mandible	5–6 months	2.1	8.0	12.0	8.4	32.5	− 19.0	3.2	5.2
GUM-37	US 1044	Pig	Humerus	1.5 years	2.7	9.5	14.0	6.7	37.8	− 20.3	3.2	6.2
GUM-38	US 1011	Pig	Humerus	1.5 years	2.5	9.0	13.8	7.7	37.5	− 20.3	3.2	8.9
GUM-39	US 1058	Pig	Right mandible	8–10 months	*0.4*	3.4	5.2	7.5	15.5	− 21.3	3.2	5.9
GUM-40	US 1041	Pig	Right mandible	8–10 months	2.0	7.9	12.0	6.5	32.4	− 21.0	3.3	9.9
GUM-94	C12	Pig	Right mandible	16–18 months	1.1	5.2	13.3	9.0	36.7	− 18.5	3.2	6.0
GUM-41	US 1044	Wild boar	Left radius	> 1 year	0.6	5.4	6.0	6.1	15.7	− 21.1	3.1	8.2
GUM-42	US 1053	Wild boar	Left radius	> 1 year	2.4	8.5	14.1	7.2	37.7	− 19.8	3.1	12.5
GUM-43	US 1044	*Sus* sp.	Left radius	> 1 year	3.0	10.2	14.6	7.7	39.6	− 21.1	3.2	13.0
GUM-44	US 1044	*Sus* sp.	Left radius	5–6 months	3.1	10.7	13.7	8.5	37.0	− 19.6	3.2	10.2
GUM-17	US 1044	Auroch	Right metacarpal	> 2y	*0.3*	3.7	8.2	8.8	21.8	− 20.1	3.1	*0.9*
GUM-23	US 1036	Roe deer	Left humerus	Adult	2.6	9.5	13.0	7.6	35.7	− 23.2	3.2	14.5
GUM-24	US 1044	Deer	Right metatarsal	Adult	0.9	4.2	10.2	6.5	27.5	− 22.2	3.1	6.6
GUM-30	US 1055	Horse	Right metapodial	Adult	1.1	5.4	7.2	4.5	19.6	− 20.5	3.2	3.7
GUM-31	US 1050	Hare	Left femur	Adult	2.3	8.6	14.0	5.8	38.2	− 22.0	3.2	9.0
GUM-89	US 1037	Fox	Left mandible	5–6 months	2.0	7.7	11.9	9.3	32.7	− 19.2	3.2	6.2
GUM-90	US 1036	Fox	Left femur	Adult	2.6	9.1	14.0	9.1	38.6	− 19.1	3.2	14.8
GUM-22	US 1036	Beaver	Left tibia	Adult	1.9	7.5	12.8	7.4	33.7	− 21.6	3.1	5.5
GUM-45	US1044	Pond turtle	Carapace	Adult	1.2	5.8	*3.5*	8.1	*11.2*	− 23.7	*3.7*	3.4
GUM-46	US1053	Pond turtle	Carapace	Adult	2.3	8.5	8.5	8.9	24.5	− 24.1	3.4	2.8
GUM-98	M1/1961	Pond turtle	Plastron	Adult	1.3	5.9	7.9	7.9	21.8	− 23.3	3.2	6.6
GUM-47	US1053	Carp	Opercle	Adult	0.5	4.3	*3.0*	7.5	*9.1*	− 24.2	3.6	11.2
GUM-48	US1017	Carp	Preopercle	Adult	*0.2*	3.0	8.5	5.1	25.2	− 26.0	3.4	6.6
GUM-49	US1053	Catfish	Vertebrae	Adult	0.5	3.9	9.0	11.2	26.3	− 20.4	3.4	5.2
GUM-50	US1053	Catfish	Vertebrae	Adult	*0.2*	3.8	*3.6*	11.4	*9.9*	− 19.7	3.2	2.8
GUM-51	US1053	Catfish	Vertebrae	Adult	0.7	4.6	5.3	10.8	14.9	− 21.0	3.3	2.3
GUM-52	US1044	Catfish	Radius	Adult	1.2	5.4	*2.3*	9.5	*6.3*	− 21.8	3.2	3.6
GUM-53	US1019	Catfish	Vertebrae	Adult	1.1	5.7	6.8	9.2	18.9	− 24.1	3.2	2.4
GUM-99	M1/1961	Catfish	Pectoral-fin spine	Adult	1.7	7.2	12.0	9.7	34.8	− 20.5	3.4	10.0

In italics the results not meeting the quality criteria.

The tell settlement from where animal samples originate comprises a wider range of radiocarbon dates than the cemetery (Table S8), so our isotopic results can reflect variations in human-animal relationship during a larger time interval.

Bones had a standardised pre-treatment for collagen extraction^80^. An aliquot of bone powder of 5 mg was collected for the sample’s elemental analysis (%C and %N). This analysis allows for assessing the degree of preservation of bone collagen^81^.

An approximate amount of 250–350 mg of bone powder was employed for collagen purification following Bocherens et al.^82^ with some in-house improvements. First, the bone powder was demineralized by digestion in 1 M HCl during 20 min. Unwanted organic components were then solubilized by incubation in NaOH 0.125 M during 20 h, at room temperature. Each digestion was followed by a filtration (5μ) to eliminate carbonates, organic contaminants, small fragments of collagen and other organic molecules of low molecular weight. The collagen thus obtained was diluted in mild HCl solution (0.01 M) for 17 h at 100 °C and filtered. The gelatine obtained was freeze-dried to be analysed by IRMS. However, due to the poor results using this method in fish collagen, we tested other variations (see section “Results” of Supplementary Material S1), and, in the end, we employed 500 mg of bone powder and eliminated the NaOH step to extract the collagen.

The bone collagen of RoAMS-1335-110, RoAMS-1336-110 and RoAMS-1337-110 (all cattle remains) were extracted separately^10^ following Sava et al.^83^. These samples were analysed directly by IRMS.

### Mollusc conchiolin

A total of 17 shell-conchiolin were extracted (3 *U. crassus*, 4 *U. pictorum*, 5 *U. tumidus* and 5 *Anodonta* sp.), which represent the local freshwater molluscs documented in the site (Fig. S2). The marine shells discovered at Gumelnița (e.g., *Spondylus* sp., *Cardium* sp., etc.) are personal ornaments, not remains from cooking or eating.

The conchiolin was obtained by decalcification of the shells following García-Vázquez et al.^84^ First, the shells were pre-washed with distilled water to remove any remaining soil contaminant. Then the shells were placed in 100 ml beakers. Large shells were broken into fragments. They were immersed in 1 M HCl (except GUM-77 to GUM-80 which were part of a test with 2.5 HCl) till the reaction stopped (CaCO_3_ + 2HCl → CaCl_2_ + H_2_O + CO_2_). We collected the HCl and placed it in 15 ml centrifuge tubes to remove the supernatant and keep the pellet, which will be the conchiolin. We repeated this process by adding again HCl to the shells to continue de digestion till the reaction stopped and centrifuging the HCl until the shell carbonate was wholly digested. The resultant pellet was then washed with distilled water and centrifuged 3 times. Finally, the pellet was freeze-dried and analysed by IRMS.

To calculate the fractionation between the proteins of the body of the animal and the conchiolin of the shell, we used the offset^84^: Δ^15^_defatted body-conchiolin_ =  + 0.95‰, Δ^13^C _defatted body-conchiolin_ =  + 0.93‰.

### Isotopic measurements

Sample preparation for isotopic analysis was performed at the Stable Isotopes Laboratory of the ArchaeoScience Platform of the University of Bucharest and at the Molecular Palaeontology Lab of the University Institute of Geology (IUX) of the University of A Coruña.

The IRMS analyses were carried out in the Unit of Instrumental Techniques of Analysis (UTIA) of the UDC Research Support Services (SAI). For the stable isotope analysis, approximately 0.5 mg of lyophilised collagen/conchiolin and 0.1 mg (for the smaller seeds) to 5 mg for charred seeds, were loaded into tin capsules and analysed directly on a DeltaV Advantage (ThermoScientific) coupled to a Flash IRMS EA IsoLink CNS (Thermo Scientific) Elemental Analyser Fash IRMS EA Isolink CNS (Thermo Scientific), via a Conflo IV Interface. An analytical precision (reproducibility) was better than 0.2‰ for both carbon and nitrogen. Stable carbon and nitrogen isotope compositions were calibrated relative to Vienna PeeDee Belemnite (VPDB) and atmospheric air (AIR). The following secondary standards were employed: USGS 40 (− 4.52‰), USGS41a (+ 47.55‰), IAEA-N-1 (+ 0.4‰), IAEA-N-2 (+ 20.3‰) and USGS-25 (− 30.4‰) for δ^15^N, and USGS 40 (− 26.39‰), USGS41a (+ 36.55‰) NBS 22 (− 30.031‰), IAEA-CH-6 (− 10.449‰) and USGS 24 (− 16.049‰) for δ^13^C. To test for precision acetanilide was employed as an internal standard. Reproducibility resulting in ± 0.15‰ (n = 10). The results are reported using the delta (δ) notation and are expressed in parts per thousand (‰).

### Statistics

Statistic calculations and plots were made using RStudio 2022.07.0 + 548^85^ and Past 4.05^86^. Plots were edited with Adobe Illustrator CS3.

### Radiocarbon dating and freshwater reservoir

A total of 29 ^14^C dates are presented here. Two of them are new and were measured in the labs of CEDAD, University of Lecce (LTL17515A) and Poznań Radiocarbon Laboratory (Poz-52559). The rest were already published^10,11,87^ (Table S8).

To reconstruct the diet of the population of Gumelnița, we employed the Bayesian mixing model software FRUITS (Food Reconstruction Using Isotopic Transferred Signals)^56^ (for settings information see Sect. 5.1 of the Supplementary Material S1). The freshwater reservoir effect (FRE) of the Danube Gorges region for a 100% freshwater diet was stabilised at 545 ± 70 ^14^C years^88^. To correct the human radiocarbon dates for FRE, the percentage of freshwater food in the diet obtained by FRUITS was used to obtain the FRE correction following Sayle et al.^89^. Radiocarbon dates were calibrated using OxCal 4.4^90^ and the calibration curve from Reimer et al.^91^ applying the FRE correction only for adult humans.

## Results

### Charred seeds and grains

The isotopic results of the analysed plants are in Table 2. All the samples were measured in duplicated except GUM-56, GUM-62, GUM-63, GUM-65, GUM-66, GUM-67, GUM-68, GUM-69, GUM-70, GUM-75 and GUM-76. In the case of GUM-73-74, two seeds where measured together.

δ^15^N values range from 1.3‰ for the pulse bitter vetch, to 13.4‰ for a grain of barley, with a mean of 6.2‰ (± 2.62). δ^13^C values range from − 28.7‰ for a grain of emmer wheat to − 22.4‰ for another grain of the same species, with a mean of − 24.8‰ (± 1.62). For the domestic cereals, the mean of δ^15^N values is 6.1‰ (± 2.67) and ranges from 3.7 to 13.4‰ and the mean of δ^13^C values is − 24.8‰ (± 1.72) with a range of − 28.7 to − 22.4‰. The pulses from Gumelnița period have a mean of 7.4‰ (± 0.58) with a range of 6.9–8.2‰ for δ^15^N values, and − 23.2‰ (± 0.25) with a range of − 23.4 to − 22.8‰ for the δ^13^C values.

On average, pulses (14.1) (n = 4) have a higher atomic C:N ratio than the cereals (18.7) (n = 12) due to a higher content of protein, but the range of values overlap (pulses range: 10.6–18.1; cereals range: 11.8–25.1).

### Collagen

The isotopic and elemental results obtained from collagen extracted from humans and animals are presented in Tables 3 and 4, respectively. All the samples were measured in duplicated except GUM-4, GUM-7, GUM-17 and GUM-28 which didn’t produce enough collagen for replicates. Elemental analysis in bone powder show %N mean values of 1.4% (± 0.84) with a range of 0.2–3.1. Nitrogen content lower than 0.5% is indicative of poor collagen preservation. Collagen samples with %C higher than 13%, and %N higher than 5%^92^; with a C:N atomic ratio between 2.9 and 3.6^93^ (where 3.2 is the theoretical value of the collagen^94^), and an extraction yield higher than 1.5%^95^ are likely to preserve the original carbon and nitrogen isotopic values.

The majority of human samples meet the quality criteria. Five samples have the %N in bone powder under 0.5% but over 0.3% (GUM-4, GUM-7, GUM-8, GUM-9, and GUM-15). However, the rest of the quality criteria are met. In the case of GUM-4 and GUM-7, only one duplicate measurement could be done, which indicates that the sample had little organic matter preserved, but at the same time the quality of the collagen is acceptable. On average, the δ^15^N of all adult humans is 10.2‰ (± 0.62) with a range of 9.0–11.5‰, and δ^13^C is − 20.3‰ (± 0.31) with values ranging from − 21.0 to − 19.6‰. The skeletons identified as females have a mean of 9.8‰ in the δ^15^N (± 0.45; range: 9.0–10.5‰) and of − 20.3‰ in the δ^13^C (± 0.31; range: − 21.0 to − 19.8‰). In the case of the skeletons identified as males, they have a mean of δ^15^N of 10.3‰ (± 0.52; range: 9.5–11.5‰) and of − 20.4‰ (± 0.24; range: − 20.8 to − 20.0‰) for the δ^13^C. On average, females have slightly lower δ^15^N values and slightly higher δ^13^C values than males.

The animal bones with a %N lower than 0.5 in bone powder are GUM-17, GUM-28, GUM-39, GUM-48 and GUM-50. GUM-17 has a yield of 0.9, and GUM-28 of 0.7, so we are going to reject these data based on that. GUM-45, GUM-47, GUM-50 and GUM-52 have the %N in collagen under 5%, and also the %C under 13%. Of these samples, GUM-45 also has a C:N ratio outside the acceptable range. GUM-93 has a C:N ratio greater than 3.6, therefore we did not include these 5 data points.

Domestic mammals have a mean of δ^15^N values of 7.6‰ (± 1.15; range 5.4–10.5‰) and δ^13^C values of –19.1‰ (± 1.11; − 21.3 to − 16.9‰), but within this group, there are animals with herbivore and omnivore diets. Domestic herbivores have δ^15^N values mean of 7.2‰ (± 0.85; range 5.4–8.6‰) and a δ^13^C values mean of − 18.5‰ (± 0.84; range: − 19.7 to − 16.9‰). Of these, cattle show a lower δ^15^N values mean (6.9‰ ± 0.67; range: 5.4–7.6‰) and a lower δ^13^C values mean (− 18.7‰ ± 0.66; range: − 19.7 to − 17.4‰) than the sheep δ^15^N values (7.5‰ ± 0.99; range: 6.2–8.6‰) and δ^13^C values mean (− 18.0‰ ± 0.90; range: − 19.3 to − 16.9‰). Wild terrestrial ungulates have a δ^15^N values mean of 6.3‰ (± 1.14; range: 4.5–7.6‰) and δ^13^C of − 21.9‰ (± 0.88; range: − 23.2 to − 20.5‰).

Of the domestic omnivores, dogs have higher δ^15^N values (9.3‰ ± 0.75; range: 8.4–10.5‰) and lower δ^13^C values mean (− 19.6‰ ± 0.63; range: − 20.6 to − 19.0‰) than pigs δ^15^N values (7.5‰ ± 0.89; range: 6.5–9.0‰) and δ^13^C values means (− 20.3‰ ± 0.98; range: − 21.3 to − 18.5‰). These dogs show a diet with a trophic level closer to the humans than the pigs. The δ^15^N values mean of all suids is 7.4‰ (± 0.87; range: 6.1–9.0‰) and the δ^13^C values mean − 20.3‰ (± 0.86; range: − 21.3 to − 18.5‰). Wild boars have a lower δ^15^N values mean (6.7‰ ± 0.53; range 6.1–7.2) and a lower δ^13^C values mean (− 20.5‰ ± 0.64; range − 21.1 to − 19.8‰) than the pigs. Even though the values are similar, data indicate that pigs have a slightly more animal protein in their diet than the wild boars, which supports the anatomical identification of swine remains. According to these results, pigs' diet could be determined by human management and pigs (but dogs also) used to be fed with human leftovers. In the case of the bones identified and *Sus* sp. it is not easy to attribute whether they belong to the domestic or the wild species, but their higher δ^15^N values than the wild boars, suggest they are closer to domestic pigs.

Freshwater-feeding vertebrates (turtles and fishes) have a δ^15^N values mean of 8.5‰ (± 2.20; range: 5.1–11.2‰) and a δ^13^C values mean of − 23.2‰ (± 2.15; range: − 26.0 to − 20.4‰). Of these animals, the European carp have the lower δ^15^N values, as this species is omnivorous, and the catfish the higher values in the δ^15^N values (10.2‰ ± 0.80; range: 9.2–11.2‰) and δ^13^C values mean (− 21.5‰ ± 1.51; range: − 24.1 to − 20.4‰). The catfish is the top predator of the Danube and it can also consume terrestrial animals, as suggested by the higher δ^13^C values. Pond turtles are in an intermediate position (δ^15^N: 8.4‰ ± 0.55; range: 7.9–8.9‰) (δ^13^C: − 23.7‰ ± 0.39; range: − 24.1 to − 23.3‰) and are also omnivores.

### Conchiolin

Conchiolin results are in Table 5. Data is from García-Vázquez et al.^84^. Raw values of the conchiolin are transformed into the defatted body because there is an offset between those tissues. The average mean of the values corrected to the defatted body value is 8.5‰ (± 0.74) with a range of 7.2–9.8‰ for the δ^15^N, and − 25.0‰ (± 2.09) with a range of − 29.0 to − 21.1‰ for the δ^13^C.

**Table 5:** Conchiolin isotopic results from García-Vázquez et al.^84^ ).

Lab ID	Feature	Specie	% N	δ^15^N (‰)	%C	δ^13^C (‰)	C/N	δ^15^N (‰) (corrected)	δ^13^C (‰) (corrected)
GUM-77	US1044	*U. tumidus*	1.5	8.2	4.9	− 22.1	3.9	9.2	− 21.1
GUM-78	US1044	*U. crassus*	3.1	8.8	9.6	− 26.5	3.6	9.8	− 25.6
GUM-79	US1036	*U. pictorum*	1.2	6.7	5.2	− 26.9	*4.9*	–	–
GUM-80	US1036	*Anodonta* sp.	7.7	8.0	22.1	− 26.6	3.3	9.0	− 25.7
GUM-103	US1050	*U. tumidus*	2.2	6.4	7.1	− 25.2	3.8	7.4	− 24.2
GUM-104	US1044	*U. tumidus*	11.8	7.1	34.9	− 25.2	3.4	8.0	− 24.2
GUM-105	US1053	*U. tumidus*	12.5	7.9	36.6	− 23.9	3.4	8.8	− 23.0
GUM-106	US1053	*U. tumidus*	15.0	7.4	45.2	− 29.7	3.5	8.3	− 28.7
GUM-107	US1053	*U. crassus*	11.9	7.9	34.9	− 26.7	3.4	8.9	− 25.8
GUM-108	US1053	*U. crassus*	14.9	6.9	44.7	− 26.2	3.5	7.9	− 25.2
GUM-109	US1053	*U. pictorum*	14.2	6.3	42.1	− 29.9	3.4	7.2	− 29.0
GUM-110	US1053	*U. pictorum*	12.4	8.3	36.7	− 26.8	3.5	9.2	− 25.8
GUM-111	US1053	*U. pictorum*	13.7	7.6	40.3	− 23.9	3.4	8.6	− 23.0
GUM-112	US1036	*Anodonta* sp.	4.8	6.9	17.6	− 26.6	*4.3*	7.8	− 25.6
GUM-113	US1053	*Anodonta* sp.	2.1	–	17.7	− 28.3	*10.0*	–	–
GUM-114	US1044	*Anodonta* sp.	9.1	6.8	34.4	− 26.4	*4.4*	–	–
GUM-115	US1050	*Anodonta* sp.	1.4	–	7.1	− 25.7	*5.8*	–	–
GUM-116	US1050	*Anodonta* sp.	6.0	6.9	19.5	− 25.2	3.8	7.8	− 24.3

δ^15^N and δ^13^C raw values are corrected to body values (defatted). In italics the C:N values that are outside the accepted range.

### Data modelling

For the FRUITS Bayesian model, we grouped our stable isotope results in terrestrial mammals (domestic and wild), domestic plants (cereals and pulses), fish and shellfish. The results are represented in Fig. 5 (see Table S7 for the numeric values, and figures from S7–S32 for plots of individual results). On average, plant protein represents half of the diet (52%) for this population, followed by freshwater resources (26%) and terrestrial mammals (22%).

The results on the calibration of the radiocarbon dates for the FRE on adult human bones are in Table S8 and are represented in Fig. 7. Due to FRUITS errors transferred to the FRE range, the obtained intervals are wider than the original calibrated dates. Therefore, the difference between calibrated dates with and without FRE is just 147 years.

## Discussion

### Crop management and the environment of the Gumelnița site

**Figure 2:**
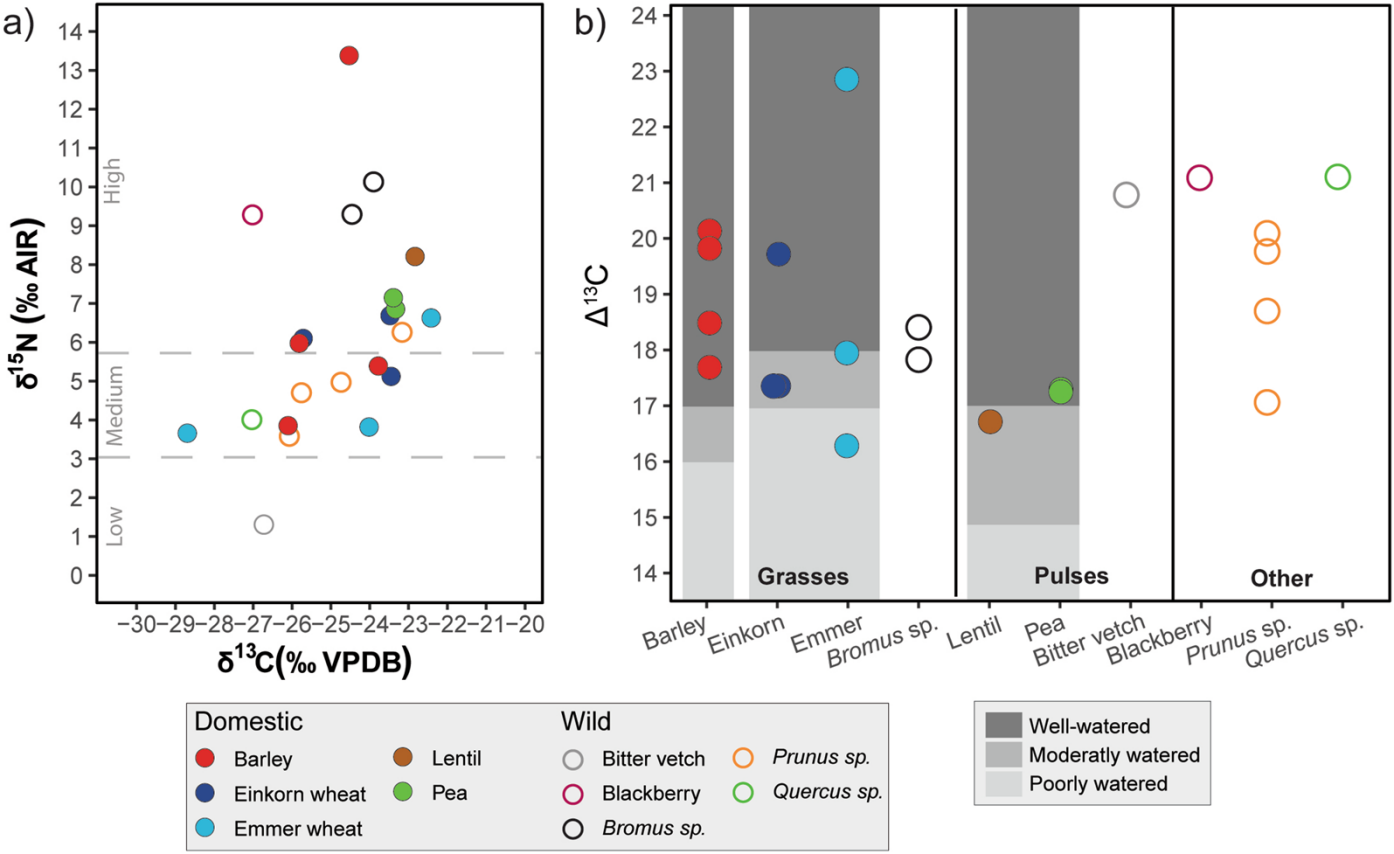
(**a**) Bivariate plot of the stable isotopes results of δ^15^N and δ^13^C of the seeds analysed. Low δ^15^N values indicates no manure, medium 10–15 tons/ha and high more than 35 tons/ha^75^. (**b**) Δ^13^C of the analysed seeds. In different shades of grey, the water availability is indicated^76^.

The isotopic values of the plants studied in this paper are represented in a bivariate plot in Fig. 2a. Most domestic plants group in the same area except for two outliers: one in the δ^15^N (GUM-67), and the other in the δ^13^C (GUM-54). These two outliers could represent the local conditions of the soil where the plants have grown.

Domestic plants from Gumelnița show medium to high addition of manure, based on Fraser et al.^75^ (Fig. 2a), especially the pulses. Interestingly, the difference between the domestic pulses (lentil and pea) and the bitter vetch, which has a typical value of a nitrogen-fixing plant^96^, could indicate that this plant was wild, or if it was cultivated, wasn’t manured. However, N-fixation is inhibited by the presence of inorganic N in the soil^97^. Thus it may not cause any distinctive signature under manured soils or in alluvial plains^96^.

Crops are subject to human management, and unlike wild plants, they will mainly reflect human intervention, rather than natural factors. Thus, in each harvest, the soil nutrients assimilated by the plants are removed in the form of biomass. The soils then would be depleted of elements such as nitrogen, so it is necessary to add fertilizer in order to continue harvesting new crops frequently. To avoid this, crop rotation was developed. Nitrogen-fixing plants fix nitrogen from the air, thereby contributing to fertilizing the soil, so it is typical in crop rotation to plant pulses and don’t add any fertilizer that year, but our results don’t support crop rotation with pulses because these plants also have been fertilized.

It would be expected that wild plants would present lower values of δ^15^N than manured domestic plants, but in our case, except the bitter vetch, all of our charred seed and grains, have δ^15^N values over 3‰. Due to the tell settlement location in the Danube floodplain, this area possibly had riparian vegetation with high δ^15^N due to the annual fertilization by the river. The higher values of the blackberry and the *Bromus* sp. seem to support this. On the terrace and, in general, outside the floodplain, the landscape could be composed of open areas and closed forests. Due to the canopy effect, the oak acorn seems to indicate that it would come from a more closed environment than the *Prunus* sp. fruits, which present different δ^13^C values and perhaps belong to different degrees of vegetation coverage. This is also reflected in the isotopic values of wild ungulates.

There's a possibility that cereal crops could be sown in autumn/winter in the floodplain, and harvested in late spring before the Danube floods its banks. These crops should show high δ^15^N values like GUM-67, but more data would be needed to support this hypothesis. Nor can it be ruled out that the high δ^15^N values of the legumes come from cultivation in the floodplain.

Our cereal values can be comparable with Neolithic ones from Bulgaria^98^, where authors also found medium to high contribution of manure in their crops, but the pulses had a low amount of it.

The domestic cereals and pulses from Gumelnița show watering from moderate to well-watered (Fig. 2b), and for GUM-54, this watering was very high. In the case of wild plants, although there are no comparison values established, it does not appear that they lacked water during their growth. The existence of a palaeochannel next to the tell indicates that it could perhaps have been used for irrigation as well as to bring drinking water closer to the settlement. On the assumption that the palaeochannel was used to irrigate the crops, this area closest to the terrace could have been isolated from the Danube flood and it could be where the cultivated fields were located.

### Livestock and wild animal resources

**Figure 3:**
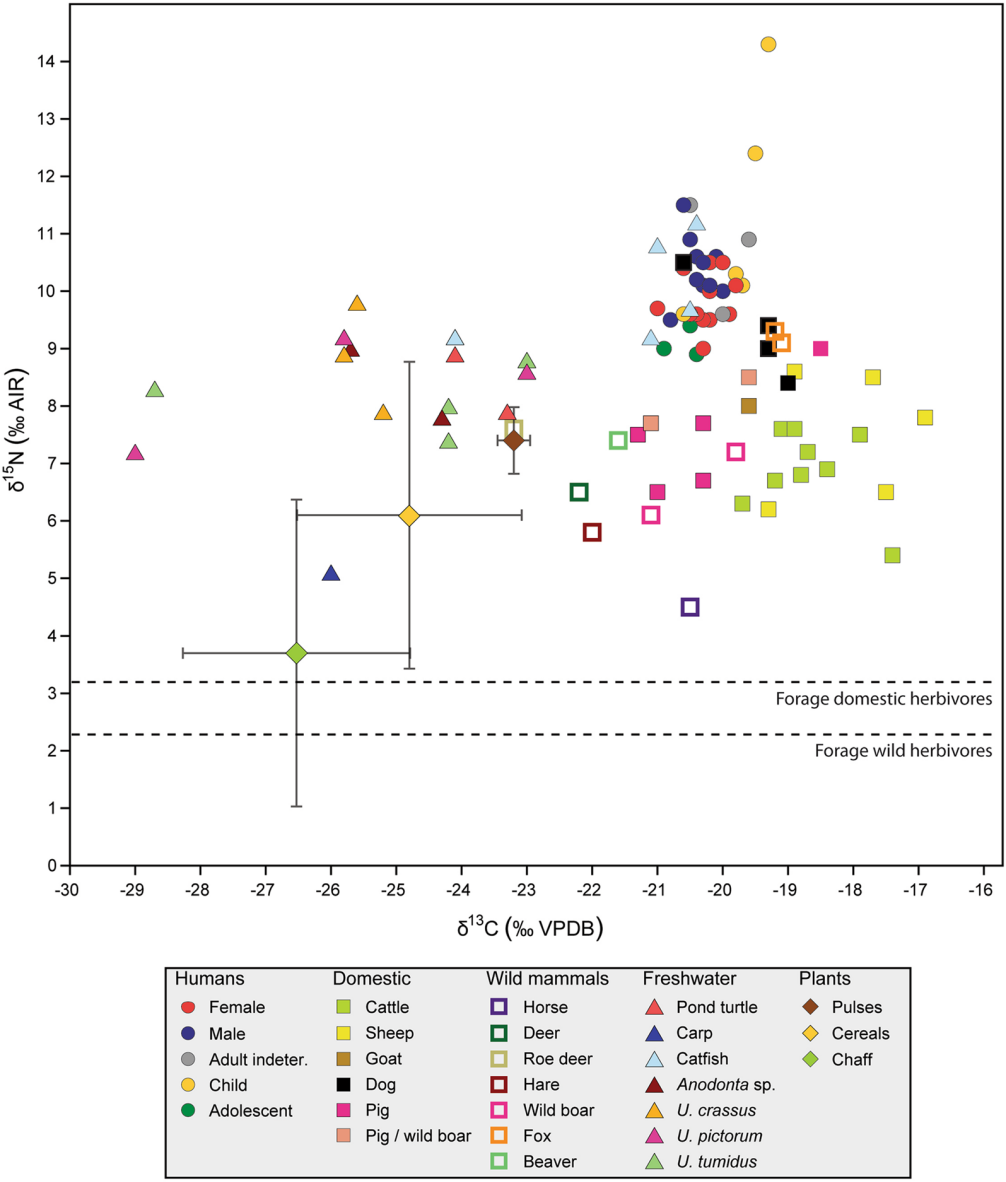
Isotopic data of Gumelnița site. Cereals, pulses and chaff are represented by the media and the standard deviation. The δ^15^N of the forage of wild and domestic herbivores is represented by a dashed line.

The isotopic data of Gumelnița samples are represented in a bivariate plot in Fig. 3. Livestock animals have more positive δ^13^C values than wild mammals. The δ^13^C values of the roe deer and the red deer denotes that they come from a more closed environment than the horse, but even this animal has more negative values than sheep and cattle.

The δ^15^N of the forage is calculated by subtracting 4‰ (as an average of the 3–5‰ range associated with the trophic shift)^98^. The forage of domestic herbivores is higher (3.2‰) than the wild ones (2.3‰). This may be because they are feeding on cultivated plants such as cereal chaff. In our data, the chaff means calculated from cereal grains (3.7‰) is very similar to the estimated forage. Chaff δ^13^C mean is − 26.6‰. The cereal grain with less watering (GUM-55) has a chaff value of − 24.3 ‰. The fractionation plant–herbivore is ~ 5‰^99^, so a herbivore with a 100% diet of this kind of grains, would have a collagen based δ^13^C value of − 19.3‰. Offset values have not been established for lentil and peas parts, but a small offset in δ^15^N values of manured *Vicia faba* between different plant components was found^100^. The mean of Gumelnița δ^13^C values in pulses is − 23.2‰, which would give a collagen value for a diet of 100% legumes of − 18.2‰. Feeding with chaff and other crop leftovers can explain part of the δ^13^C values of cattle and sheep, although a small percentage of C4 plants in the diet is needed to explain all the values.

In Romania, only about 2% of the species are C4^101^. Roe-deer values from the Cheia site (Hamangia “culture”, first half of the 5th millennium BC, Dobrudja area)^69^ show a wide variety of ecosystem provenience, and some of them are similar to those of the sheep and cattle values that go towards C4 plants both in Cheia and in our data. In that paper, the authors propose that sheep from Cheia went to feed in the salt marshes on the western coast of the Black Sea where plants of the family *Chenopodiaceae* are widespread. Analogously to this, the roe deer from Cheia with the most positive values would come from these areas. To test if Gumelnița sheep and cattle with these higher δ^13^C values fed on the Black Sea coast (a hypothesis that we must consider at least at a theoretical level), a mobility study has to be done because the Gumelnița site is not close to the coast (more than 200 km).

Another possibility with wild C4 plants is the *Eragrostio-Polygonion arenastri* communities of trampled soil that nowadays exist in the eastern part of the Danube in Romania, characterised by the C4-assimilation syndrome^102^. *Echinochloa crus-galli*, or cockspur grass, was found in Gumelnița site (unpublished) and belong to this C4 plants community. This plant is a type of millet native to Eurasia and was domesticated in Japan 4500 year BP ago^103^. Nowadays can be grown for grain or forage, but also it is mainly found in wet and degraded environments like swamps, ponds or temporarily flooded palustrine wetlands, and seasonally wet habitats^104^. Therefore, we propose that cockspur grass could have served as food for sheep and cattle that grazed in the vicinity of the site in the floodplain, explaining higher δ^13^C values of these animals respecting wild herbivores.

Bone fragments from in the tell, show household waste characteristics like traces of cutting, disarticulation, defleshing, burning and traces of carnivorous teeth/swine, etc.^11^. Dogs’ isotopic values are close to humans, especially GUM-26, which overlaps human distribution. The other 3 data from dogs have a lower δ^15^N and higher δ^13^C values and are in the same area (Fig. 3) as the foxes and some of the pigs. This could indicate that those three species could be feeding on human waste, confirming the relation with the teeth marks in the bones. Interestingly, this same isotopic relative position with humans has been found in dogs and foxes of the Bronze Age of the northeastern of the Iberian Peninsula^105,106^. The earliest synanthropic commensal behaviour of foxes was detected in the Aurignacian, around 42,000 years ago^107^. This behaviour could explain why dogs and foxes of Gumelnița site have practically the same isotopic values. Hare is one of the potential natural prey of foxes but the δ^13^C value of the hare from Gumelnița is more than one trophic level apart, which indicates that the fox diet possibly included cultivated plants (cereal grains, pulses), the remains of domestic animals and/or micromammals that fed on crops.

Besides dogs and foxes eating human waste, they were also part of the human diet occasionally or sacrificed for their skin. Cutting marks in dog bones have been found in Gumelnița^10^ and other KGK VI sites^63,64,108,109^. Of the two foxes analysed, one was an adult and the other a subadut, and all the dogs older than 6 months, except GUM-29 which was 5–6 months old. This utilisation of the animal bodies could be part of the artificial selection of the domestication process: the dogs that weren’t good enough for work (hunting, herding, etc.) or showed unwanted behaviour could have had this end. This artificial selection process could also control the dog population.

In the case of the suids, they show values corresponding to a gradual increase of animal protein in their diet (tendency to higher δ^15^N and δ^13^C). GUM-94 includes the highest amount of terrestrial animal protein in the diet and GUM-41, the lowest, being the difference in δ^15^N of 2.9‰, almost one trophic level apart. The ecological niche of the wild boar was defined^72^ for southeast Romania during the Chalcolithic by carbon isotope values always higher than − 22‰ and in most cases higher than − 21‰, which places the wild boar as a species of open environments. In the Gumelnița site the wild boar values are − 21.1‰ and − 19.8‰. Also an enrichment of 1‰ in the wild boar δ^15^N values (compared with other wild herbivores) was observed^72^, confirming a more omnivorous diet. But in our case the δ^15^N is within the variation of wild herbivores, even though the two wild boars analysed are among the suids with lower δ^15^N of the sampling, suggesting less manured vegetables in the diet. Wild boar diet nowadays is dominated by plants (~ 90%)^110^, similar to the case of Chalcolithic wild boars from Romania. In the case of the pigs, with different amount of animal protein in the diet, higher δ^15^N values could be more related to the consumption of domestic animals (and dairy products) and domestic cereals rather than to the freshwater resources or game. Regarding these results, it is not easy to separate wild from domestic pigs, considering their frequent hybridization.

From the freshwater environments, we analysed fishes, molluscs, turtles and a beaver. Of those, the beaver has a semiaquatic life and our value indicates that it fed on C3 plants. The pond turtles are omnivores, whose values are close to one of the catfish and some of the molluscs. Freshwater mussels are omnivores that feed on animal and vegetal particles, living or partially decomposed, present in suspension and on phytobenthic species^111^. Our data show higher variation in δ^13^C values due to the different origins of the shells in the sense of habitat, not area. So, the freshwater mussels with less negative δ^13^C values could be a result of feeding near the river bank or lakeside where there was an influence of terrestrial carbon input^112^. The rest of the δ^13^C values variation could be explained by the different eutrophication degrees of the different water bodies, and the most negative values could be explained by the presence of methanogenic bacteria^113^. Particulate organic matter of the Danube River nowadays has a value of δ^15^N of 4.88‰^114^, which is within the trophic level offset of the mean of the freshwater mussels (8.5‰) and their diet.

Regarding the fish, catfish can feed on freshwater fish, molluscs, insects, worms, etc., but also terrestrial animals, birds, and can even predate on other catfish. Our results put them as top predators. All the catfish analysed except GUM-53 show a δ^13^C value in the middle of freshwater and terrestrial isotopic area, which is consistent with the diet observed in present-day animals. GUM-53 have values that put this individual on a diet based on freshwater animals, in particular, the carp GUM-47 has isotopic values compatible with being the prey type of this catfish. Both GUM-53 and GUM-57 have lower δ^15^N and δ^13^C values compared to the other catfish (GUM-53) and to molluscs in the case of GUM-57, which could suggest a different habitat than the other fish.

### Human diet

**Figure 4:**
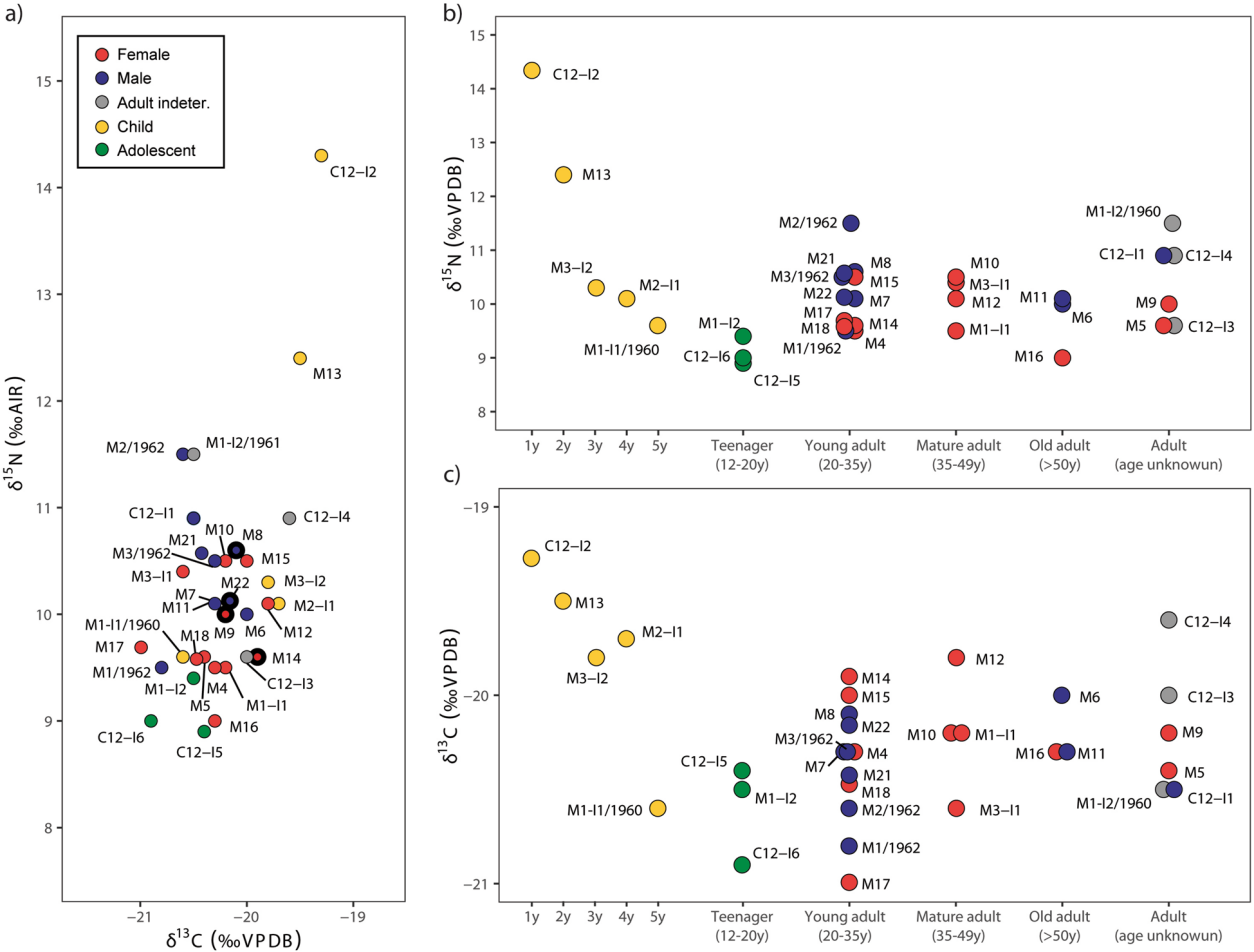
(**a**) Bivariate plot of δ^13^C and δ^15^N of the humans from Gumelnița. Individuals with bold line have flint in their graves. (**b**) Bivariate plot of age in years vs. δ^15^N. (**c**) Bivariate plot of age in years vs.δ^13^C.

The isotopic values of Gumelnița humans are concentrated, as can be seen in Fig. 3. In Fig. 4a, only human results are represented. The most divergent data are the children. In Fig. 4b, we can observe the differences of the δ^15^N values in different stages of life. C12-I2 is a 1-year-old baby with the highest δ^15^N and δ^13^C values of all the samples. M13, a child between 1 and 3 years old, show a lower δ^15^N value than C12-I2, and this decrease continues with the rest of the children till the adolescents, which show the lowest δ^15^N of the non-adults. After this, the values are more homogeneous in the adults. This effect of increasing δ^15^N values with breastfeeding and decreasing with active growth have been described also in other mammals^80,115^. During the first months of life, infants have a diet of 100% mothers’ milk, which makes them have a higher trophic level than their mothers. This offset was observed to be about 2–3‰ in humans^116,117^. From our data, C12-I2 has breastfeeding values, and M13 starts showing a mixture diet of breastmilk and other foods with lower δ^15^N values. Studies on modern populations suggest that after 6 months of age, the incorporation of more complex foods is required^118^, which marks the beginning of the weaning process. Nitrogen isotopes are rather insensitive proxies for detecting the start of the weaning process due to bone collagen turnover rates^119^, so this is why C12-I2 still show that high δ^15^N value. C12-I2 has an important difference in δ^15^N values compared with the females. Females have a mean of 9.8‰ and C12-I2 has a value of 14.3‰. The offset is 4.5‰, between the expected trophic level offset (3–5‰)^98^, but towards the upper limit. Changes in food preference and food consumption during pregnancy^120^, and the increase of protein requirements during pregnancy and lactation^121^, make possible that during this period the δ^15^N values of the female body's blood or milk would be higher, so this effect could be diluted in the δ^15^N values of the female bones because it reflects the media during the last years of life.

Many typical weaning foods are high in carbohydrates and low in protein^122,123^, which would produce that difference between C12-I2 and M13. The following children in age group are M3-I2 and M2-I2 of 3 and 4 years, respectively. Again the δ^15^N values decrease with age but is slower this time. The δ^13^C values of these children are less negative than the rest of the adults (except C12-I4 and M-12 which show the same value as M3-I2). This can be explained with a diet rich in cereals, vegetables, domestic animal milk and terrestrial meat, and with none or very few foods of freshwater origin. After this, the 4–6 years old child (M1-I1/1960) and the adolescents show similar values with lower δ^15^N and δ^13^C as a result of growth physiology and maybe a diet with less protein but now with the inclusion of freshwater resources. M1-I1/1960 show pathologies (criba orbitalia and criba cranii) (Table 1) that could indicate dietary deficiencies or metabolic disorders. Weaning and early dietary transitions through childhood affected the juvenile’s nutritional regime^124^. Criba orbitalia have been connected to low iron levels due to anemia^125^ or vitamin (B-12, C) deficiency due to unsanitary living conditions correlated with parasitic infections^126^. Regarding stable isotopes, opposite results have been found in δ^15^N values^124,125^, but these data may indicate that these individuals with criba orbitalia had different diets than the rest. In our case, the difference between M1-I1/1960 and M2-I, could be due to this different diet and related with the pathologies behind criba orbitalia. Human remains are not uncommon intramural^21,127^, mainly of children^127^, some of them showing pathologies^21^, like M1-I1/1960.

On average, the δ^15^N values of Gumelnița males are slightly higher than in females, but this difference has to be tested statistically. Mann–Whitney test for equal medians of the adults with confirmed sex, in the case of the δ^15^N, resulted in p = 0.024, which is lower than 0.05, so the null hypothesis of identical distributions for females and males is rejected. For δ^13^C, was p = 0.445, so for this isotope, they are not significantly different. Doing a hierarchical clustering analysis (Fig. S6) is possible to observe that between the adults there are two main groups (cluster 2.2.1 and cluster 2.2.2) based on δ^15^N. Cluster 2.2.1 is where most of the men are (and also some of the females and weaned children), and 2.2.2 is formed mainly by females and teenagers, and only one adult male is included.

The dietary intake of adult males is 56 g/day, while in females is 46^121^. This suggests a higher preference for proteins in males. But during pregnancy and lactation, these requirements increase to 71 g/day^121^. According to this, it is expected that men could have a higher amount of animal protein in their diet than women. However, it also is expected that women during pregnancy and lactation also had a similar pattern, being nothing of this relating to some social rank or economic status, just human physiology. These differences in preferences towards food with more protein have been observed in a modern population of hunter-gatherers^128^.

There are 4 graves with flint as grave goods (M8, M9, M14 and M22). All of these skeletons belong to young people (except M9, an adult of unknown age) and are two females and two males. Interestingly their δ^13^C values are in the middle of the distribution, and these values could correspond with more game resources, indicating that they could be hunters, and this type of work could be carried out by young people.

Only 3 adults are classified as older than 50 years old. This group show a slight decrease in δ^15^N values compared to the younger groups, but the two males (M11, M6) have higher δ^15^N values than the female (M16).

**Figure 5:**
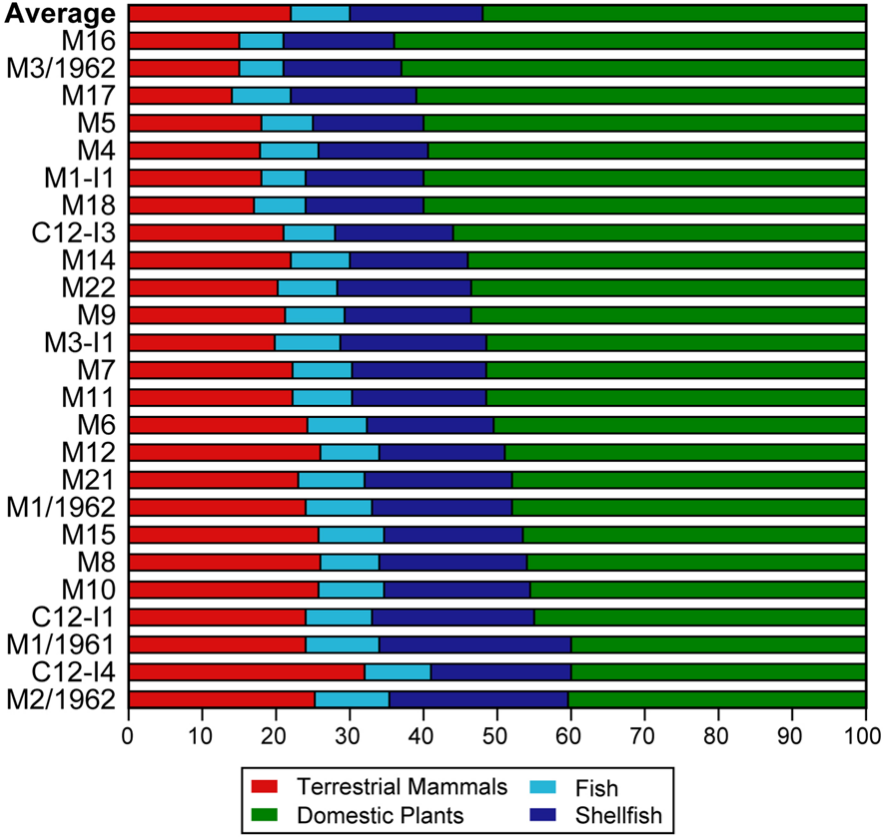
Percentages of the different food groups estimated by FRUITS.

The individual with a higher amount of plants in her diet is M16 (Fig. 5), and also has the lowest δ^15^N among the adults. This woman had a premortem loss of the molars (Fig. S21c), so her diet was under this, and would probably have been dominated by soft foods such as plants and fish. The FRUITS model for this person (Fig. S21) demonstrates this, as the percentage of terrestrial animals in her diet is lower than in the rest of the individuals. M1-I2/1960 (Fig. S29) and M2/1962 (Fig. S31) are among the individuals with a smaller percentage of plants and the adults with higher δ^15^N, but the model attributes it to freshwater resources, mainly shellfish. C12-I4 also has one of the lower percentages of plants, and the model places this human with the highest percentage of terrestrial mammals.

Looking at the human, game, and domestic δ^13^C values, it seems more likely that much of the protein assimilated from terrestrial animals came from the game rather than domestic animals. If the Gumelnița settlement was a farming society, the exploitation of domestic animals might be more related to dairy production, exploitation of hair/skin, or even labour power in the fields or for transport. Of the 6 cattle individuals analysed here, two were younger than 1 year, meanings they were used for meat, the same as 5 of the 7 ovicaprines that were between 9 and 12 months old. This indicates that they were indeed consumed, but this would be the result of the fact that to produce milk, cows and sheep have to reproduce, so this consumption would be more occasional, and as a consequence of the milk production process.

**Figure 6:**
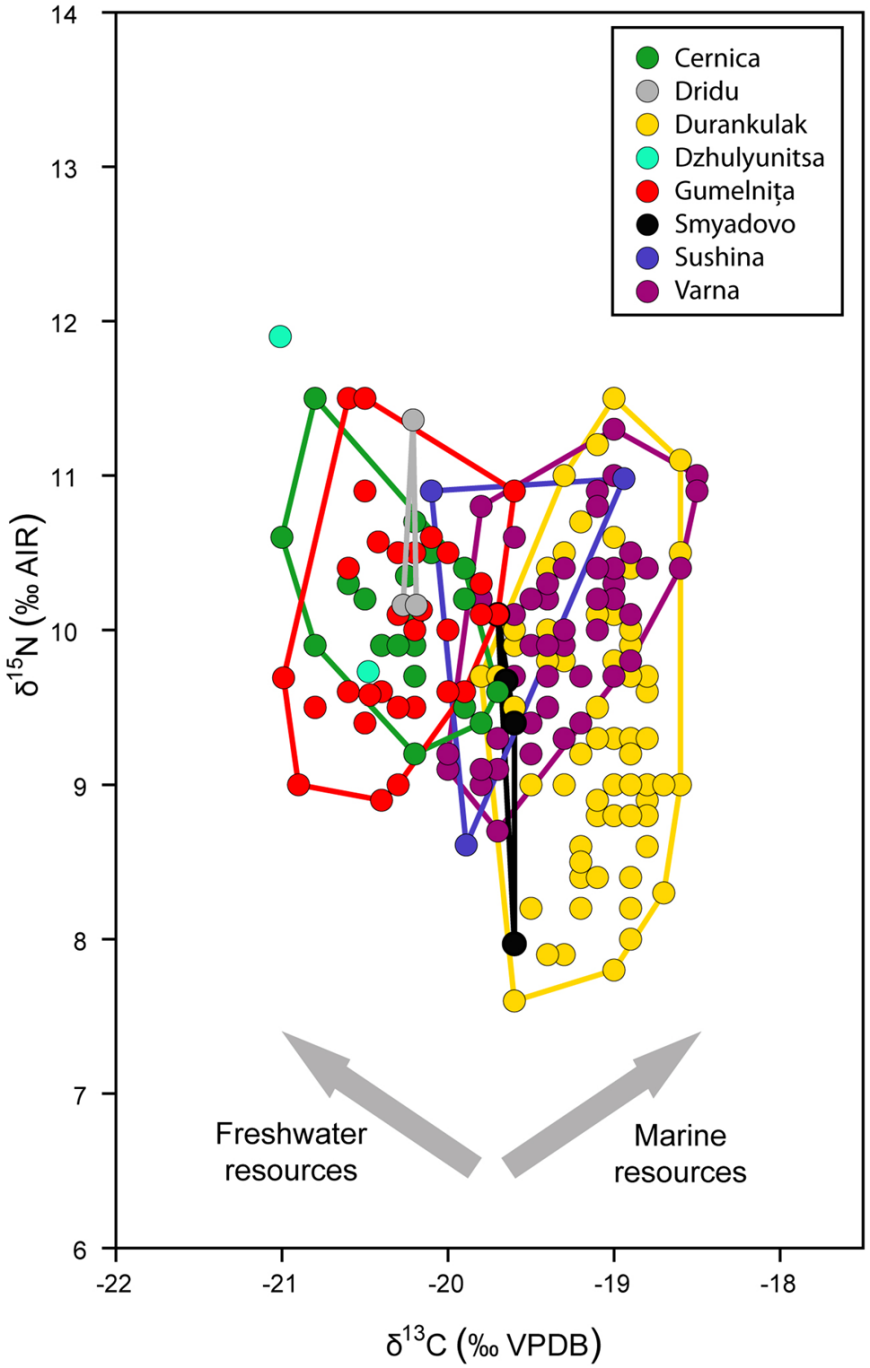
Isotopic comparison with other Neolithic sites from Romania and Bulgaria^6,58,62,129^ and their convex hulls. Lactating children and neonates have not been included in the analysis.

To establish if the people's diet from Gumelnița is different (or not) to other communities from the same area and period, we compared our data with those from other Neolithic sites in Romania and Bulgaria (Fig. 6)^6,58,62,129^ (see Fig. 1a for locations). Thus, Cernica, Dridu, and Dzhulyunitsa overlap with Gumelnița, which means that the inhabitants’ diets could also be similar and that a quarter of the protein in their diets could come from freshwater resources. Employing FRUITS, it was established that Cernica population^129^ had between 13 and 19% of freshwater fish in their diet, which is a similar result to Gumelnița, but they have not taken into account freshwater mussels that increase the percentage of freshwater resources in the diet. Results shown in Fig. 6, suggest that the amount of protein from freshwater resources was similar between both populations. Cernica, Dridu and Gumelnița sites are all in the north of the Danube, and located close to rivers. Dzhulyunitsa is located south of the Danube, closer to the Balkan Mountains than the Danube, and near a river. Cernica has an older chronology (5355–4940 cal BC^129^) than Gumelnița, but that only supports the continuity of this type of diet at least since Boian communities.

Situated to the right of the plot are Durankulak and Varna. These two communities are from Hamangia and KGK VI archaeological signals^58,59^. Varna humans follow a linear relationship^59^ related to marine fish consumption^61^. In the case of Durankulak, some individuals overlap with Varna, so they probably had the same diet, but there are others with lower δ^15^N values but the same range in the δ^13^C values. These differences in δ^15^N values can be explained by diets with more percentage of plants, differences in the amount of manuring of the crops, or the consumption of marine molluscs instead of fish by the individuals with lower δ^15^N.

The KGK VI individuals from Smyadovo and Sushina cemeteries are in the middle of the plot. Smyadovo individuals have very similar δ^13^C values, but differences in δ^15^N values, and they overlap with the more negative δ^13^C values of Varna and Durankulak. Probably they have a more terrestrial diet than the other populations, but more data is needed. In the case of Sushina, we only have 3 data with 3 completely different values that point to different diets: one with more marine food that overlaps with Varna, another with freshwater resources (or game) that overlap with Gumelnița site, and the last one with more terrestrial that overlap with Durankulak. Smyadovo and Sushina are situated in the Balkan Mountains and probably had some communication with Varna or the Black Sea area.

Only one individual from the Gumelnița cemetery (M3/1962) was in the extended dorsal position^15^, specific for the KGK VI groups from the Black Sea area, also known as “Varna culture”. Considering the isotopic results, this individual has an average diet within the Gumelnița population. If this person was related to the Varna area, he certainly didn’t live there in the last years of his life since his diet is typical of the north of the Danube. A mobility study could clarify this issue.

Isotopic studies (δ^13^C, δ^15^N, δ^34^S) show that hunter-gatherers^130–134^ from Romania had a higher amount of animal protein in their diet than Neolithic people, especially with a higher amount of freshwater fishes^135^. The importance of aquatic resources in the diet seems to continue in the Neolithic and Chalcolithic, as we have seen with our results. Depending on the site location, these resources will come from rivers, lakes or the sea.

### Reservoir effect

**Figure 7:**
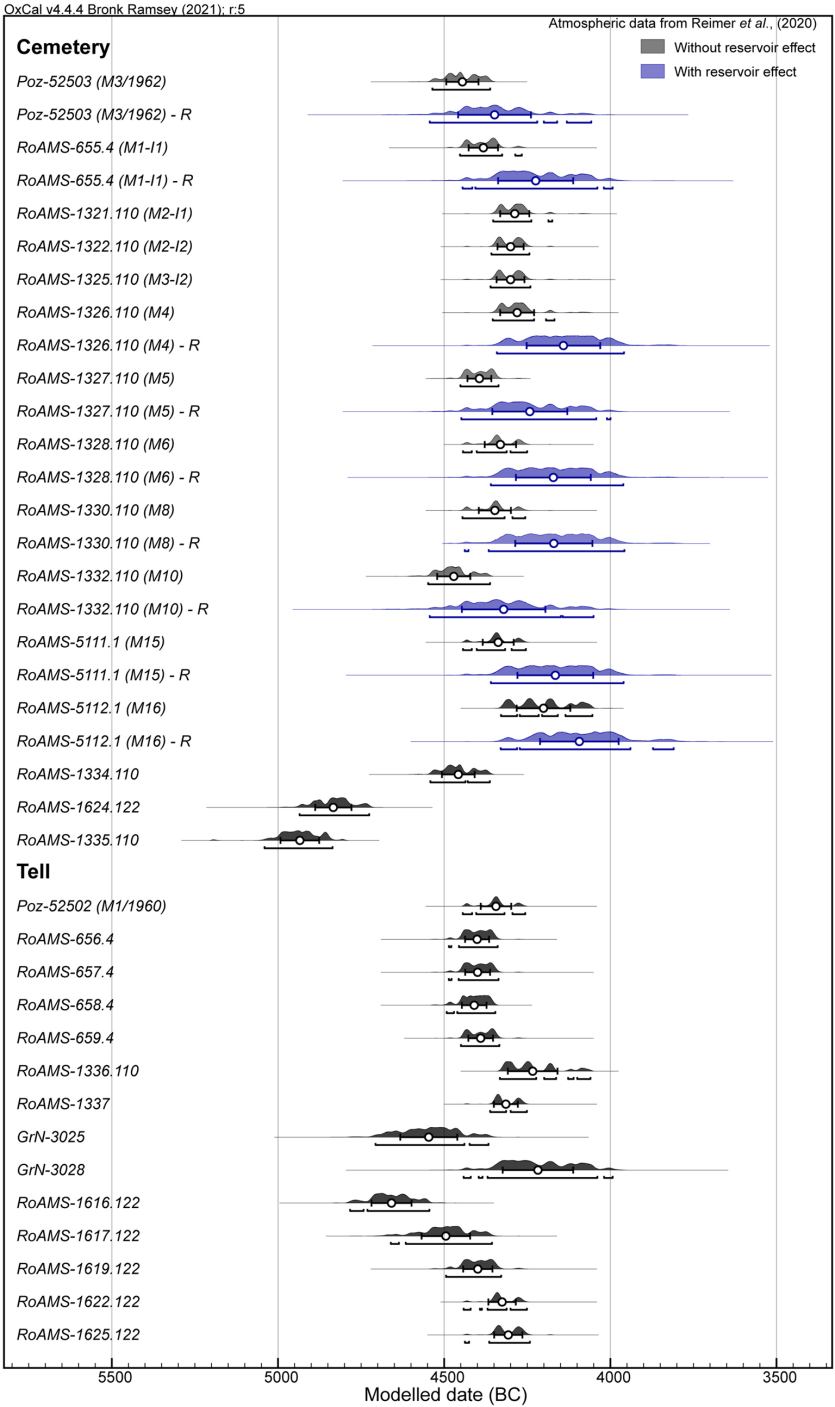
Radiocarbon data from the cemetery and the tell calibrated with Oxcal. R indicates that the radiocarbon was calibrated with the FRE (in blue). Mean (empty circle), σ and 2σ are represented in each of the radiocarbon dates.

The oldest radiocarbon dates from the cemetery are RoAMS-1335.110 and RoAMS-1624 from a pit (C7) assigned to Boian communities based on the specific archaeological signature (pottery with typical shapes and decorations of the Boian-Vidra ceramic style)^10^. The next is RoAMS-1616.122, a seed from C6, where pottery from Gumelnița A2 was found^10^. The youngest of the dates is M16. Whether we use the calibration with or without FRE, all human dates are within the Gumelnița archaeological period (Fig. 7). Interestingly M10 is the older raw human date, but applying the FRE, the older is M3/1962, the only grave where the skeleton is in an extended dorsal position.

Burials M15 and M16 were found in the same pit (feature C12) but deposited in overlapping soil layers, and one was placed in front of the other with complementary orientations (Fig. S3e). The difference between them in the mean of the uncalibrated dates is 126, calibrated with no FRE is 136, and with FRE is 72. These differences indicate that they were probably not contemporary, and M16 was buried later, but if the FRE of 540 ± 70 was not correct, M15 could have an earlier date that is masked by higher consumption of freshwater resources. Whatever it is, 2σ distributions of M15 and M16 with FRE overlap more than non-FRE distributions (Fig. 7). Whether we consider the FRE or not, this cemetery was used for at least 300 years. Burial M1-I1/1960 was found in the tell settlement and radiocarbon data of the child fits well into the cemetery ^14^C dataset. So, it seems that although most of the graves are extramural, KGK VI people still prefer to bury their dead inside the settlement, a funerary tradition that is related to the first Early Neolithic groups in the Balkans.

**Figure 8:**
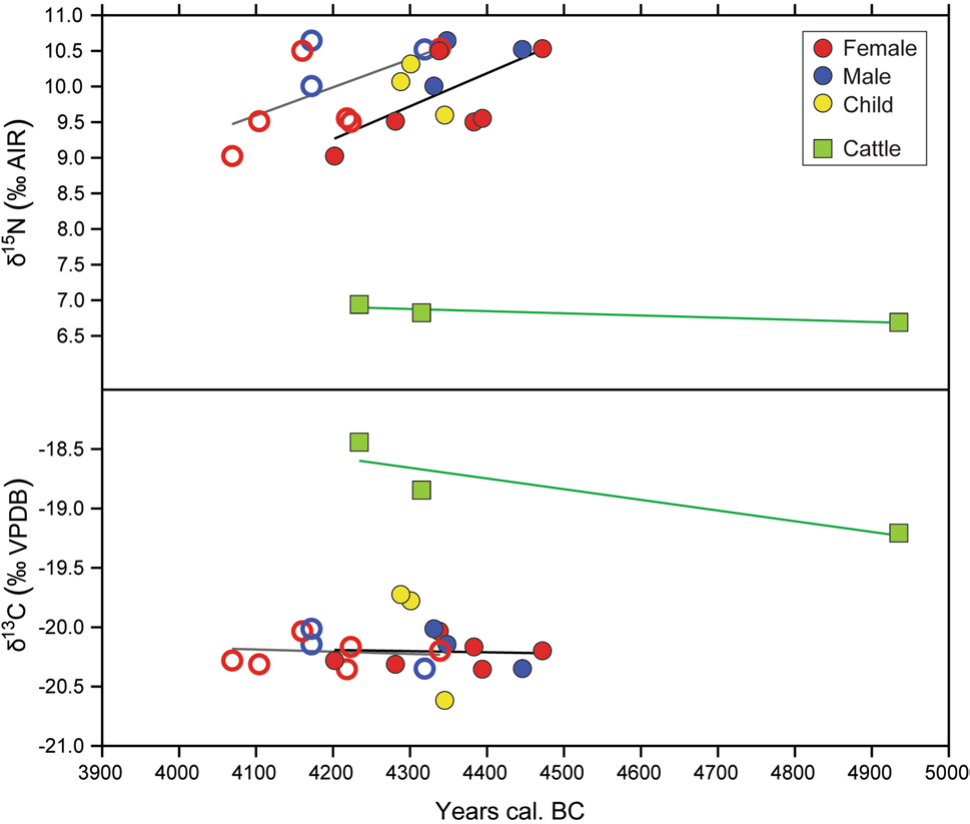
δ^15^N and δ^13^C vs. radiocarbon dating cal BC (means). Calibrated dates without the FRE are in the filled circles and squares and calibrated data with FRE are in the empty circles. Black line represents the regression of human calibrated dates without the FRE, grey line the regression of human calibrated dates with FRE, and green line the cattle regression.

In Fig. 8, the calibrated human radiocarbon dates with and without FRE and the cattle with available dating are represented in relation to their isotopic values.

### The resilience of the KGK VI community at Gumelnița site

The human group designed as KGK IV developed within the Atlantic period (6900–3700 cal BC), a warm-moist of Blytt and Sernander’s classification. At the end of this period (c. 4100–3700 cal BC), the climate got cooler and drier^136^. This transition towards the next period (Subboreal) was set by the 5.9 ka event (Bond event 4)^137^. This event has also been observed in lipid biomarkers, pollen and speleothems studies^138,139^ in the Carpathian mountains. During this period, along with fading of many KGK VI tell settlements, other contemporary "cultures" from this part of Europe collapsed too (e.g., Tiszapolgár in Transylvania; the end of Cucuteni A-B and the beginning of B1 phase in Moldova)^136^. However, in the case of KGK VI, some communities are more resilient than others, and the Gumelnița site is one of those.

In the 300 years covered by the cemetery radiocarbon dates, a decrease in δ^15^N values can be observed (Fig. 8), but this could be an artefact due to the sex of the individuals (the two youngest dates are females). Besides this, M16 (the youngest date) shows the lowest δ^15^N value of the adults, probably due to molar loss (Fig. S21c). The precipitation decreased in Romania from 4300 cal BC^136^, which coincides with the oldest graves (FRE). Aridity increases δ^15^N values, but the decrease in temperatures, also decreases δ^15^N values of soils^140^, producing opposite effects. The domestic plants baseline is not only affected by these climate changes but only by the manuring process, which is an anthropic one. Therefore, a decrease in agricultural activity could be behind these values, which could indicate the decline of the KGK VI civilization. Interestingly, this decrease in precipitation corresponds very well with the KGK VI population dynamics model recently proposed by Popescu et al.^8^, more precisely with the decline episode of these communities, which started at c. 4350 BC, without a significant recovery, until it vanished toward the end of the 5th millennium BC–early 4th millennium BC.

This situation proves that the Gumelnița settlers were a more resilient community than others, temporarily resisting the shock produced by climatic changes and economic-cultural collapse, which led to the disappearance of many KGK VI tell settlements up to 4200 cal BC^8^. The fact that the Gumelnița community resisted to the collapse, is also proven by the cemetery's radiocarbon data, which shows that people most likely survived until the beginning of the 4^th^ millennium BC, according to the corrected FRE (Table S8).

Besides the few data on cattle with ^14^C dates, these results show that δ^15^N values haven't changed since the Boian period. However, in the case of δ^13^C values, Gumelnița cattle turned towards those C4 values that were already mentioned. This could be a sign that the pastures of the Gumelnița site area could be less suitable for these animals, and they have to move with the herds at least some part of the year (maybe in summer, as it is the C4 growth period) to areas with wild C4 plants, or the trampled soil ecosystem with C4 plants^102^, developed near the settlement area during the KGK VI period. On the other hand, four “C4 episodes” were identified over the Balkans^141^ during the Late Pleistocene. Those episodes were related to dry and cold summers, which don't allow C3 plants to grow, thus supporting C4 development and considering the precipitation decrease in Romania at 4300 cal BC, along with temperature decrease, may have a "C4 episode" here but on a smaller scale, meaning that the percentage of C4 plants was higher during this period.

On the other hand, the fact that naked barley is the most frequent crop in the site, followed by hulled barley and einkorn, indicates another clue regarding Gumelnița people's resilience. Ecologically, barley is well adapted to dry conditions, and it is more susceptible to waterlogged conditions and vulnerable to low temperatures (compared to wheat). Hulled barley had a more significant environmental tolerance than naked barley, and it responded better to environmental changes. Einkorn can grow in cold and dry environments too. This shows a deliberate selection of the cultivated cereals by Gumelnița settlers, apparently species resistant to climate change stress.

Moreover, the documented wild plant species at the Gumelnița site (e.g., *Prunus* type, followed by blackberry and hazelnut) are not that susceptible to climate change. For example, some *Prunus* species are adaptable to various conditions, except light. The blackberry is also a very adaptable plant; hazelnuts can resist cold and high temperatures.

## Conclusions

The Gumelnița cemetery was used for more than 300 years (4545–4179 cal BC) and corresponded to the period of use of the even settlement (4800–4040 cal BC). If the entire chronological sequence of KGK VI is documented on the tell, the cemetery's radiocarbon data reflects only the field research stage. The human remains buried here reflect the maximum period of development of KGK VI communities (4500–4400 cal BC)^8^ but also the fast decline period that followed it and marked the collapse of this flourish VI civilization^,^^8^. The FRE has been applied in the calibration of radiocarbon dates on humans. Although the range obtained is larger than in the calibrations without the FRE, on average, there is a lapse of 147 years between these calibrations that not affect the archaeological interpretation of the features presented here.

Overall, due to taxonomically diverse material and a consistent chronology from Gumelnița site, the analysis of δ^15^N and δ^13^C has allowed us to get a closer look at the people’s way of life of at the Gumelnița site. The first observation is that these people had a terrestrial diet (74%) with a consistent contribution to aquatic resources (26%). Regarding terrestrial resources, we note that domestic plants (52%) play the most crucial role in feeding these people, followed by terrestrial mammals (22%). The diet of the KGK VI population was supplemented with aquatic resources, more specifically shellfish (18%) and fish (8%).

Considering this data, we could conclude that the KGK VI community from Gumelnița was an agrarian society in which half of the protein of the diet came from cereal crops (wheat and barley) and legumes (lentils and peas). The rest of the protein came mainly from freshwater resources (freshwater mussels and fish) and hunting, with meat from livestock being a more occasional resource. Finally, domestic herbivores would be exploited primarily to obtain secondary resources such as dairy products, fur, manure and labour power, an activity not yet demonstrated for the Chalcolithic period in the Eastern Balkans^142^, but in the Western Balkans^143^.

However, the diet profile of the Gumelnița population seems to be dominant in northern side of the Danube River, and it has existed already since Boian and Hamangia archaeological groups^144^, and it is related to the arrival of Anatolian Neolithic farmers in the area^6^. This situation reflects the perpetuation of culinary traditions, between generations, over several hundred years between these human groups.

The agricultural practices of the KGK VI community from Gumelnița involve manured and irrigated crops, highlighting the use of this manure in legumes, this being the first direct evidence of these processes within KGK VI human groups. Cereal chaff and other crops wastes were used to feed cows and sheep. Also, the dogs and pigs consumed human waste (according to isotopic signature), although the latter had a less carnivorous diet, with different degrees. The foxes appear to have had a diet similar to that of the dogs, indicating possible synanthropic behaviour, a situation which is also for the first time documented in KGK VI area.

Human dietary data does not seem to indicate that there were different classes or ranks within this Chalcolithic society, so it looks pretty egalitarian, although there is a slight difference between the animal protein consumed by men and women, which could be the effect of the particular preferences based on physiological differences. There is no gender difference of grave goods either, which in this society seems quite scarce. In the case of the diet variations on gender classes, we observe some minor differences between men and women, which consist in a slightly higher δ^15^N in men. Also, for age categories, there is a decrease in δ^15^N from infants to adolescents determined by lactation and later by active growth. Once adulthood is reached, the diet is homogenous, and is determined by small variations based by own tastes and physiology either due to gender or pregnancy and lactation. Older individuals appear to have a small decrease in δ^15^N, however, in the case of M16, with premortem loss of molars, her diet would have softer foods such as plants or fish.

On the other hand, slight dietary variations noticed in the human population from the Gumelnița site may reflect the stages of development and decline of the KGK VI civilization. This situation proves that the Gumelnița settlers were a more resilient community than others, temporarily resisting the stress produced by climatic changes and economic-cultural collapse, which led to the disappearance of most KGK VI tell settlements between 4350 cal BC to 4200 cal BC^8^. The fact that the Gumelnița community resisted better to the collapse was due to human adaptation to the new climate conditions (precipitation decreased after 4300 cal BC^136^) that led to the abandonment of most of the KGK VI tell settlements after this moment. Thus, being an agrarian community, which had a diet based on plants, as we have shown in this study, the Gumelnița people, to support the crops, used intensive soil fertilization and crop watering alongside the selection of cereal species resistant to climate change stress, as proven by our data. In this way, the community managed to survive for several hundred years more than the other KGK VI settlements, as shown in ^14^C data from the cemetery (Table S8). However, the KGK VI decline episode was a continuous process spanning almost 550 years (c. 4350–3800 BC)^8^, and Gumelnița community resilience was only a temporary solution. The socio-economic recovery of it never happened.

Finally, we do not exclude those human dietary variations recorded at the Gumelnița site be the result of particular traditions of different human individuals related to the place of their origin or the paleogenetic background documented for KGK VI (e.g., Anatolian Neolithic farmers, Balkan hunter-gatherers or people with steppe-related ancestry), but this correlation will be made into a further study. Thus, the next steps will be represented by isotopic investigations related to the mobility (δ^18^O, ^87^Sr/^86^Sr) of these individuals, doubled by paleogenetic analyses, in order to increase the accuracy of the lifestyle reconstruction model of the KGK VI human groups.

## Supplementary Information

Supplementary Information.

## Data Availability

The data sets supporting the conclusions of this article are included within the article and in the Supplementary data file.
