# Supplementary material for: Unravelling the resilience of the KGK VI population from the Gumelnița site (Romania) through stable isotopes
Source: Sci Rep. 2023 May 25;13:8499. doi: 10.1038/s41598-023-35129-0 (PMC10213031; doi:10.1038/s41598-023-35129-0)
Supplement: Supplementary file 1 — Supplementary Information. [file 41598_2023_35129_MOESM1_ESM.pdf]

SUPPLEMENTARY MATERIAL

Unravelling the resilience of the KGK VI population from the Gumelnița site (Romania) through stable isotopes

Ana García-Vázquez, Adrian Bălășescu, Gabriel Vasile, Mihaela Golea, Valentin Radu, Vasile Opriș, Theodor Ignat, Mihaela Culea, Cristina Covătaru, Gabriela Sava, Cătălin Lazăr

Table of contents **Page**

Section 1. Gumelnița site 3
Figure S1. Gumelnița tell and excavations (2017-2019) 3
Figure S2. Graves M1-M11 4
Figure S3. Graves M12-M16 and C12 5
Figure S4. Graves M17, M18, M21 and M22 6
Table S1. Plant macroremains 7
Table S2. Animal remains 9
Section 2. Plant samples pre-treatment 10
Figure S5. FTIR-ATR spectra 10
Section 3. Fish collagen 11
Section 4. Cluster 12
Figure S6. Clusters of the human population from Gumelnița 12
Section 5. FRUITS Bayesian modelling 13
Section 5.1. Model settings 13
Section 5.2. Results 14
Table S7. FRUITS results 14
Figure S7. FRUITS box plots (all individuals) 15
Figure S8. M1-I1 15
Figure S9. M3-I1 16
Figure S10. M4 16
Figure S11. M5 16
Figure S12. M6 17

Figure S13. M7 ... 17
Figure S14. M8 ... 17
Figure S15. M9 ... 18
Figure S16. M10 ... 18
Figure S17. M11 ... 18
Figure S18. M12 ... 19
Figure S16. M14 ... 19
Figure S20. M15 ... 19
Figure S21. M16 ... 20
Figure S22. C12-I1 ... 20
Figure S23. C12-I3 ... 21
Figure S24. C12-I4 ... 21
Figure S25. M17 ... 21
Figure S26. M18 ... 22
Figure S27. M21 ... 22
Figure S28. M22 ... 22
Figure S29. M1-I2/1960 ... 23
Figure S30. M1/1962 ... 23
Figure S31. M2/1962 ... 23
Figure S32. M3/1962 ... 24
Section 6. Radiocarbon datings ... 25
Table S8. Radiocarbon datings and reservoir effect ... 25
References ... 27

Section 1. Gumelnița site

Figure S1. Gumelnița tell and excavations (2017-2019)

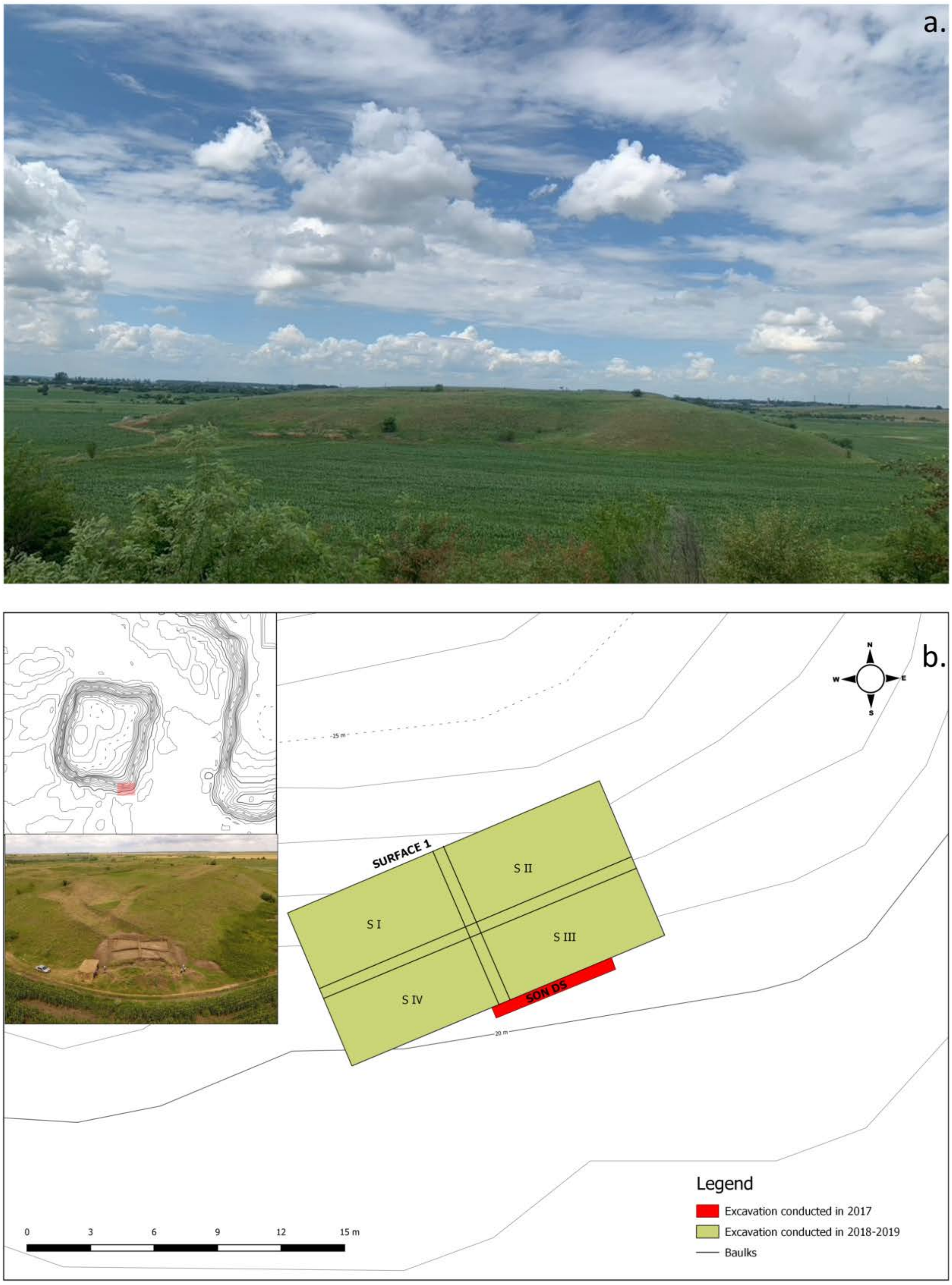

Figure S1. a) Gumelnița tell settlement. b) Excavation areas conducted in Zone 1 of the Gumelnița site.

Figure S2. Graves M1-M11

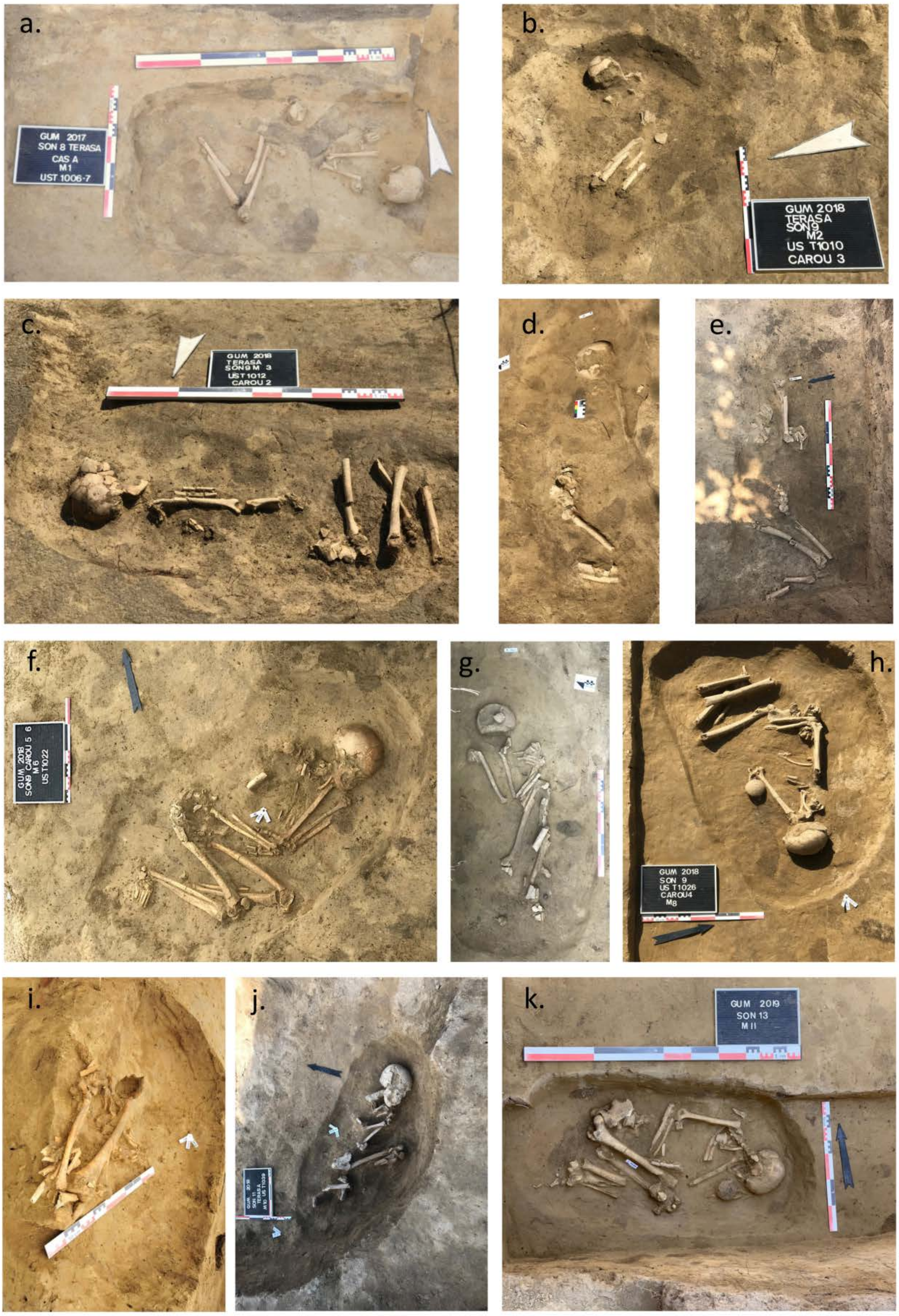

Figure S2. a) M1. b) M2. c) M3. d) M4. e) M5. f) M6 g) M7. h) M8. i) M9. j) M10. k) M11.

Figure S3. Graves M12-M16 and C12

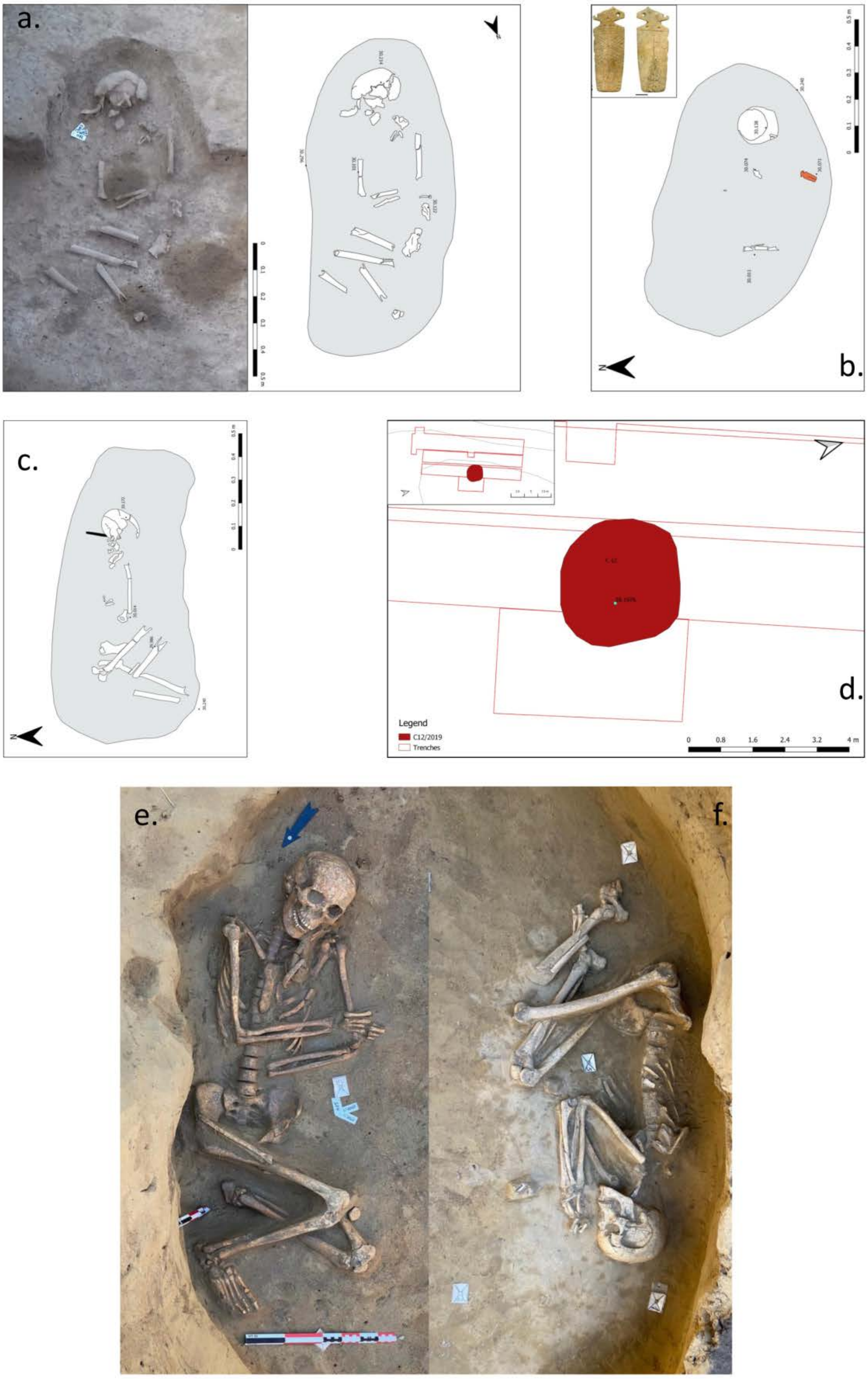

Figure S3. a) M12. b) M13. c) M14. d) C12. e) M15 on the left and M16 on the right, positioned in overlapping layers.

Figure S4. Graves M17, M18, M21 and M22

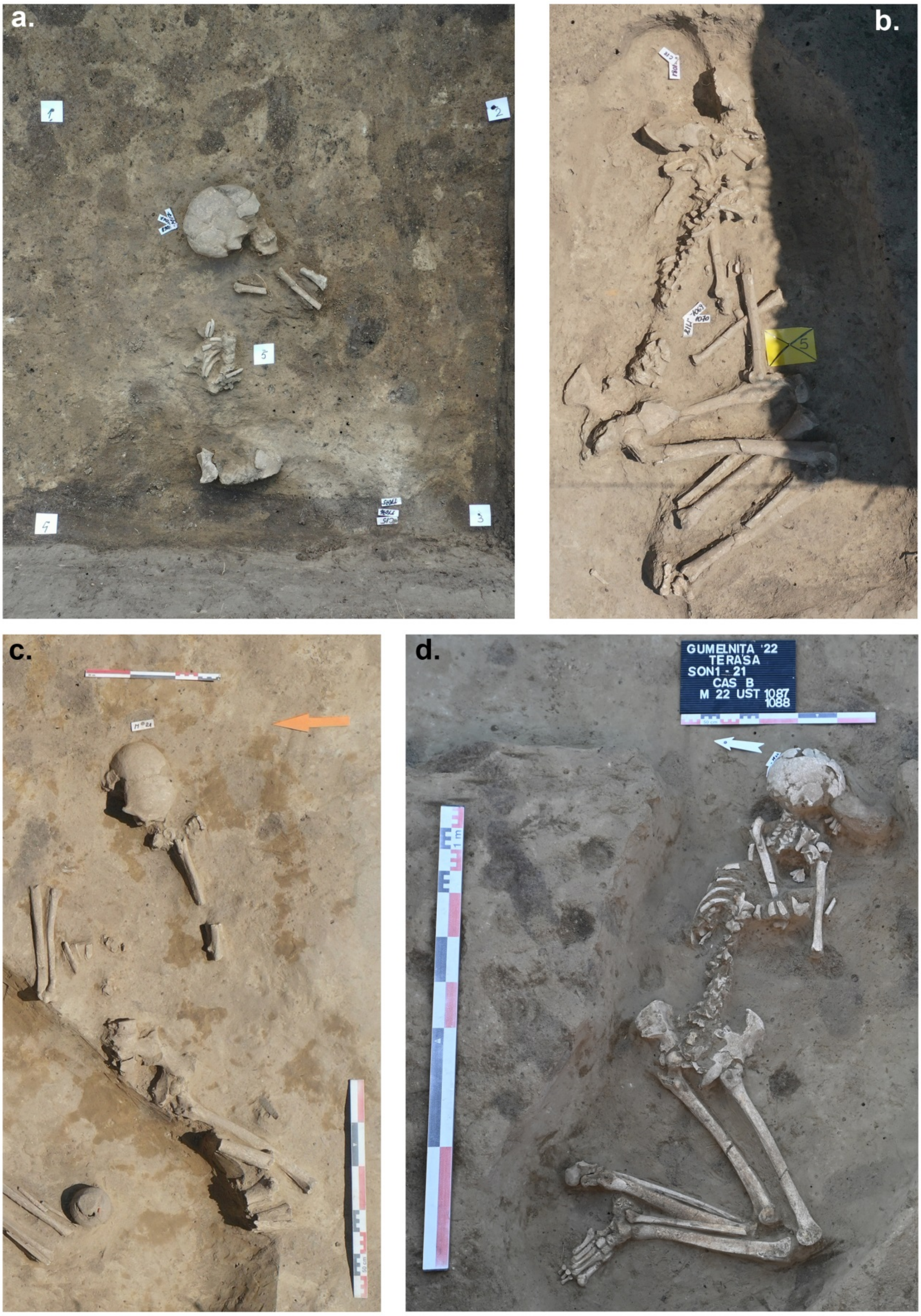

Figure S4. a) M17. b) M18. c) M21. d) M22.

Table S1. Plant macroremains

Table S1. Plant macroremains found in the excavations of 2017-2019. Data from [1,2].

	Tell (Zone 1)									Cemetery (Zone 3)									
**Species**	**L1**	**C4**	**C5**	**C3**	**C2**	**C6**	**A7-8**	**A8**	**A7**	**M5**	**M6**	**M7**	**M8**	**M9**	**M10**	**M14**	**C9**	**C12**	**Total**
**Cereals**																			
Poaceae							10	5	18		3			4	8	1	2	45	96
Cerealia	10	7	17	3	7	2	1												47
Triticum monococcum	2		2	1		1	2		5					2				2	17
Triticum dicoccum	6	2	2		1				1										12
Triticum. naked									1										1
Triticum sp.	2		1				1		4		1			2	3			2	16
Triticum cf. *spelta*							1												1
Hordeum vulgare vulgare		1	1	1	1	2	1		2						1			12	22
Hordeum vulgare nudum	4	2	1		2		3	2	1									7	22
Hordeum sp.	2	1	3			1	1	1	10	2	1			3	2			12	39
Secale cereale	2			1															3
Bromus sp.										1				1					2
Bromus /*Stipa*														1					1
**Pulses**																			
Fabaceae							2	1	3						1				7
Pisum sativum						1					2								3
Pisum sp.	1																	2	3
Lens culinaris	2		1			1													4
Vicia ervilia		1							4										5
Vicia sp.	1						3												4
Lathyrus sp.									2										2
**Other plants**																			
Quercus rubur pedunculiflora	1																		1
Prunus cerasifera		1		4															5
Prunus sp.	5	4	6	1	4	3	1		4	1				3					32
Sambucus nigra	2			1	1	8													12
Vitis sp.	1																		1
Polygonum aviculare	1	1																	2
Rumex acetosa	1																		1
Chenopodium album																	1		1

Brassica sp.														1					1
Rubus cf. *fruticosus*						2													2
Undetermined macroremains							14	4	8	5	4	3	15	16					69
																		Total	434

Table S2. Animal remains

Table S2. Animal bone remains recovered during the excavations of 2017-2019. NISP: number of identified specimens. MNI: minimum number of individuals

Specie	Lazăr *et al.* (2017) [1]	Lazăr *et al.* (2020) [2]	Total NISP	%	MNI
**Mammals**					
Bos taurus	78	311	389	41.47	7
Ovis aries	2	20	22	2.35	7
Capra hircus		3	3	0.32	1
Ovis aries/Capra hircus	60	222	282	30.06	2
Sus domesticus	6	79	85	9.06	5
Canis familiaris	9	53	62	6.61	6
Equus ferus		3	3	0.32	1
Bos primigenius	2	4	6	0.64	1
Cervus elaphus	2	4	6	0.64	1
Capreolus capreolus		4	4	0.43	1
Sus scrofa	5	16	21	2.24	2
Vulpes vulpes	2	2	4	0.43	2
Meles meles		2	2	0.21	1
Lepus europaeus		3	3	0.32	1
Castor fiber		2	2	0.21	1
Bos sp.	5	4	9	0.96	1
Sus sp.	13	22	35	3.73	2
**Total mammals determined**	**184**	**754**	**938**	**100.00**	**42**
Mammals big size undetermined	72	254	326		
Mammals medium size undetermined	84	237	321		
**Total mammals**	**340**	**1245**	**1585**		**42**
**Molluscs**					
Unio tumidus	35	91	126	21.65	76
Unio pictorum	21	20	41	7.04	22
Unio crassus	3	19	22	3.78	12
Unio sp.	34	144	178	30.58	113
Anodonta sp.	38	137	175	30.07	98
Psudoanodonta sp.		1	1	0.17	1
Viviparus sp.	4	30	34	5.84	34
Dreissena sp.	1		1	0.17	1
Cepaea vindobonensis		4	4	0.69	4
**Total molluscs**	**136**	**446**	**582**	**100.00**	**361**
**Fish**					
Esox lucius	1	3	4	8.16	3
Cyprinus carpio	3	12	15	30.61	6
Silurus glanis	2	13	15	30.61	6
Fish undetermined	2	13	15	30.61	
**Total fish**	**8**	**41**	**49**	**100.00**	**15**
**Reptiles**					
Emys orbicularis	1	22	23		7
**Total freshwater**	**289**	**996**	**1285**		**383**

Section 2. Plant samples pre-treatment

Figure S5. FTIR-ATR spectra

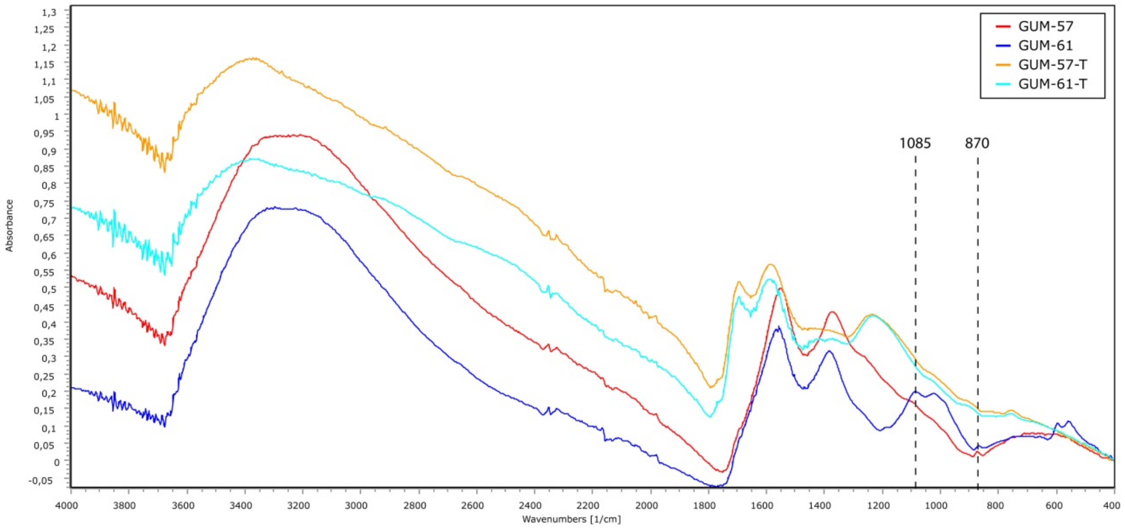

Figure S5. FTIR-ATR spectra of GUM-57 and GUM-61 before the pre-treatment, and the same samples but treated (GUM-57-T and GUM-62-T). The peaks that show contamination (870 in carbonates, and 1085 in nitrates) are also represented.

Section 3. Fish collagen

Due to the poor results of the method of Bocherens *et al.* (1991) [3] (**R0**) in the fish from Gumelnița (Table S4: R0), four variations of it have been tested with the sample GUM-49 (Table S3):

- **R1**. 500 mg of bone powder. Centrifugation instead of filtration.
- **R2**. 500 mg of bone powder. Acid-acid (suppressing the NaOH step).
- **R3**. 500 mg of bone powder. The NaOH step only 45 min instead 20h, similar to Balasse *et al.* (2016) [4].
- **R4**. 500 mg of bone powder, and then R0.

Table S3. Results of the different methods on GUM-49. Two measurements have been done for all the samples except in GUM-49-R4. In red are the parameters that are outside the range of the quality criteria for collagen [5–8].

Sample	Method	$\delta^{15}N$	%N	$\delta^{13}C$	%C	Yield %	C:N
GUM-49	R0	-	-	–25.6	1.92	5.8	-
GUM-49-R1	R1	10.2	2.11	–19.8	5.20	0.4	2.9
GUM-49-R2	R2	11.2	9.00	–20.4	26.25	5.2	3.4
GUM-49-R3	R3	11.0	8.65	–19.8	23.05	1.1	3.1
GUM-49-R4	R4	10.6	4.90	–20.5	14.60	-	3.5

From all these variations, the one that had better results is the acid-acid (R2) as the sample (GUM-49-R2) meets all quality criteria and also the %C and the %N have higher values. Interestingly with the double amount of bone powder (R4), we obtained acceptable results, but looking at the percentages of C and N, we obtained almost half than using the R2 method.

Table S4. R0, R1 and R2 methods were applied to all the samples. In red, the values that do not meet the quality criteria.

Sample	R0 [3]					R1 (centrifugation)					R2 (A-A)				
	%N	$\delta^{15}N$ (‰)	%C	$\delta^{13}C$ (‰)	C:N	%N	$\delta^{15}N$ (‰)	%C	$\delta^{13}C$ (‰)	C:N	%N	$\delta^{15}N$ (‰)	%C	$\delta^{13}C$ (‰)	C:N
GUM-47	-	-	4.01	–27.4	-	1.90	6.4	4.55	–24.0	2.8	2.96	7.5	9.07	–24.2	3.6
GUM-48	-	-	2.92	–26.7	-	2.50	6.0	6.17	–25.2	2.9	8.53	5.1	25.24	–26.0	3.4
GUM-49	-	-	1.92	–25.6	-	2.11	10.2	5.20	–19.8	2.9	9.00	11.2	26.25	–20.4	3.4
GUM-50	-	-	3.02	–26.4	-	1.53	10.7	3.43	–19.9	2.6	3.65	11.4	9.87	–19.7	3.2
GUM-51	-	-	-	-	-	2.69	10.4	6.58	–20.7	2.9	5.32	10.8	14.86	–21.0	3.3
GUM-52	-	-	-	-	-	0.82	-	1.5	-	2.2	2.34	9.5	6.32	–21.8	3.2
GUM-53	-	-	2.40	–25.5	-	3.64	9.5	9.3	–23.9	3.0	6.82	9.2	18.91	–24.1	3.2

We obtained better percentages of carbon and nitrogen with the A-A method (R2) in relation to the Bocherens *et al.* (1991) [3] method (R0) and the centrifugation (R1) (Table S4). It was also possible to obtain good results on samples that were rejected before, but still in three of the cases (GUM-47, GUM-50 and GUM-52) quality criteria were not fulfilled, maybe due to bad preservation.

Section 4. Cluster

Figure S6. Clusters of the human population from Gumelnița

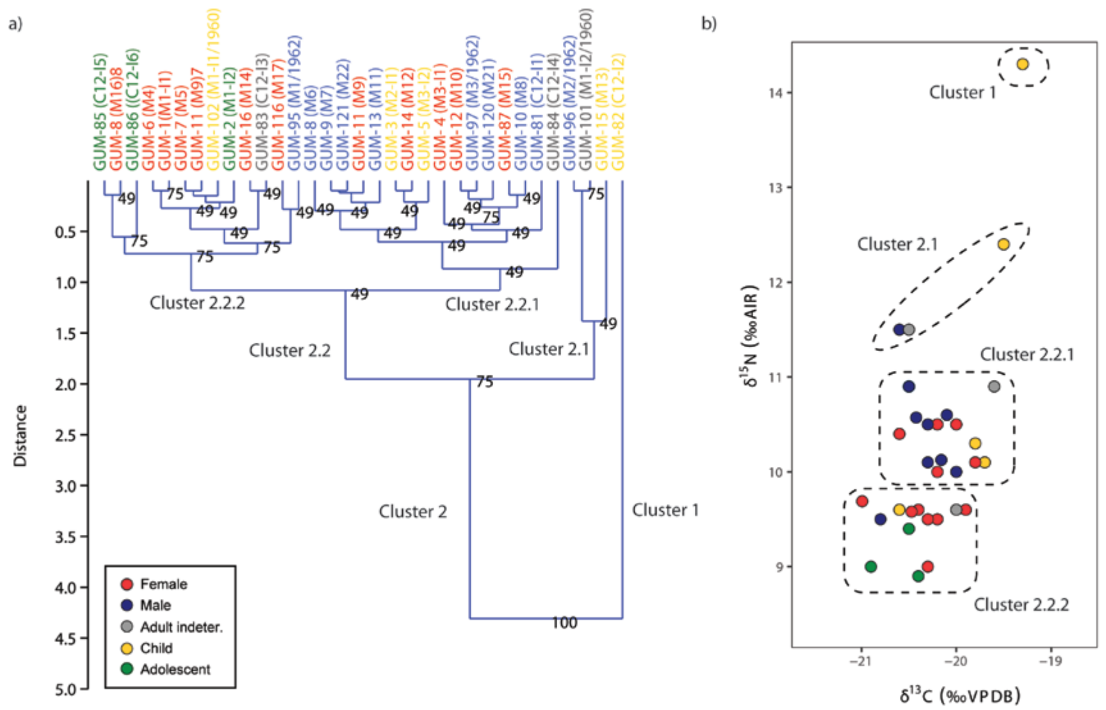

Figure S6. a) Hierarchical clustering using UPGMA algorithm with boot N=5000. b) Isotopic bivariate plot of human isotopic results and the representation of the clusters from a).

Section 5. FRUITS Bayesian modelling

Section 5.1. Model settings

- **Dietary proxies** are $\delta^{13}C$ and $\delta^{15}N$ and the analysed **food fractions** are protein and energy.
- The **consumer data** are the Gumelnița adults only because we wanted to avoid errors or attribute higher or lower trophic levels due to growth or breastfeeding.
- We have chosen 4 **food groups**: terrestrial animals, fish, shellfish and domestic plants. For each food group, the mean $\delta^{13}C$ and $\delta^{15}N$ values of protein and energy were estimated using fractionations reported by Fernandes (2016) [9]. The isotopic composition of the macronutrients is derived from the average $\delta^{13}C$ and $\delta^{15}N$ values using the following offsets for mammals: $\Delta^{13}C_{protein\text{-}collagen} = -2‰$, $\Delta^{13}C_{lipids\text{-}collagen} = -8‰$ and $\Delta^{15}N_{protein\text{-}collagen} = +2‰$; for fish: $\Delta^{13}C_{protein\text{-}collagen} = -1‰$, $\Delta^{13}C_{lipids\text{-}collagen} = -7‰$) and $\Delta^{15}N_{protein\text{-}collagen} = +2‰$; and for plants: $\Delta^{13}C_{bulk\text{-}protein} = -2‰$ and $\Delta^{13}C_{bulk\text{-}carbohydrate} = +0.5‰$. No tissue offsets were required for shellfish protein values [10], and $\Delta^{13}C_{lipid\text{-}protein}$ is calculated to be −3.5‰ [11].
- The **offsets** are the recommended by Fernandes *et al.* (2015) [12]: human diet-to-collagen enrichment factors of +4.8 ± 0.5‰ for $\delta^{13}C$ and +5.5 ± 0.5‰ for $\delta^{15}N$. Collagen carbon is routed from 74 ± 4% of dietary protein carbon and the remaining 26% from carbohydrates and lipids [13]. Collagen nitrogen only comes from proteins.
- **Food values** (Table S5) derived from our isotopic results applying the offsets described before:

Table S5. Food values for FRUITS model.

Food Group	Fraction	$\delta^{13}C$ (‰)		$\delta^{15}N$ (‰)	
Terrestrial animals	Protein	–21.6	1.26	7.5	1.3
	Carbs/Lipids	–27.6	1.26		
Fish	Protein	–23.4	2.3	11.2	2.2
	Carbs/Lipids	–29.4	2.3		
Shellfish	Protein	–25	2.1	8.5	0.7
	Carbs/Lipids	–28.5	2.1		
Domestic plants	Protein	–26.4	1.7	6.4	2.4
	Carbs/Lipids	–23.9	1.7		

- **Concentrations** of the food fractions in the different food groups (Table S6):

Table S6. The concentration of the different food fractions in each food group.

Food Group	Protein	Energy (lipids, carbohydrates)	References
Terrestrial mammals	30 ± 4	70 ± 4	[12]
Fish	65 ± 5	35 ± 5	[12]
Shellfish	10 ± 2.5	90 ± 2.5	[14]
Domestic plants	10 ± 2.5	90 ± 2.5	[12]

- **Prior info**: ([Protein]/([Protein]+[Energy])) > 0.05; ([Protein]/([Protein]+[Energy])) < 0.4 [15].

Section 5.2. Results

Table S7. FRUITS results

Table S7. FRUIT results (%) and reservoir effect calculation using 545 ± 70 ^{14}C years for a 100% freshwater diet [16].

Grave	Terrestrial animals	Fish	Shellfish	Domestic plants	Freshwater total	Reservoir effect (years)
M1-I1	18 ± 19	6 ± 10	16 ± 13	60 ± 24	22 ± 16	118 ± 88
M3-I1	20 ± 19	9 ± 11	20 ± 15	52 ± 26	28 ± 19	152 ± 102
M4	18 ± 19	8 ± 11	15 ± 12	60 ± 25	23 ± 17	122 ± 90
M5	18 ± 20	7 ± 10	15 ± 13	60 ± 25	22 ± 16	118 ± 88
M6	24 ± 22	8 ± 11	17 ± 14	50 ± 26	25 ± 18	137 ± 96
M7	22 ± 21	8 ± 12	18 ± 14	51 ± 26	27 ± 18	144 ± 100
M8	26 ± 21	8 ± 11	20 ± 15	46 ± 25	28 ± 19	151 ± 100
M9	21 ± 20	8 ± 11	17 ± 14	53 ± 25	25 ± 18	137 ± 97
M10	26 ± 22	9 ± 11	20 ± 15	46 ± 25	28 ± 19	154 ± 100
M11	22 ± 21	8 ± 12	18 ± 14	51 ± 26	27 ± 18	144 ± 100
M12	26 ± 22	8 ± 10	17 ± 13	49 ± 26	24 ± 17	131 ± 92
M14	22 ± 22	8 ± 12	16 ± 13	54 ± 26	24 ± 17	129 ± 94
C12-I1	24 ± 21	9 ± 11	22 ± 16	45 ± 25	31 ± 20	170 ± 107
C12-I3	21 ± 21	7 ± 11	16 ± 13	56 ± 25	23 ± 17	123 ± 91
C12-I4	32 ± 22	9 ± 11	19 ± 15	40 ± 24	29 ± 19	154 ± 101
M15	26 ± 22	9 ± 11	19 ± 15	47 ± 26	27 ± 18	148 ± 100
M16	15 ± 19	6 ± 10	15 ± 13	64 ± 24	21 ± 16	112 ± 88
M17	14 ± 16	8 ± 11	17 ± 14	61 ± 24	25 ± 18	134 ± 97
M18	17 ± 18	7 ± 11	16 ± 13	60 ± 24	24 ± 17	127 ± 93
M21	23 ± 21	9 ± 11	20 ± 16	48 ± 26	29 ± 19	156 ± 104
M22	20 ± 20	8 ± 11	18 ± 14	53 ± 25	26 ± 18	141 ± 97
M1-I2/1960	24 ± 20	10 ± 11	26 ± 18	40 ± 24	36 ± 21	194 ± 116
M1/1962	24 ± 21	9 ± 11	19 ± 15	48 ± 26	28 ± 19	152 ± 102
M2/1962	25 ± 20	10 ± 11	24 ± 18	40 ± 24	35 ± 21	187 ± 114
M3/1962	15 ± 17	6 ± 10	16 ± 13	63 ± 24	22 ± 16	117 ± 87
Average	22	8	18	52	26	142

Figure S7. FRUITS box plots (all individuals)

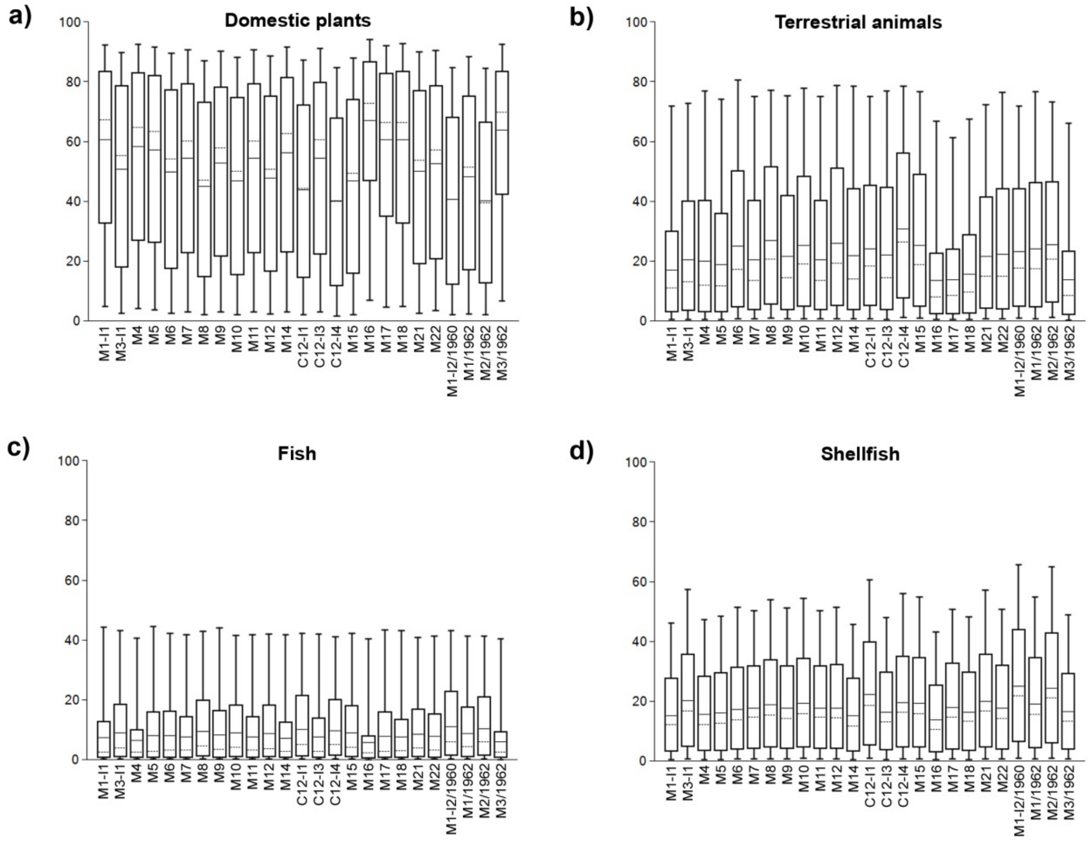

Figure S7. FRUITS results of all the adults from Gumelnița on a) domestic plants, b) terrestrial animals, c) fish and d) shellfish.

Figure S8. M1-I1

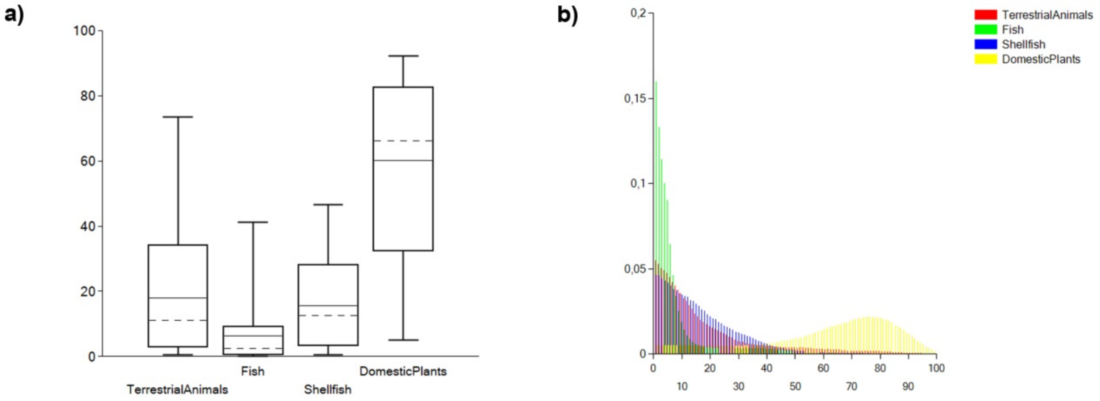

Figure S8. M1-I1 FRUITS results. a) Box plots. b) Probably distributions.

Figure S9. M3-I1

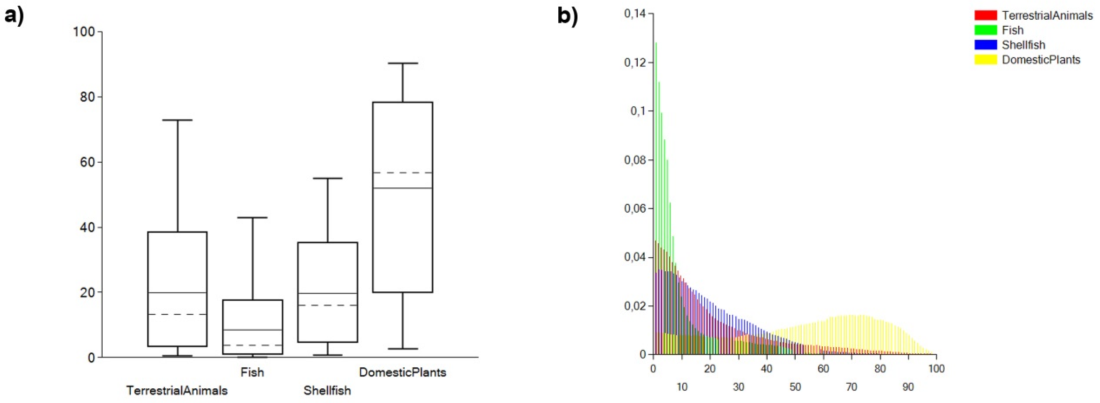

Figure S9. M3-I1 FRUITS results. a) Box plots. b) Probably distributions.

Figure S10. M4

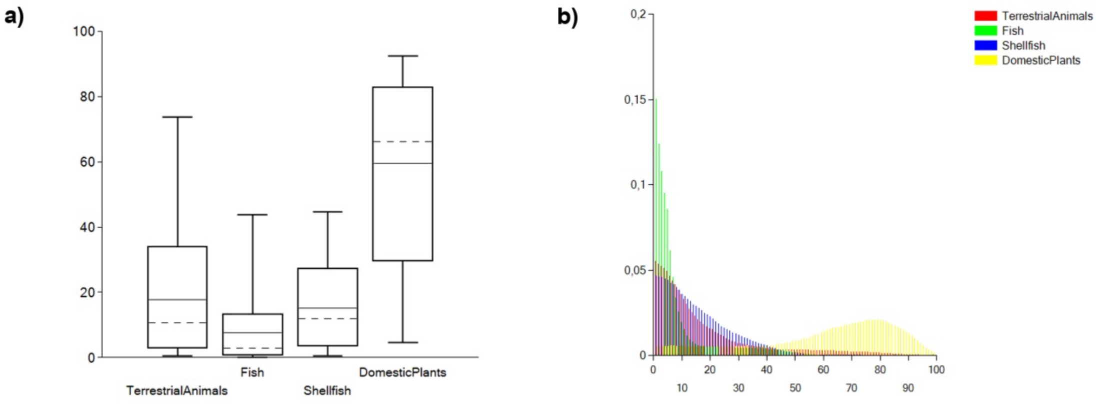

Figure S10. M4 FRUITS results. a) Box plots. b) Probably distributions.

Figure S11. M5

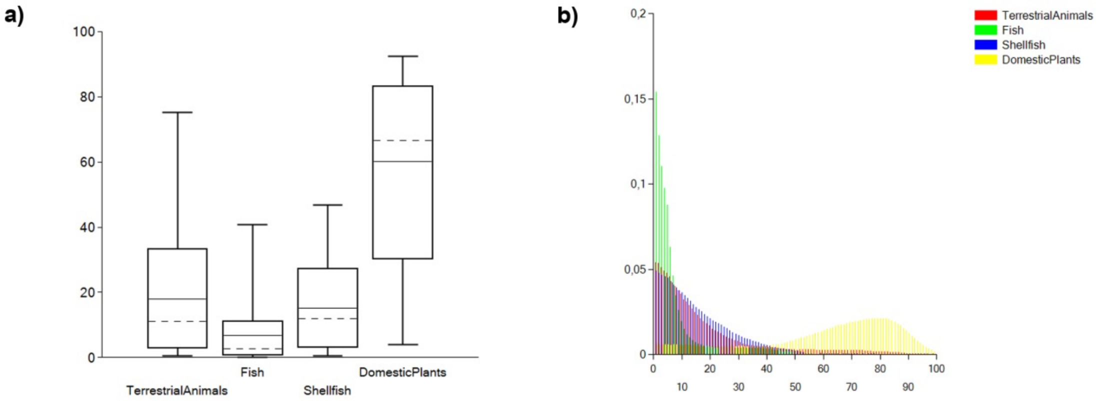

Figure S11. M5 FRUITS results. a) Box plots. b) Probably distributions.

Figure S12. M6

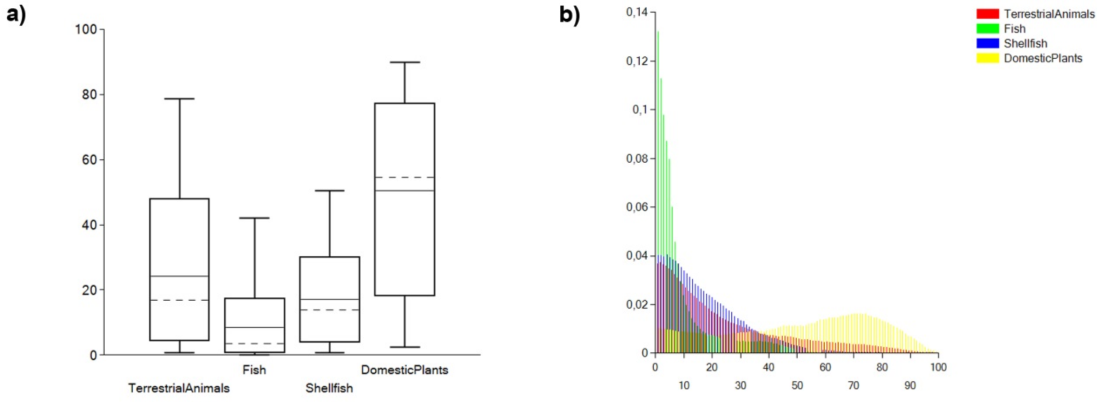

Figure S12. M6 FRUITS results. a) Box plots. b) Probably distributions.

Figure S13. M7

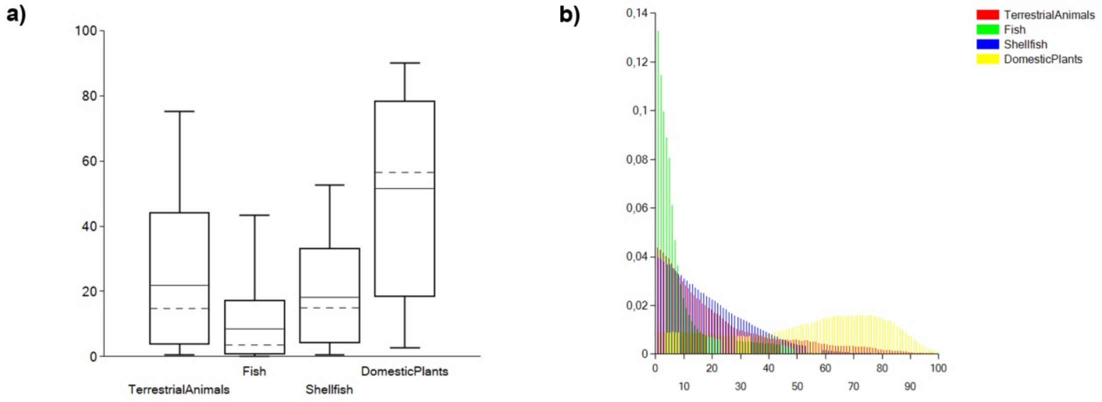

Figure S13. M7 FRUITS results. a) Box plots. b) Probably distributions.

Figure S14. M8

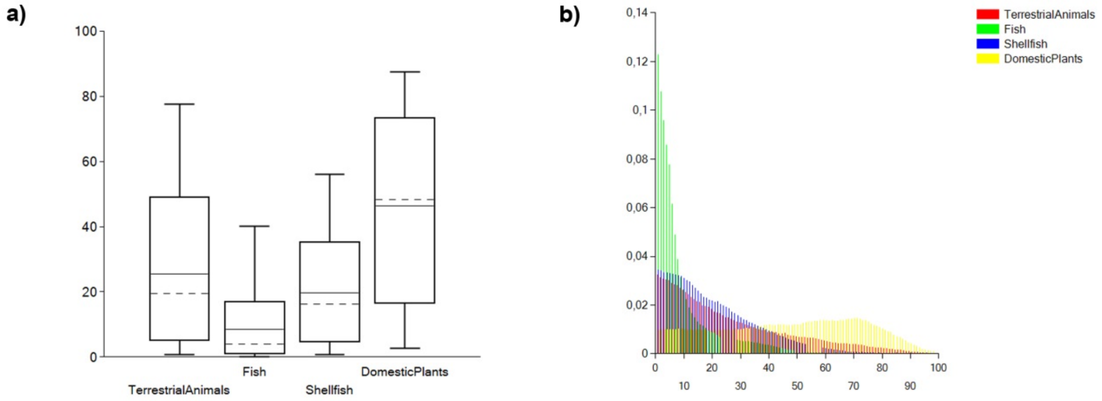

Figure S14. M8 FRUITS results. a) Box plots. b) Probably distributions.

Figure S15. M9

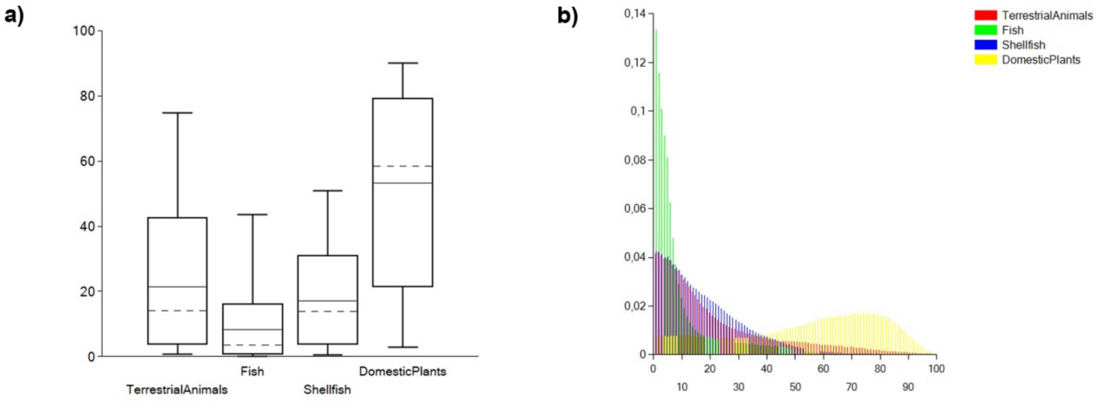

Figure S15. M9 FRUITS results. a) Box plots. b) Probably distributions.

Figure S16. M10

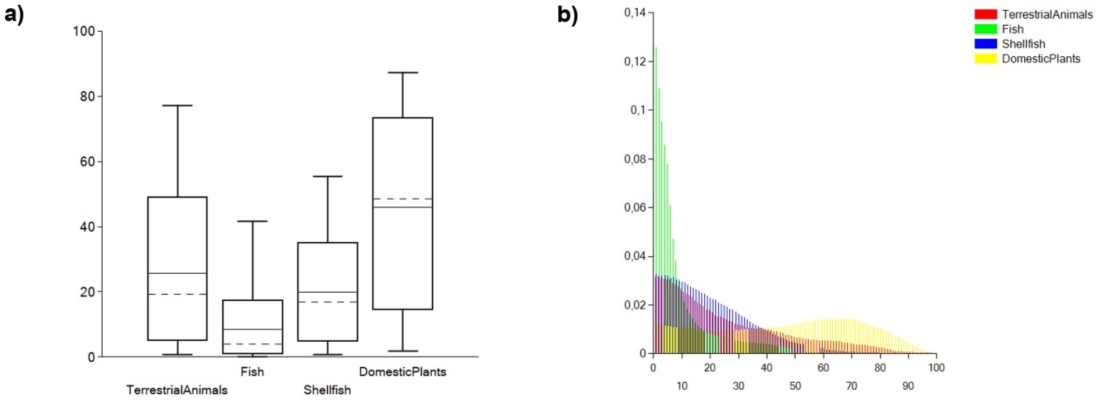

Figure S16. M10 FRUITS results. a) Box plots. b) Probably distributions.

Figure S17. M11

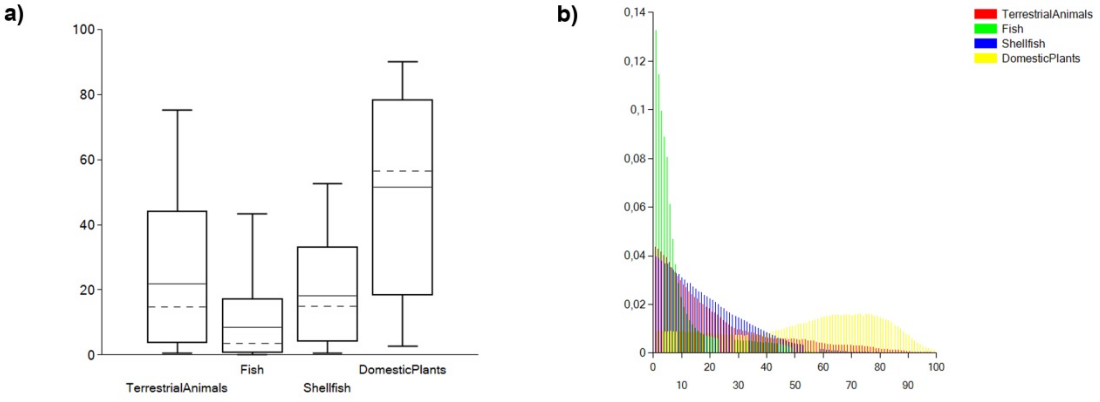

Figure S17. M11 FRUITS results. a) Box plots. b) Probably distributions.

Figure S18. M12

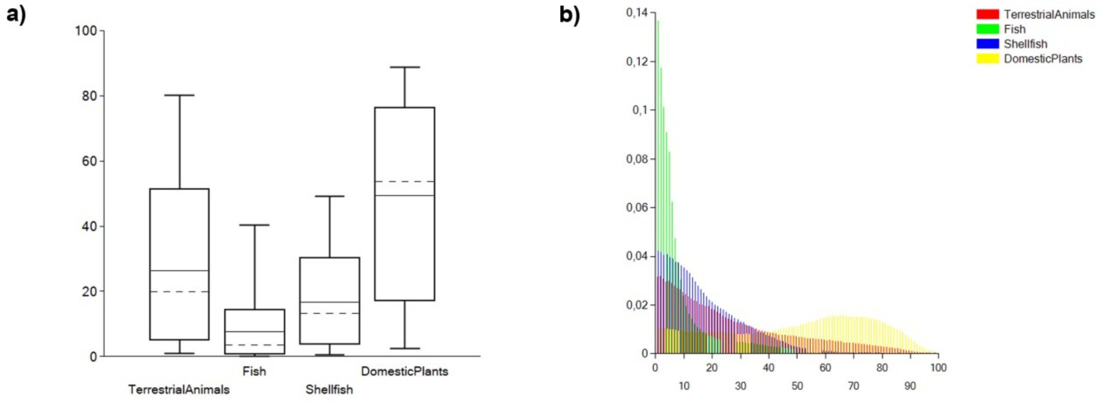

Figure S18. M12 FRUITS results. a) Box plots. b) Probably distributions.

Figure S16. M14

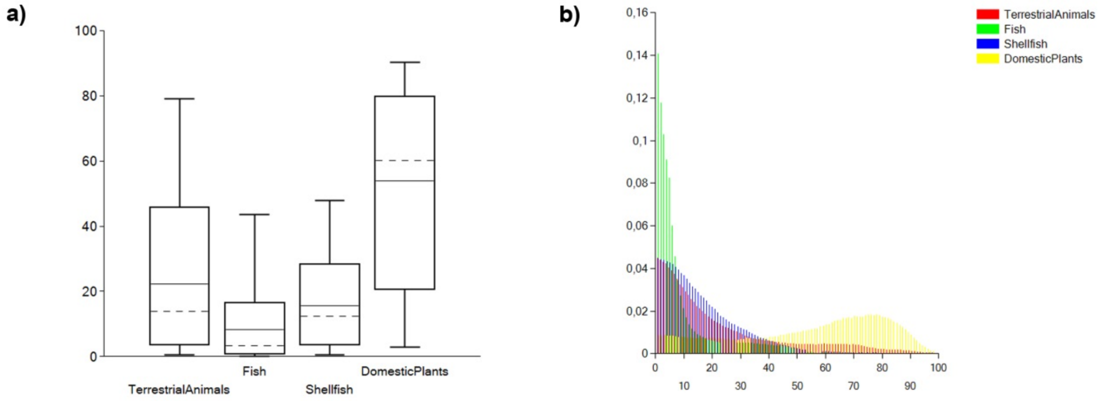

Figure S19. M14 FRUITS results. a) Box plots. b) Probably distributions.

Figure S20. M15

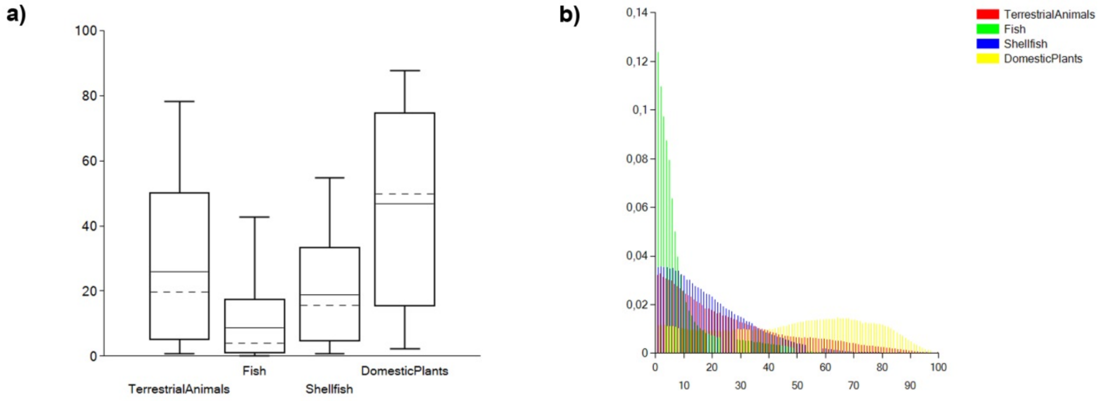

Figure S20. M15 FRUITS results. a) Box plots. b) Probably distributions.

Figure S21. M16

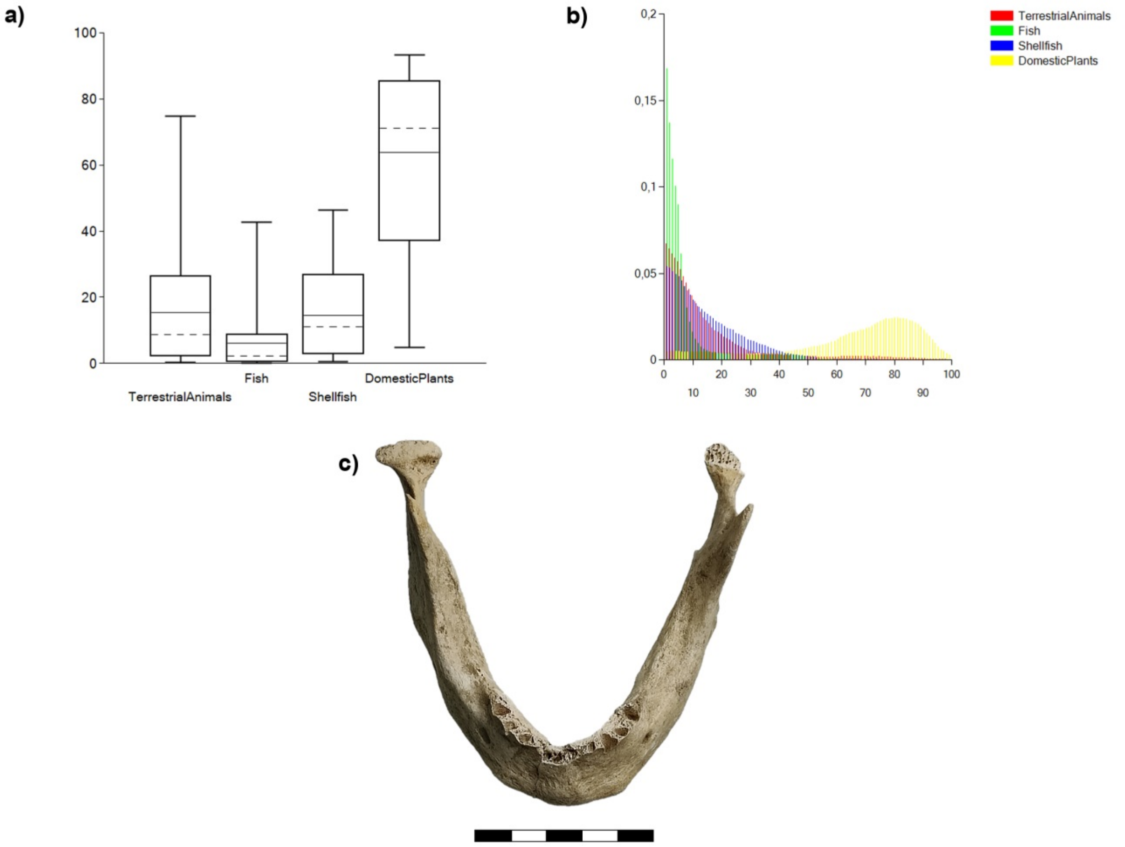

Figure S21. M16 FRUITS results. a) Box plots. b) Probably distributions. c) Mandible of M16 with antemortem loss of molars.

Figure S22. C12-I1

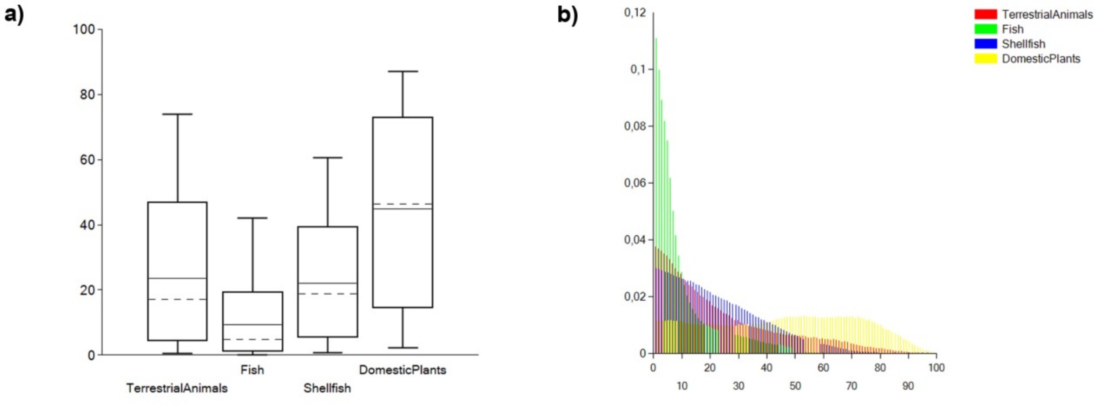

Figure S22. C12-I1 FRUITS results. a) Box plots. b) Probably distributions.

Figure S23. C12-I3

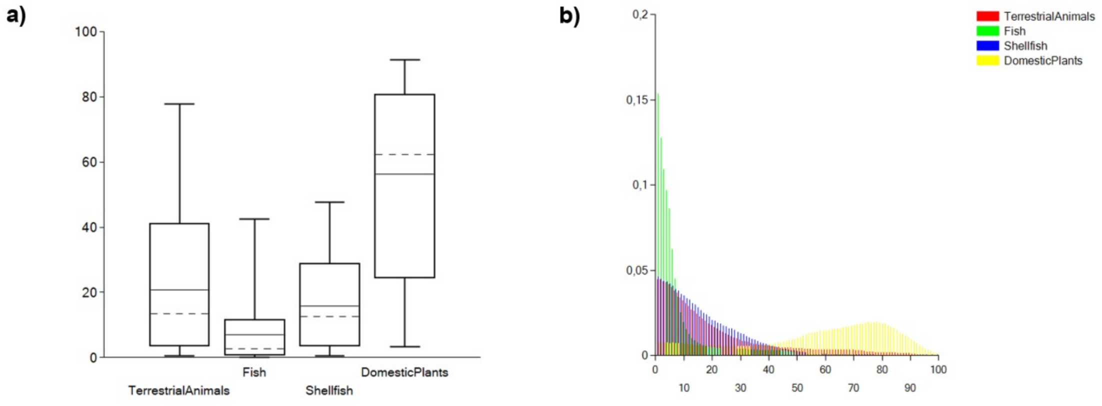

Figure S23. C12-I3 FRUITS results. a) Box plots. b) Probably distributions.

Figure S24. C12-I4

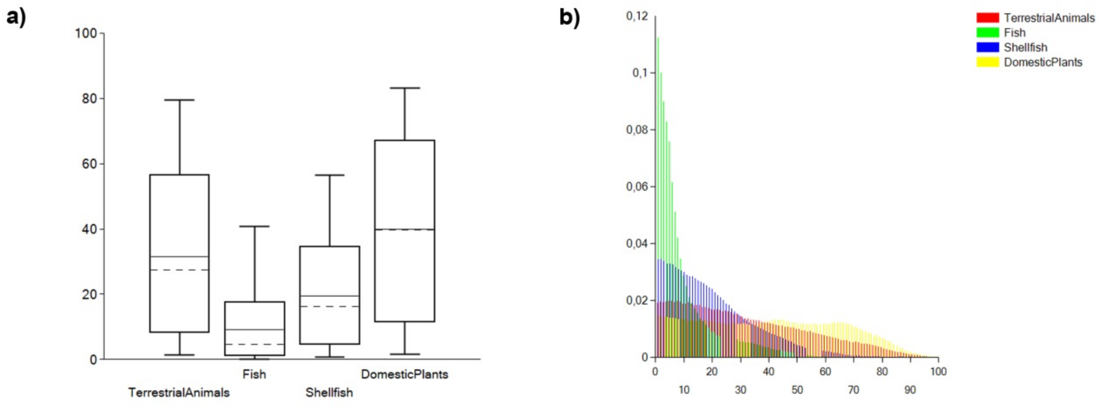

Figure S24. C12-I4 FRUITS results. a) Box plots. b) Probably distributions.

Figure S25. M17

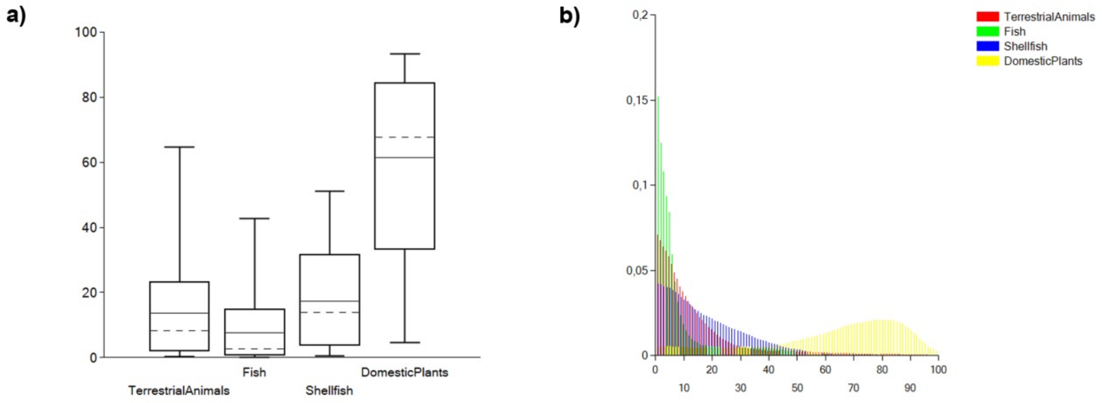

Figure S25. M17 FRUITS results. a) Box plots. b) Probably distributions.

Figure S26. M18

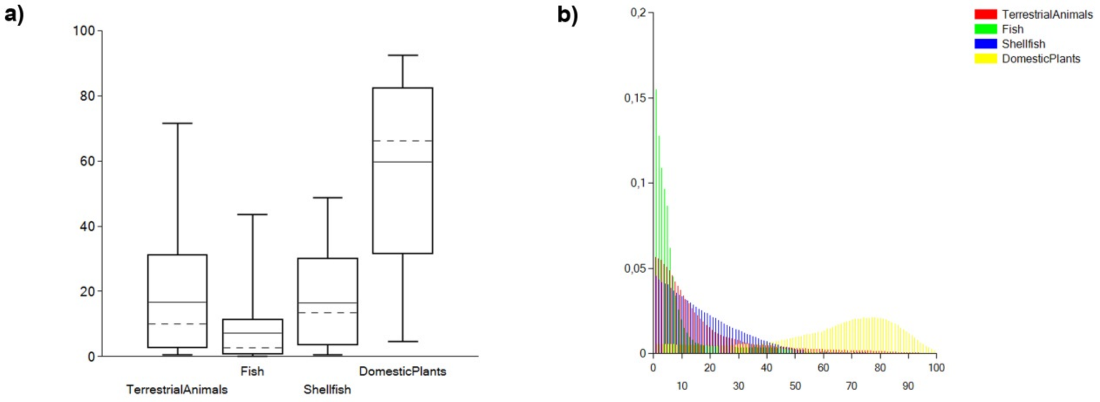

Figure S26. M18 FRUITS results. a) Box plots. b) Probably distributions.

Figure S27. M21

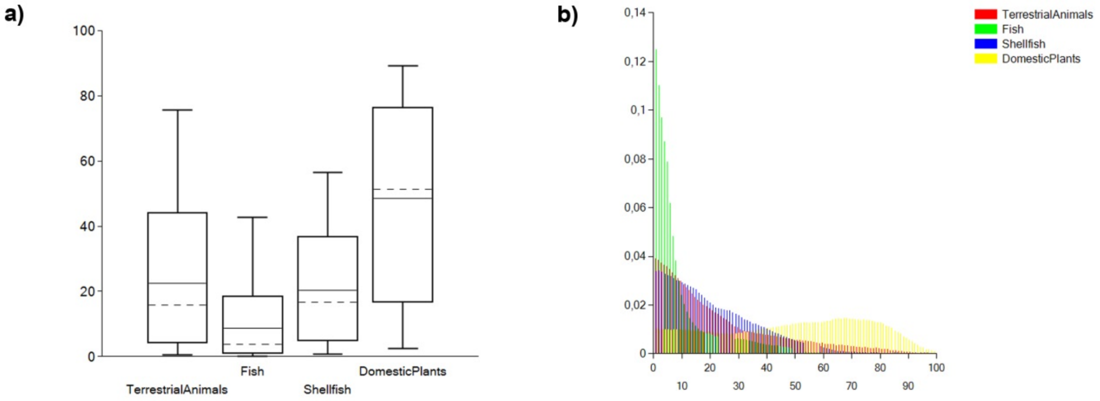

Figure S27. M21 FRUITS results. a) Box plots. b) Probably distributions.

Figure S28. M22

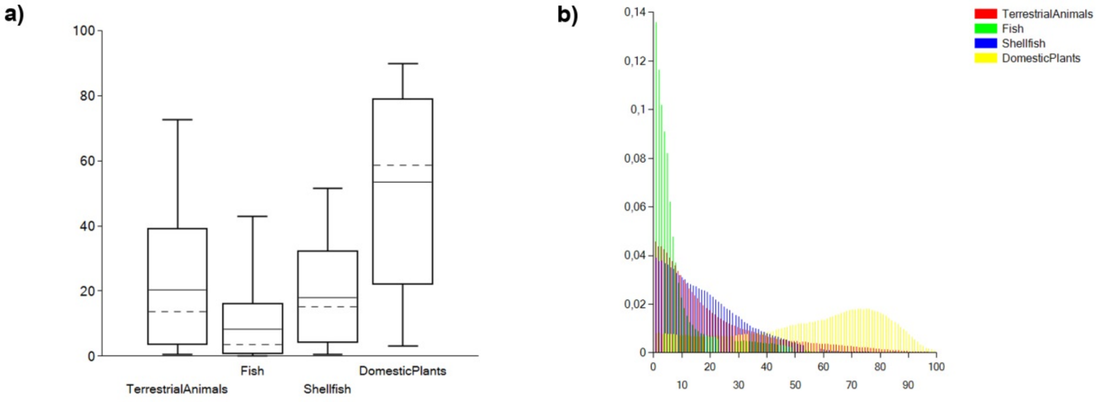

Figure S28. M22 FRUITS results. a) Box plots. b) Probably distributions.

Figure S29. M1-I2/1960

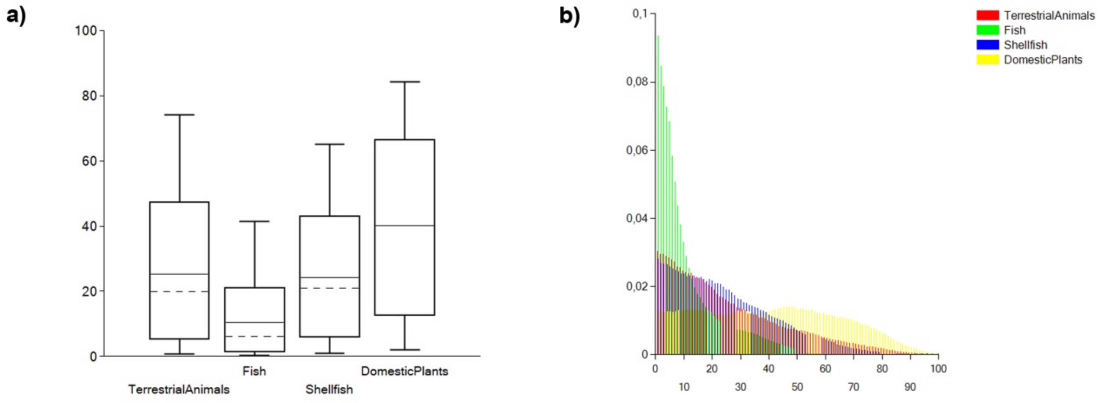

Figure S29. M1/1961 FRUITS results. a) Box plots. b) Probably distributions.

Figure S30. M1/1962

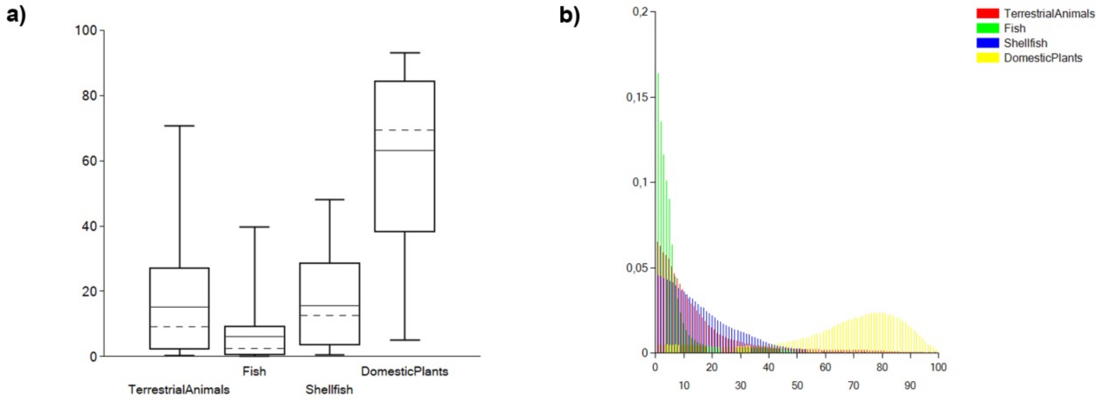

Figure S30. M1/1962 FRUITS results. a) Box plots. b) Probably distributions.

Figure S31. M2/1962

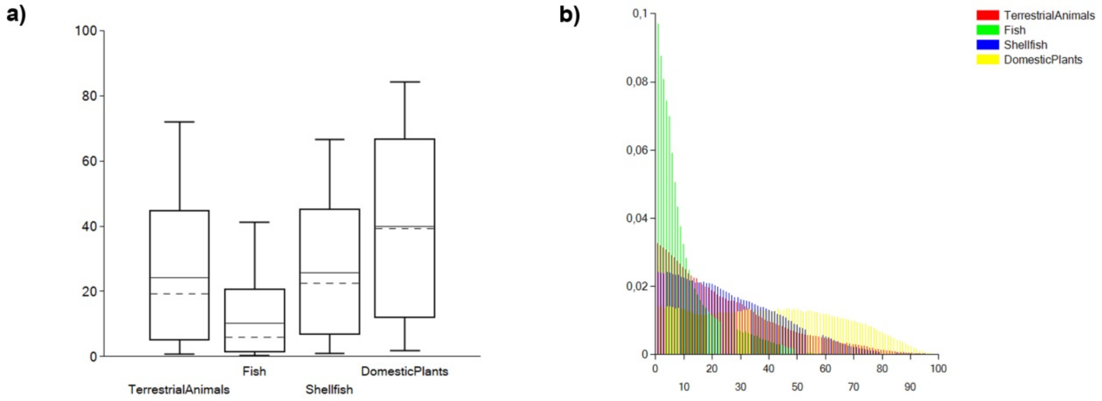

Figure S31. M2/1962 FRUITS results. a) Box plots. b) Probably distributions.

Figure S32. M3/1962

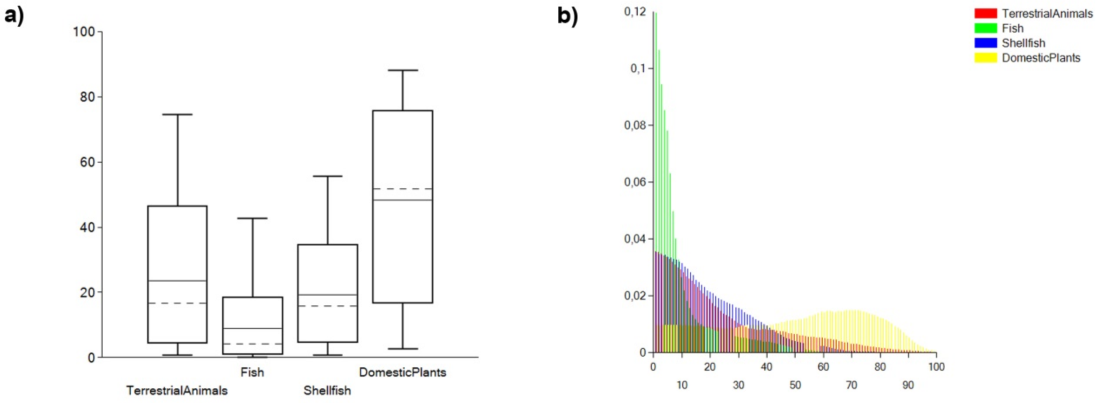

Figure S32. M3/1962 FRUITS results. a) Box plots. b) Probably distributions.

Section 6. Radiocarbon datings

Table S8. Radiocarbon datings and reservoir effect

Table S8. Radiocarbon datings from the Gumelnița tell (Zone 1) and the cemetery area (Zone 3) and the freshwater reservoir effect (FRE). * This work.

Radiocarbon Lab ID	Grave/Pit	Zone	Excavation year	Material	Date BP ± SD	Date CalBP (mean) ± SD (no FRE)	CalBC 2σ	FRE	Date CalBP (mean) ± SD (FRE)	CalBC 2σ (FRE)	Ref.
Poz-52503	M3	3	1962	Human bone	5625 ± 35	4446 ± 49	4536 - 4362 BC (95.4%)	117 ± 87	4349 ± 109	4712 - 3964 BC (95.4%)	[1]
RoAMS-655.4	M1 (ind 1)	3	2017	Human bone	5527 ± 43	4383+44	4454 - 4326 BC (92.1%)<br>4286 - 4266 BC (3.3%)	118 ± 88	4225 ± 113	4608 - 3903 BC (92.7%)<br>3880 - 3799 BC (2.7%)	[1]
RoAMS-1321.110	M2 (ind 1)	3	2018	Human bone	5437 ± 37	4288+44	4352 - 4238 BC (93.8%)<br>4186 - 4176 BC (1.6%)	-	-	-	[2]
RoAMS-1322.110	M2 (ind 2)	3	2018	Human bone	5455 ± 36	4301+39	4358 - 4244 BC (95.4%)	-	-	-	[2]
RoAMS-1325.110	M3-I2	3	2018	Human bone	5456 ± 38	4301+42	4362 - 4240 BC (95.4%)	-	-	-	[2]
RoAMS-1326.110	M4	3	2018	Human bone	5429 ± 39	4281+51	4354 - 4230 BC (90.9%)<br>4194 - 4168 BC (4.6%)	122 ± 90	4142 ± 111	4456 - 3708 BC (95.2%)<br>3670 - 3662 BC (0.3%)	[2]
RoAMS-1327.110	M5	3	2018	Human bone	5545 ± 37	4394+36	4452 - 4337 BC (95.4%)	118 ± 88	4243 ± 113	4612 - 3792 BC (95.4%)	[2]
RoAMS-1328.110	M6	3	2018	Human bone	5485 ± 38	4331+47	4444 - 4418 BC (7.5%)<br>4402 - 4312 BC (59.1%)<br>4302 - 4251 BC (28.8%)	137 ± 96	4172 ± 113	4540 - 3792 BC (95.3%)<br>3786 - 3781 BC (0.1%)	[2]
RoAMS-1330.110	M8	3	2018	Human bone	5497 ± 38	4348+48	4446 - 4318 BC (77.9%)<br>4295 - 4256 BC (17.6%)	151 ± 100	4171 ± 116	4544 - 3769 BC (95.4%)	[2]
RoAMS-1332.110	M10	3	2018	Human Bone	5646 ± 36	4472+50	4548 - 4362 BC (95.4%)	154 ± 100	4322 ± 126	4712 - 3976 BC (95.4%)	[2]
RoAMS-5111.1	M15	3	2019	Human bone	5490 ± 36	4338+46	4444 - 4418 BC (8.9%)<br>4402 - 4317 BC (63.2%)<br>4297 - 4254 BC (23.3%)	148 ± 100	4166 ± 114	4540 - 3762 BC (95.3%)<br>3724 - 3718 BC (0.2%)	*
RoAMS-5112.1	M16	3	2019	Human bone	5364 ± 34	4202+81	4330 - 4280 BC (21.5%)<br>4274 - 4216 BC (25.4%)<br>4206 - 4158 BC (22.2%)<br>4136 - 4054 BC (26.3%)	112 ± 88	4094 ± 118	4436 - 4429 BC (0.2%)<br>4364 - 3704 BC (94.4%)<br>3674 - 3656 BC (0.8%)	*
RoAMS-1335.110	C7 S.U. T1039	3	2018	Cattle bone	6040 ± 36	4935+58	5041 - 4836 BC (95.4%)	-	-	-	[2]
RoAMS-1334.110	C6, S.U. T1032	3	2018	Ovicaprine bone	5635 ± 35	4458+49	4542 - 4436 BC (66.6%)<br>4430 - 4363 BC (28.9%)	-	-	-	[2]

RoAMS-1624.122	C7, S.U. T1039	3	2018	Seeds	5954 ± 37	4834+55	4935 - 4726 BC (95.4%)	-	-	-	[2]
Poz-52502	M1 (ind1)	1	1960	Human bone	5495 ± 35	4345+46	4444 - 4416 BC (11.1%)<br>4404 - 4319 BC (66.2%)<br>4295 - 4256 BC (18.1%)	-	-	-	[2]
RoAMS-656.4	Dwelling 2 S.U.1032	1	2017	Ovicaprine bone	5560 ± 39	4401+36	4484 - 4480 BC (0.8%)<br>4456 - 4340 BC (94.6%)	-	-	-	[1]
RoAMS-657.4	Pit C6 S.U.1031	1	2017	Cattle bone	5556 ± 41	4400+38	4487 - 4478 BC (1.4%)<br>4458 - 4336 BC (94.0%)	-	-	-	[1]
RoAMS-658.4	Pit C2 S.U.1013	1	2017	Cattle bone	5582 ± 36	4410 ± 37	4493 - 4472 BC (5.2%)<br>4461 - 4346 BC (90.3%)	-	-	-	[1]
RoAMS-659.4	Pit C5 S.U.1030	1	2017	Cattle bone	5538 ± 37	4391 ± 37	4450 - 4334 BC (95.4%)	-	-	-	[1]
RoAMS-1336.110	Pit C9 S.U.1041	1	2018	Cattle bone	5379 ± 29	4234 ± 75	4333 - 4224 BC (65.9%)<br>4200 - 4164 BC (17.4%)<br>4128 - 4111 BC (2.7%)<br>4100 - 4060 BC (9.3%)	-	-	-	[2]
RoAMS-1337.110	Pit C12 S.U.1048	1	2018	Cattle bone	5472 ± 29	4315 ± 36	4362 - 4314 BC (56.4%)<br>4300 - 4252 BC (39.0%)	-	-	-	[2]
GrN-3025	Tell	1	1960's?	Charcoal	5700 ± 70	4547 ± 86	4708 - 4440 BC (87.8%)<br>4424 - 4367 BC (7.6%)	-	-	-	[17]
GrN-3028	Tell	1	1960's?	Grains	5400 ± 90	4219 ± 106	4442 - 4420 BC (1.8%)<br>4396 - 4382 BC (0.9%)<br>4370 - 4039 BC (90.7%)<br>4020 - 3992 BC (2.1%)	-	-	-	[17]
RoAMS-1616.122	C6, S.U. 1031	1	2017	Seeds	5809 ± 43	4659 ± 60	4783 - 4745 BC (7.3%)<br>4731 - 4546 BC (88.1%)	-	-	-	[2]
RoAMS-1617.122	C4, S.U. 1027	1	2017	Seeds	5660 ± 57	4495 ± 73	4672 - 4668 BC (0.3%)<br>4659 - 4636 BC (2.3%)<br>4616 - 4356 BC (92.8%)	-	-	-	[2]
RoAMS-1619.122	L1, S.U. 1026	1	2017	Seeds	5552 ± 49	4399 ± 44	4495 - 4329 BC (95.4%)	-	-	-	[2]
RoAMS-1622.122	L1, S.U. 1026	1	2017	Seeds	5482 ± 32	4327 ± 41	4442 - 4421 BC (4.4%)<br>4392 - 4389 BC (0.3%)<br>4370 - 4314 BC (60.7%)<br>4300 - 4252 BC (30.1%)	-	-	-	[2]
RoAMS-1625.122	C6, S.U. 1031	1	2018	Seeds	5464 ± 38	4308 ± 43	4439 - 4426 BC (1.6%)<br>4365 - 4242 BC (93.8%)	-	-	-	[2]

References

1. Lazăr, C. *et al.* Gumelnița: Then and now. the research results of the 2017 fieldwork. *Stud. Preist.* **2017**, 119–174 (2017).

2. Lazăr, C. *et al.* Gumelnița: Research results of the 2018 and 2019 fieldwork seasons. *Rev. Cercet. Arheol. şi Numis.* **6**, 13–100 (2020).

3. Bocherens, H. *et al.* Isotopic biogeochemistry (13C,15N) of fossil vertebrate collagen: application to the study of a past food web including Neandertal man. *J. Hum. Evol.* **20**, 481–492 (1991).

4. Balasse, M. *et al.* Wild, domestic and feral? Investigating the status of suids in the Romanian Gumelniţa (5th mil. cal BC) with biogeochemistry and geometric morphometrics. *J. Anthropol. Archaeol.* **42**, 27–36 (2016).

5. Ambrose, S. H. Preparation and characterization of bone and tooth collagen for isotopic analysis. *J. Archaeol. Sci.* **17**, 431–451 (1990).

6. DeNiro, M. J. Post-mortem preservation and alteration of in vivo bone collagen isotope ratios in relation to paleodietary reconstruction. *Nature* **317**, 806–809 (1985).

7. Schwarcz, H. P. & Nahal, H. Theoretical and observed C/N ratios in human bone collagen. *J. Archaeol. Sci.* **131**, 105396 (2021).

8. van Klinken, G. J. Bone Collagen Quality Indicators for Palaeodietary and Radiocarbon Measurements. *J. Archaeol. Sci.* **26**, 687–695 (1999).

9. Fernandes, R. A Simple(R) Model to Predict the Source of Dietary Carbon in Individual Consumers. *Archaeometry* **58**, 500–512 (2016).

10. Bownes, J. M., Ascough, P. L., Cook, G. T., Murray, I. & Bonsall, C. Using Stable Isotopes and a Bayesian Mixing Model (FRUITS) to Investigate Diet at the Early Neolithic Site of Carding Mill Bay, Scotland. *Radiocarbon* **59**, 1275–1294 (2017).

11. Pickard, C. & Bonsall, C. Post-glacial hunter-gatherer subsistence patterns in Britain: dietary reconstruction using FRUITS. *Archaeol. Anthropol. Sci.* **12**, 142 (2020).

12. Fernandes, R., Grootes, P., Nadeau, M.-J. & Nehlich, O. Quantitative diet reconstruction of a Neolithic population using a Bayesian mixing model (FRUITS): The case study of Ostorf (Germany). *Am. J. Phys. Anthropol.* **158**, 325–340 (2015).

13. Fernandes, R., Nadeau, M.-J. & Grootes, P. M. Macronutrient-based model for dietary carbon routing in bone collagen and bioapatite. *Archaeol. Anthropol. Sci.* **4**, 291–301 (2012).

14. USDA. Nutrient Database. https://ndb.nal.usda.gov/.

15. Fernandes, R., Millard, A. R., Brabec, M., Nadeau, M.-J. & Grootes, P. Food Reconstruction Using Isotopic Transferred Signals (FRUITS): A Bayesian Model for Diet Reconstruction. *PLoS One* **9**, e87436 (2014).

16. Cook, G. T. *et al.* Problems of dating human bones from the Iron Gates. *Antiquity* **76**, 77–85 (2002).

17. Bem, C. Noi propuneri pentru o schiţă cronologică a eneoliticului românesc. *Pontica* **33**–**34**, 25–121 (2000).
